# Neurofibromatosis-Noonan syndrome: a prospective monocentric study of 26 patients and literature review

**DOI:** 10.1186/s13023-025-03706-3

**Published:** 2025-04-27

**Authors:** Didier Bessis, Dominique Vidaud, Pierre Meyer, Laurence Pacot, de La Villeon G, Adeline Alice Bonnard, Yline Capri, Christine Coubes, Fanchon Herman, Didier Lacombe, Nicolas Molinari, Laura Poujade, Agathe Roubertie, Julien Van Gils, Alain Verloes, David Geneviève, Hélène Cavé, Marjolaine Willems

**Affiliations:** 1 Department of Dermatology and Reference Center for Rare Skin Diseases, Filière Maladies Rares Dermatologiques (FIMARAD), MAGEC-Sud Montpellier, Saint-Eloi Hospital and Univ Montpellier, Montpellier, France; 2 Fédération de Génétique et Médecine Génomique, Hôpital Cochin, DMU BioPhyGen, AP-HP, Centre-Université Paris Cité, Paris, France; 3 Institut Cochin, Inserm U1016, CNRS UMR8104, Université Paris Cité, CARPEM, Paris, France; 4 Department of Pediatric Neurology, Gui de Chauliac Hospital and Univ Montpellier, Montpellier, France; 5 Department of Pediatric and Congenital Cardiology, M3C Regional Reference Center, Univ Montpellier, Montpellier, France; 6 Department of Genetic Biochemistry, Robert-Debré Hospital, AP-HP and University of Paris-Diderot, Paris, France; 7 Department of Clinical Genetics and Reference Center, Reference Center for Developmental Anomalies and Malformative Syndromes- Île de France, Robert-Debré Hospital, AP-HP and University of Paris-Diderot, Paris, France; 8 Department of Clinical Genetics, Arnaud de Villeneuve Hospital, and Univ Montpellier, Montpellier, France; 9 Department of Medical Information, Epidemiological and Clinical Research Unit, La Colombière Hospital, University of Montpellier, Montpellier, France; 10 Department of Clinical Genetics, Pellegrin University Hospital of Bordeaux, AP-HP, Paris, France; 11 ERN ITHACA Coordinating Center, Paris, France; 12 Institute for Neurosciences of Montpellier, Univ Montpellier, INSERM, Montpellier, France

**Keywords:** Neurofibromatosis type 1, Noonan syndrome, NF1, RASopathies, Cardiovascular malformation

## Abstract

**Background:**

Data on clinical manifestations of neurofibromatosis-Noonan syndrome (NF-NS) remain heterogeneous, with limited validated descriptions.

**Methods:**

This study aims to better define the clinical and molecular features of NF-NS and compare them with existing literature. Secondary objectives include evaluating inter-rater diagnostic agreement among experienced clinicians and assessing the utility of deep-learning algorithms (Face2Gene^®^ [F2G]). Additionally, we assess the prevalence of congenital heart malformations (CHM) in NF-NS compared to ‘classic’ neurofibromatosis type 1 (NF1). A 9-year, prospective, monocentric study was conducted, involving patients with *NF1* pathogenic variants (PVs) and Noonan syndrome-like facial phenotype (NSLFP).

**Results:**

Twenty-six patients were enrolled. NSLFP was categorized as ‘suggestive’ in 69% of cases and ‘typical’ in 31%. The presence of at least two facial abnormalities (e.g., low-set ears, downslanted palpebral fissures, hypertelorism, and ptosis) was consistently observed in ‘typical’ cases. Inter-rater concordance was substantial (0.65 [95% CI = 0.56; 0.74]), while concordance between clinicians and F2G was almost perfect at (0.821 [CI 95% = 0.625; 1.000]). Missense *NF1* PVs were observed in 38.5% of cases. Apart from NSLP and a high frequency of pectus excavatum (62.5%), no significant differences in anthropometric, dermatological, neurological, skeletal, or ocular clinical features were observed between NF-NS and ‘classic’ NF1. CHM were found in 19.2% of NF-NS patients, with pulmonic stenosis present in 7.7%.

**Conclusion:**

NF-NS is a distinct phenotypic variant of NF1, marked by NSLP with consistent facial features -, and frequent pectus excavatum. F2G demonstrated high diagnostic concordance, reinforcing its clinical utility. Given the elevated risk of CHM, especially pulmonic stenosis, proactive cardiovascular assessment similar to other RASopathies is recommended for NS-NF patients, regardless of *NF1* PV type.

**Supplementary Information:**

The online version contains supplementary material available at 10.1186/s13023-025-03706-3.

## Introduction

Neurofibromatosis-Noonan syndrome (NF-NS; OMIM # 601321) is a rare autosomal-dominant disorder, first described by Allanson et al. in 1985 [1]. It presents with clinical features overlapping those of neurofibromatosis type 1 (NF1; OMIM # 162200) and Noonan syndrome (NS; OMIM # 163950). Approximately 320 cases identified as NF-NS have been reported (Table S1) [2–52]. Molecular studies of the *NF1* gene in NF-NS, initiated in 2005, has established that NF-NS is consistently a phenotypic variant of NF1, characterized by a high prevalence of missense or in-frame deletions of pathogenic variants (PVs) in the *NF1* gene.

However, the literature on NF-NS remains heterogeneous, mainly due to the lack of validated diagnostic criteria. Consequently, diagnosis has often been based on case reports or studies conducted under non-comparable conditions. Diagnosis were made based on: (*i*) the presence of ‘Noonan syndrome-like’ facial phenotype (NSLFP), often without detailed clinical descriptions; (*ii*) other NS diagnostic criteria, such as short stature, pectus deformity, or pulmonic valve stenosis - features also commonly reported in ‘classic’ NF1; or (*iii*) in nearly half of all cases, large NF1 cohort studies with genotype-phenotype correlations linked to recurrent PVs, including p.(Met992del) in-frame deletion [23], p.(Met1149) [24], p.(Arg1809) [31, 38], p. (Arg1038) [22, 45], p.(Arg1276) [24], p.(Lys1423) [24], and codons 844–848 missense PVs [22]. Moreover, cases with PVs such as p.(Arg1276) [24], p.(Lys1423) [24], and p.(Arg1809) [31] missense PVs appear to be associated with a higher prevalence of congenital heart malformations (CHM), including pulmonic valvular stenosis (PVS), compared to “classic” NF1.

Given these diagnostic challenges, it is important to clarify whether specific clinical features, such as CHM, are more prevalent in NF1 patients with NSLFP compared to ‘classic’ NF1. This also raises the question of whether systematic cardiovascular evaluations, including follow-up by a cardiologist with echocardiography, should be recommended in NF1 with NSLFP, similar to guidelines for other RASopathies such as NS [53], cardiofaciocutaneous syndrome [54], and Costello syndrome [55].

In this French monocentric and multidisciplinary prospective study, our primary objective was to better define the clinical manifestations of NF-NS by studying a cohort of children and adults with molecularly confirmed *NF1* PVs. Our secondary objectives were twofold: (*i*) to evaluate inter-rater agreement among clinicians experienced in diagnosing NF-NS and assess the effectiveness of phenotypic evaluation; and (*ii*) to determine whether there is an increased risk of CHM in NF-NS patients, regardless of the type of *NF1* PV, compared to those with ‘classic’ NF1.

## Patients and methods

We prospectively enrolled children and adults with suspected NF-NS, evaluated at the Reference Center for Rare Skin Diseases and departments of medical genetics and pediatric neurology at CHU Montpellier, Montpellier, France, from March 2013 to December 2022.

This study was approved by the Clinical Research Department of the University Hospital (DB, MW) and relevant ethics committees. Informed consent was obtained from all participants or their legal guardians.

### Inclusion and evaluation criteria

Patients were included if they had a clinically confirmed diagnosis of NF1, based on NIH diagnostic criteria (1988 and revised in 2021) [56, 57], and a confirmed *NF1* PV, along with NSLFP. Each patient underwent a thorough clinical assessment, including family history, physical exams, and evaluations of the cutaneous, neurological, ophthalmological, skeletal, and cardiac systems.

### Facial phenotype analysis

NSLFP was assessed by a dermatologist and geneticists (DB, DG and MW), considering the following NSLFP features: coarse facial features, flat occiput/brachycephaly, facial asymmetry, prominent and high forehead, frontal bossing, ptosis, hypertelorism (interpupillary distance > 2 standard deviations), midface hypoplasia, triangular face, downslanted palpebral fissures, eversion of the lateral eyelid, thickened eyelids, epicanthal folds, low-set posteriorly angulated ears, thickened upper helix, high and broad nasal bridge, depressed flat nasal root, bulbous and upturned nasal tip, hooked nose, wide and prominent philtrum, wide peaks to vermillion border of the upper lip (cupid’s bow appearance), micrognathia, and a small, pointed chin.

Standardized 2D facial images, including both front-facing and profile views, were taken during clinical visits using standard digital photography. To ensure a natural facial gesture, images were acquired in an upright position with a neutral facial expression. All photographic images were reviewed separately by a team of geneticists (MW, DG, DL, JVG, AV, YC) and dermatologist (DB). NSLFP was rated according to the following classification: typical (scored 2), suggestive (scored 1), and low-suggestive (scored 0).

Frontal images were analyzed using the Face2Gene^®^ (F2G) tool (FDNA Inc., Boston MA, USA, v.19.1.7) without any additional molecular or clinical information provided [58]. F2G is a clinical decision support tool that leverages machine learning to assist in the diagnosis of genetic syndromes. By analyzing facial photographs, the software compares the patient’s facial features to known genetic syndromes and generates a differential diagnosis listing the top 30 syndrome matches. For each syndrome, the software evaluates the images by creating a heat-map based on the Gestalt score confidence, categorizing the results as “high” (considered typical, scored 2), “medium” (considered suggestive, scored 1), or “low” (considered low-suggestive, scored 0).

### Genetic screening

Genetic screening of genes known to be involved in RASopathies (i.e. *PTPN11*, *SOS1*, *SOS2*, *SHOC2*, *CBL*, *HRAS*, *NRAS*, *KRAS*, *RIT1*, *RRAS*, *RRAS2*, *BRAF*, *RAF1*, *MAP2K1*, *MAP2K2*, *SPRED1*, *SPRED2*, *NF1*, *PPP1CB*, and *LZTR1*) [59] was performed by next-generation sequencing (NGS) on genomic DNA obtained from peripheral leukocytes. Briefly, NGS was performed using capture-based target enrichment (Custom SureSelect XTHS2, Agilent) and sequencing on a NextSeq500^®^ (High Output Kit v2, 2*150 bp) or NextSeq2000^®^ (Flow Cell P2, 2*150 bp) (Illumina). Bioinformatic alignment was performed using Pipeline Local Run Manager v.2.4.0 (Illumina). Read alignment and variant calling was performed using VarScan v.2.3.5, with the UCSC GRCh37/hg19 genome assembly version as reference. Variant classification was performed using Alissa Interpret^®^ (Agilent Technologies). The average sequencing depth was 100x. The pathogenicity of amino acid variants was interpreted according to international expert consensus [60, 61], taking into consideration the Human Gene Mutation Database (HGMD), Leiden Open Variation Database (LOVD3.0) and ClinVar information.

*NF1* variants were named according to the National Center for Biotechnology Information (NCBI) reference transcript sequence with the following GenBank accession number NF1 (NC_000017.10). Previous reports of single nucleotide variants were checked by consulting the Ensembl genome browser (http://www.ensembl.genome.org).

### Statistical analysis

Categorical variables were reported with the number of observations (N) and the frequency of each modality (%). Group comparisons were made using the Chi-squared test or Fisher’s exact test, as appropriate. *P*-values were adjusted using the false discovery rate method.

Concordance analysis including both inter-rater between one dermatologist (DB) and six geneticists (AV, DG, DL, MW, JVG, YC) and the clinicians’ panel average rating *versus* F2G analysis was performed using Gwet’s AC coefficient. The interpretation of Gwet’s AC coefficient was as follows: < 0: poor agreement; 0.01–0.20: slight agreement; 0.21–0.40: fair agreement; 0.41–0.60: moderate agreement; 0.61–0.80: substantial agreement; 0.81–1.00: almost perfect agreement. The Gwet’s AC coefficient is presented with its 95% confidence interval (CI), and a summary of the various Gwet’s AC coefficients is displayed using a Forest Plot. All statistical tests were two-sided, and *P*-values ≤ 0.05 were considered statistically significant. All statistical analyses were performed using R software, version 4.3.1.

## Results

From March 2013 to December 2022, 26 patients diagnosed with NF-NS were recruited, representing 4.7% of a cohort of 512 NF1 (NIH criteria, revised in 2021). The characteristics of these patients are summarized in Table 1 (details in Table S2). All patients were Caucasian and predominantly male (73%), with a median age of 10 years (range 1–45).

**Table 1:** Baseline characteristics and frequency of clinical manifestations end pathogenic variants *NF1* in our series in comparison with the data from the literature on neurofibromatosis type 1-Noonan syndrome and neurofibromatosis type 1

	Neurofibromatosis type 1-Noonan syndrome Our study	Neurofibromatosis type 1-Noonan syndrome literature review	Neurofibromatosis type 1 literature review	Our study vs. neurofibromatosis type 1-Noonan syndrome literature review (Corrected *P*-value)	Our study vs. neurofibromatosis type 1 literature review (Corrected *P*-value)	Neurofibromatosis type 1-Noonan syndrome literature review vs. neurofibromatosis type 1 literature review (Corrected *P*-value)
Baseline characteristics						
Number of patients	26	321	ND	-	-	-
Sex ratio	1.1 (19 M/17 F)	1.1 (159 M/142F)	ND	1	-	-
Facial Noonan phenotype	100% (26/26)	100% (242/242)	3.1% (93/2978)	1	**< 0.001**	**< 0.001**
Down-slanting palpebral fissures	53.8% (14/26)	45.9% (111/242)	ND	0.704	-	-
Low set and/or angulated ears	61.5% (16/26)	57.9% (140/242)	ND	0.925	-	-
Ptosis	37.5% (9/24)	40.1% (97/242)	9.3% (7/75)	1.000	**0.028**	**< 0.001**
Hypertelorism	50% (13/26)	61.2% (148/242)	52% (62/119)	0.470	1.000	0.114
Prominent and/or high forehead	30.8% (8/26)	10.7% (26/242)	ND	0.072	-	-
Triangle-shaped head	26.9% (7/26)	4.5% (11/242)	ND	**0.010**	-	-
Epicanthal folds	23.1% (6/26)	14.5% (35/242)	ND	1.000	-	-
Midface and/or malar hypoplasia	23.1% (6/26)	21.9% (53/242)	ND	0.469	-	-
Short stature	23% (6/26)	48.7% (134/275)	17.4% (409/2346)	0.071	0.661	**< 0.001**
Macrocephaly	42.3% (11/26)	41.2% (93/226)	31.3% (787/2516)	1	0.443	**0.003**
Cardiovascular abnormalities						
Cardiovascular malformations	19.2% (5/26)	36.8% (112/304)	4% (121/3054)	0.162	**0.019**	**< 0.001**
Pulmonic stenosis	7.7% (2/26)	24.3% (74/304)	1.7% (61/3680)	0.161	0.181	**< 0.001**
Left heart obstruction (aortic stenosis/coarctation)	3.8% (1/26)	2% (6/304)	0.3% (10/3849)	0.668	0.139	**< 0.001**
Mitral valves prolapse/dysplasia	7.7% (2/26)	5.6% (17/304)	1.1% (36/3236)	0.811	0.119	**< 0.001**
Atrial septal defect	0% (0/26)	4.3% (13/304)	0.3% (13/4646)	0.807	1	**< 0.001**
Ventricular septal defect	0% (0/26)	2% (6/304)	0.3% (11/4338)	1	1	**< 0.001**
Hypertrophic cardiomyopathy	0% (0/26)	2.6% (8/304)	0.1% (4/2932)	1	1	**< 0.001**
Electrocardiographic abnormality	8.7% (2/23)	0.7% (2/304)	0.2% (5/2322)	0.103	**0.012**	0.202
Vasculopathy						
Cerebral vasculopathy	3.8% (1/26)	0% (0/305)	4% (122/3024)	0.162	1	0
Peripheral vasculopathy	3.8% (1/26)	0.6% (2/321)	2.5% (2/77)	0.372	1	0.186
Skin manifestations						
Café au lait spots (> 5)	100% (26/26)	96% (288/300)	93.6% (3690/3943)	0.807	0.661	0.112
Skinfold freckling	96.2% (25/26)	64% (192/300)	78.9% (3022/3831)	**0.004**	0.114	**< 0.001**
Superficial cutaneous neurofibromas (> 18 y)	62.5% (5/8)	37.6% (32/85)	91% (803/882)	0.423	0.114	**< 0.001**
Subcutaneous neurofibromas (> 18 y)	50% (4/8)	14.1% (12/85)	57.7% (297/515)	0.103	0.961	**< 0.001**
Plexiform neurofibromas (major external/severe; > 8 y)	37.5% (3/8)	12.6% (23/183)	20% (169/847)	0.162	0.423	**0.024**
Neurological manifestations						
Learning disabilities	15.4% (4/26)	32.8% (98/299)	24.5% (260/1063)	0.162	0.497	**0.005**
Attention deficit hyperactivity disorders	34.6% (9/26)	7.4% (22/299)	22.8% (227/996)	**0.002**	0.372	**< 0.001**
Clinical autism spectrum disorder	0% (0/26)	1% (3/299)	7.8% (163/2077)	1	0.473	**< 0.001**
Developmental delay/intellectual disability	19.2% (5/26)	25.8% (77/299)	13.4% (154/1146)	0.712	0.632	**< 0.001**
Nervous-system tumours						
Optic pathway gliomas (RMI and/or CT-scan; < 6 y)	ND	20% (4/20)	18% (102/566)		-	0.793
Malignant peripheral nerve sheat tumours	3.8% (1/26)	0% (0/321)	3.4% (191/5682)	0.162	0.847	**< 0.001**
Lisch nodules (> 20 y)	33.3% (1/3)	51.4% (18/35)	94% (102/108)^∃^	1	0.062	**< 0.001**
Skeletal abnormalities						
Scoliosis	19.2% (5/26)	23.2% (70/302)	22.9% (240/1047)	0.811	0.904	0.926
Pectus excavatum	61.5% (16/26)	19.9% (57/287)	1% (12/1157)^φ^	**< 0.001**	**< 0.001**	**< 0.001**
NF1 pathogenic variant						
Truncating	34.6% (9/26)	18.6% (54/280)	54% (576/1067)	0.162	0.139	**< 0.001**
Missense	38.5% (10/26)	61.4% (172/280)	9.2% (98/1067)	0.102	**< 0.001**	**< 0.001**
Splice	15.4% (4/26)	3.2% (9/280)	27.3% (291/1067)	0.089	0.390	**< 0.001**
In-frame	0% (0/26)	12.5% (35/280)	2% (21/1067)	0.161	1	**< 0.001**
Large deletions	11.5% (3/26)	3.6% (10/280)	7.5% (80/1067)	0.172	0.661	**0.024**

Abbreviations: CT, computed tomography; F, female; M, male; NF1, neurofibromatosis type 1; NS, Noonan syndrome; ND, not done; RMI, resonance magnetic imaging; y, years.

### Facial phenotype analysis by clinicians

**Fig. 1:**
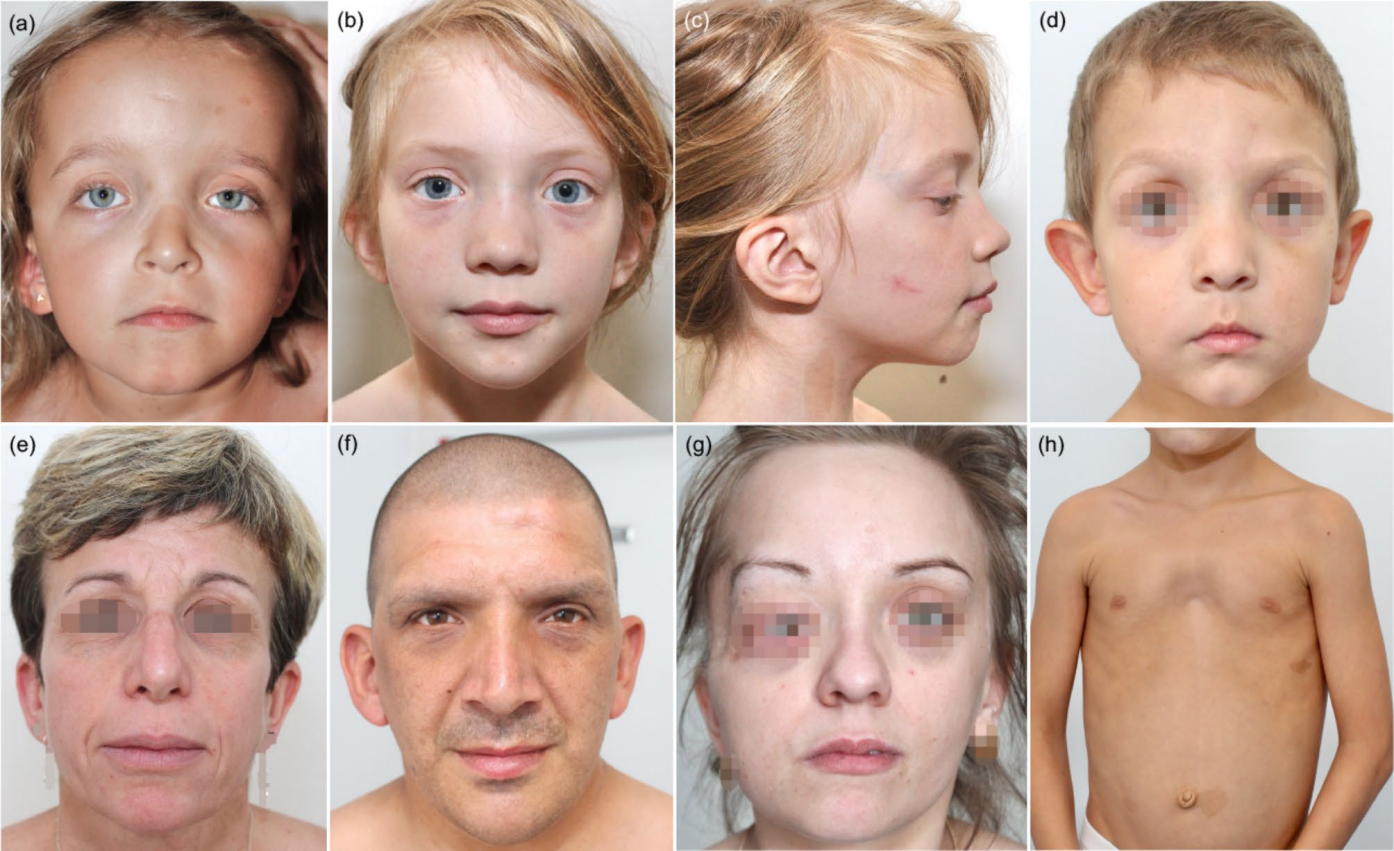
Neurofibromatosis-Noonan syndrome facial and thoracic features. (**a**) Prominent and high forehead, ptosis, hypertelorism, down-slanting palpebral fissures and low-set ears in a 5-year-old girl. (**b** and **c**) Hypertelorism, low set posteriorly angulated ears, high and broad nasal bridge, wide and prominent philtrum and triangular face in an 8-year-old girl. (**d**) Prominent and high forehead, high and broad nasal bridge, small and pointed chin in a 5-year-old-boy with recurrent p.Arg1809Cys *NF1* pathogenic variant. (**e**) Low-set ears and prominent nasolabial folds in a 45-year-old woman. (**f**) Frontal bossing, hypertelorism and prominent nasolabial folds in a 45-year-old man. (**g**) Prominent and high forehead, high anterior hairline, low-set ears and bulbous nasal tip in a 27-year-old-woman. (**h**) Pectus excavatum and café-au-lait spots in an 8-year-old boy

NSLFP was classified as ‘typical’ in 31% and ‘suggestive’ in 69% of cases. The inter-rater concordance showed substantial agreement, with a kappa of 0.65 [95% CI = 0.56; 0.74]; Suppl Fig. 1A). For sub-groups of patients aged under 12 years and 12 years or older, the inter-rater concordance showed moderate agreement, with kappa values of 0.47 [95% CI = 0.06; 0.88] and 0.73 [95% CI = 0.60; 0.87], respectively (Suppl Fig. 1B, 1 C). Common NSLFP features included low-set/angulated ears (61.5%), downslanted palpebral fissures (53.8%), hypertelorism (50%), and ptosis (37.5%). At least two of these anomalies were present in 100% of ‘typical’ cases and in 61% of ‘suggestive’ cases (Fig. 1).

### Facial analysis by Face2Gene (F2G)

F2G ranked NS as the top match in 73% of cases and second match in 15% (Table S3). NF1 was listed in the top five in 88.5% and top ten in 100%. The most common alternative diagnoses included other RASopathies, like NS with multiple lentigines, cardiofaciocutaneous syndrome, NS-like disorders with loose anagen hair, and Costello syndrome.

Concordance between the clinicians and F2G was almost perfect with a kappa of 0.821 [CI 95% = 0.625; 1.000]) (Suppl Fig. 1D).

### Cardiovascular malformations

Cardiovascular malformations were identified in 19.2% of cases, including pulmonic stenosis in 7.7%, with one case each of mild valvular and supravalvular stenosis, and mitral valve prolapse/dysplasia in 7.7%. Incomplete right bundle branch block was noted in 8.7%, and vasculopathies in 7.7%, including Moya-Moya disease with renal artery stenosis and ascending aortic dilatation.

### Additional features

Key data are summarized in Table 1, with additional details in Table S4. All patients met NF1 diagnostic criteria and café-au-lait spots were universally present. Lentigines, superficial neurofibromas, subcutaneous neurofibromas, and plexiform neurofibromas were observed in 96.2%, 62.5%, 50% and 37.5%, respectively. Neurological manifestations were present in 61.5%, with attention deficit hyperactivity disorder (ADHD), developmental delay/intellectual disability, and learning disabilities observed in 34.6%, 19.2% and 15.4%, respectively. Macrocephaly, short stature, pectus excavatum and scoliosis were present in 42.3%, 23%, 62.5% and 19.2%, respectively.

Considering the diagnostic criteria of NS established by van der Burgt [62] and by Zenker [63], 38.5% and 30.8% of the patients, respectively, could also be diagnosed with NS. All identified *NF1* PVs were classified as pathogenic/likely pathogenic and included missense, truncating, splice and large deletions, occurring in 38.5%, 34.6%, 15.4% and 11.5%, respectively. Of the 10 missense PVs, 80% were previously reported as being associated with NF-NS, specifically *NF1* p.(Arg1809) (50%, 2 families), p.(Arg1276) (20%, 2 families) and p.(Lys1423) (10%) No PVs were found in other RASopathies-related genes.

### Comparison with ‘classic’ NF1 and literature

A literature review of 321 cases NF-NS (Table *S1*) [1–52] found that 94.7% met NIH NF1criteria. The most common NSLFP features were hypertelorism (61.2%), low-set and/or angulated ears (57.9%), downslanted palpebral fissures (45.9%), and ptosis (40.1%). At least two of these four anomalies were present in 70.7% of the cases. Cardiovascular malformations were noticed in 36.8% of cases, including pulmonic stenosis in two-thirds. Scoliosis and pectus excavatum were observed in 23.2% and 19.9%, respectively.

PVs in the *NF1* gene were identified in 87.2% of cases, with missense, truncating, in-frame, large deletions, and splice variants found in 61.4%, 18.6%, 12.5%, 3.6% and 3.2%, respectively. Additionally, co-occurring PVs in other RASopathies-associated genes were noted in eight cases, including seven with *PTPN11* PVs [8, 14, 29, 33, 44] and one with a *KRAS* PV [5]. Overall, *PTPN11* PVs were identified in 4.7% of cases.

## Discussion

Our study confirms that NF-NS is a rare phenotypic variant of NF1, with a frequency of 4.7% in our cohort, consistent with the literature reports ranging from 2% to 6.4% [4, 64–67]. However, these findings are often heterogeneous due to the lack of standardized diagnostic criteria for NF-NS. We included patients with NF1 confirmed by NIH criteria and molecular analysis of *NF1* gene, who exhibited typical or suggestive facial abnormalities (“gestalt”) of NS. Molecular confirmation of the NF1 was essential to avoid misdiagnosing NF-NS as other RASopathies with overlapping features, such as café-au-lait spots and lentigines, seen in Legius syndrome [67], NS [68], NS with multiple lentigines [68], and heterozygous *LZTR1* variants [69]. At inclusion, we did not consider other NS diagnostic criteria (e.g., short stature, thoracic or cardiac malformations) but focused on NSLFP as the cornerstone of NF-NS diagnosis due to its clinical relevance and lower susceptibility to bias. NF1 lacks a distinctive facial phenotype among RASopathies [57, 70], and short stature, a common feature in 20% of ‘classic’ NF1 [14, 32, 65, 66, 71–75], lacks discriminatory value. Similarly, in ‘classic’ NF1, pectus deformities remained underexplored [76], while congenital cardiovascular malformations have been reported with frequencies ranging from 0.4 to 8.6% [74], with PVS present in 1.7% [12, 32, 77–81]. Studies on PVS are limited by small sample sizes and depend on whether the diagnosis was based on or confirmed by auscultation or echocardiography [81].

Recognizing NSLFP is challenging, as features evolve and become more subtle with age [82]. Inter-rater agreement among clinicians was moderate (κ = 0.65 [95% CI = 0.56; 0.74]) reflecting the inherent variability and subjectivity in assessing facial phenotypes. The presence of two or more facial abnormalities (e.g., low-set and/or angulated ears, downslanted palpebral fissures, hypertelorism, and ptosis) is a valuable diagnostic indicator, consistently observed in ‘typical’ NF-NS cases. According to the literature, these features are noticed in nearly three-quarters of cases. However, the specificity of these features in ‘classic’ NF1 remains undetermined in the absence of dedicated studies. Hypertelorism and ptosis have been reported in 52% [72, 83] and 9.3% [84] of ‘classic’ NF1 cases, but the small number of observations precludes definitive conclusions.

F2G analysis demonstrated high performance, ranking NS as the top match in 73% of cases and NF1 among the top five in 88.5%. Despite relying solely on front-facing images, F2G’s performance was comparable to clinicians with access to comprehensive data. Concordance between F2G and clinicians in identifying typical or suggestive NSLFP was near-perfect (κ = 0.821). While NF1 has been historically thought to lack distinct facial features, recent studies using deep learning suggest subtle facial characteristics in NF1 compared to controls [85], or within RASopathies, particularly milder CS features [70]. These technologies have limitations, including population-specific traits [85], but their precision could improve with the inclusion of clinical data or genetic information.

Our study detailed anthropometric, dermatological, neurological, ocular, and skeletal findings, which were broadly consistent with the literature. We observed a higher frequency of skinfold freckling (96.2% vs. 64%) and ADHD (34.9% vs. 7.4%), likely due to systematic data collection and evolving diagnostic criteria for ADHD. Pectus excavatum was present in 61.5%, higher than the 19.9% reported in the NF-NS literature, possibly due to our inclusion of minor cases. Aside from NSLFP and pectus excavatum, our study did not identify a distinct phenotype compared to ‘classic’ NF1, aligning with a previous detailed series of 22 NF-NS patients [16] and contrasting with other studies focusing on specific NF1 patients with *NF1* PVs [22–24, 31, 38, 45] or associated CHM [32].

CHM in ’classic’ NF1 is reported in 4% of cases, ranging from 0,4 to 6,4% with PVS occurring in 1.7% overall [12, 77–81, 86]. This may be underestimated as cardiovascular assessments often rely on auscultation [81]. Echocardiography, routinely recommended for other RASopathies [53], is not yet established for ‘classic’ NF1. The association between NF-NS and higher CHD risk, including PVS, and prevalent missense or in-frame NF1 PVs [32], such as p.(Arg1276) [24], p.(Lys1423) [24], and p.(Arg1809) [31], is supported by pooled literature data. More globally, the increased risk of CHM in NF-NS, regardless of the type of *NF1* pathogenic variant, appears to be confirmed by pooled data from the literature, with a significantly increased frequency of CHM and PVS at 36.8% and 24.3%, respectively. Our study, conducted without presupposing NF1 PV types, confirmed an increased CHM risk (19%), with a trend toward increased PVS (7.7%), left heart obstruction (3.8%), and mitral valve prolapse/dysplasia (7.7%). These findings support the need for an initial cardiac evaluation, including echocardiography, in all NF1 patients with NSLFP, regardless of the type of *NF1* PVs. Furthermore, given the potential for late-onset or progressive cardiac manifestations, we recommend periodic cardiac follow-up over time, similar to surveillance guidelines in RASopathies, even in the absence of initial cardiological abnormalities”.

*NF1* PVs remain the primary molecular event underlying NF-NS. In our cohort, the frequency of truncating PVs was higher than reported in the literature; however, we also observed a high frequency of recurrent missense PVs in 38.5%, consistent with previous findings. Additionally, RASopathy PVs, mostly linked to the *PTPN11*, were observed at a frequency of 5.7% based on pooled data [5, 8, 14, 29, 33, 44]. A recent study reported *PTPN11* PV in 2.9% of NF1 patients, 75% of whom exhibited an NS-like phenotype [14]. Although we did not identify co-occurring RASopathy PVs in our cohort, possibly due to the limited sample size, these findings support systematic screening for RASopathy PVs in NF1 patients who exhibited an NS-like phenotype.

## Conclusions

This study highlights that NF-NS is a distinct phenotypic variant of NF1, confirmed through both molecular and clinical analyses. Future advancements in facial phenotype analysis, particularly deep-learning technologies, offer promising tools for helping clinicians diagnose NF-NS earlier and more accurately. Given the increased prevalence of CHM, our findings suggest that early recognition of NSLFP in NF1 patients should prompt a more proactive cardiovascular evaluation. The frequent association of NF-NS with missense and in-frame PVs in the *NF1* gene, as well as the rare but significant co-occurrence of RASopathy PVs, underscores the importance of systematic RASopathy variant testing and genetic screening in this population.

## Electronic supplementary material

Below is the link to the electronic supplementary material.

Supplementary Material 1

Supplementary Material 2

Supplementary Material 3

Supplementary Material 4

Supplementary Material 5

Supplementary Material 6

## Data Availability

The data that support the findings are available from the corresponding author upon reasonable request.

## Declarations

This work was approved by the clinical research department of the university hospital of the principal investigator (D.B), the Consultative Committee for the Processing of Health Research Data (CCTIRS; 12.750) and the National Commission on Informatics and Liberty (CNIL; 913041).

## Abbreviations

ADHD: Attention deficit hyperactivity disorder
CHM: Congenital heart malformations
CI: Confidence interval
F: Female
F2G: Face2Gene^®^
M: Male
n: Number
NF1: Neurofibromatosis type 1
NS: Noonan syndrome
NF-NS: Neurofibromatosis-Noonan syndrome
NGS: Next-generation sequencing
NSLFP: Noonan syndrome-like’ facial phenotype
PV: Pathogenic variant
PVS: Pulmonic valvular stenosis
