# Supplementary material for: Neurofibromatosis-Noonan syndrome: a prospective monocentric study of 26 patients and literature review
Source: Orphanet J Rare Dis. 2025 Apr 27;20:201. doi: 10.1186/s13023-025-03706-3 (PMC12036184; doi:10.1186/s13023-025-03706-3)
Supplement: Supplementary file 1 — Supplementary Material 1 [file 13023_2025_3706_MOESM1_ESM.docx]

| **Table S1. Epidemiological characteristics, clinical manifestations and molecular diagnostics in series or case reports of neurofibromatosis-Noonan syndrome previously reported** | | | | | | | | | | | | | | | |  |
| --- | --- | --- | --- | --- | --- | --- | --- | --- | --- | --- | --- | --- | --- | --- | --- | --- |
| Year/Reference | Case number | NF1 family history | Sex | Age (y) | Macrocrania | Short stature | Facial Noonan phenotype | Skin manifestations | Neurological manifestations | MRI and/or CT-scan abnormalities | Cardiac manifestations | Lisch nodules | Osteoarticular manifestations | Other findings/manifestations | Molecular diagnosis | |
| 1985/Allanson (Am J Med Genet) | 1 | Sporadic | M | 19 | No | Yes | . Ptosis (bilateral)  . Midface hypoplasia  . Down-slanting palpebral fissures | PNF (right forearm), SCNF | Delayed speech development | CT-scan: tumors on the right optic nerve | No | No | . Scoliosis  . Pectus excavatum | . Impaired peripheral nerve function  . Anisocoria  . Sluggish right pupil | ND | |
|  | 2 | ND | F | 22 | Yes | Yes | . Ptosis  . Malar hypoplasia  . Down-slanting palpebral fissures  . Low posterior hairline | CLS, L, SNF (abdomen, buttocks) | . Delayed language development  . Delay motor development | ND | No | ND | . Scoliosis  . Pectus excavatum  . Cubitus valgus | Broad neck | ND | |
|  | 3 | ND | M | 5 | No | Yes | . Malar hypoplasia  . Down-slanting palpebral fissures | CLS, L, PNF (ankle) | Generalized weakness | ND | No | ND | . Scoliosis  . Pectus carinatum | . Short neck  . Type 2 fiber atrophy (electromyogram) | ND | |
|  | 4 | Sporadic | M | 1.25 | No | Yes | . Hypertelorism  . Malar hypoplasia  . Down-slanting palpebral fissures  . Low posterior hairline | CLS | Slight development delay | ND | Pulmonary valvular stenosis suspected | ND | Pectus excavatum | . Short neck with excess nuchal skin  . Increased internipple distance  . Cutaneous depigmented areas | ND | |
| 1985/Mendez (Am J Med Genet) | 5 | Sporadic | M | 12 | No | Yes | . Ptosis (bilateral)  . Hypertelorism  . Shortness of the midface  . Low-set posteriorly angulated ears  . Prominent helix and heavy lobules  . Low posterior hairline  . Scalp hair whorls  . Highly arched palate | CLS, SCNF | IQ tests 50-70 | ND | ECG normal | ND | . Scoliosis (thoracic)  . Lordosis (lumbar)  . Pectus carinatum  . Cubitus valgus | . Webbing neck  . Hypoplastic and inverted nipples  . Cryptorchidism (left)  . Chest films normal | ND | |
|  | 6 | Sporadic | F | 23 | No | Yes | . Ptosis (bilateral)  . Short midface  . Epicanthal folds  . Down-slanting palpebral fissures  . Low-set ears  . Low posterior hairline | CLS, L, SCNF | No | ND | No | No | Short 4^th^ metacarpals and short 4^th^ and 5^th^ metatarsals | Small nails | ND | |
| 1985/Opitz (Am J Med Genet) | 7 | Sporadic | M | 19 | No | No | . Malar hypoplasia  . Laterally extended eyebrows  . Highly arched palate | CLS, L, PNF | No | CT-scan normal | No | No | . Scoliosis (thoracic)  . Limitation flexion 2^nd^ and 3rd metacarpophalangeal joints | Cryptorchidism (left) | ND | |
|  | 8 | Sporadic | M | 7 | No | Yes | . Posterior angulation of the auricles  . Flat philtrum | CLS, PNF | No | ND | . No  . ECG normal | No | . Scoliosis (thoracic)  . Kyphosis (thoracic)  . Short sternum  . Clinodactyly (5^th^ fingers) | Chest films normal | ND | |
|  | 9 | Sporadic | F | 17 | No | Yes | . Ptosis  . Hypertelorism  . Posteriorly angulated auricules  . Down-slanting palpebral fissures  . Epicanthal folds (remnant upper)  . Raised arched eyebrows  . Micrognathia  . Anteversion of nostrils  . Arched palate  . Low posterior hairline | CLS, L, SNF (probable) | No | ND | No | No | Syndactyly (toes) | . Short and webbing neck  . Lymphedema (hands and toes) | ND | |
| Abbreviations: CLS, café-au-lait spots; CT-scan, computized tomography-scan; ECG, electrocardiogram; F, female; L, lentigines; M, male; MRI, Magnetic resonance imaging; ND, not done; PNF, plexiform neurofibroma; SCNF, subcutaneous neurofibroma; SNF, superficial neurofibroma | | | | | | | | | | | | | | | | |

| **Table S1. Epidemiological characteristics, clinical manifestations and molecular diagnostics in series or case reports of neurofibromatosis-Noonan syndrome previously reported (continued)** | | | | | | | | | | | | | | | |  |
| --- | --- | --- | --- | --- | --- | --- | --- | --- | --- | --- | --- | --- | --- | --- | --- | --- |
| Year/Reference | Case number | NF1 family history | Sex | Age (y) | Macrocrania | Short stature | Facial Noonan phenotype | Skin manifestations | Neurological manifestations | RMI and/or CT-scan abnormalities | Cardiac manifestations | Lisch nodules | Osteoarticular manifestations | Other findings/manifestations | Molecular diagnosis | |
| 1985/Kaplan (Am J Med Genet) | 10 | Familial | M | 17 | Yes | No | . Triangular face  . Down-slanting palpebral fissures  . Broad nose  . Thick and protruding lower lip | CLS, SNF, PNF | Visual perceptual motor deficits | CT-scan: left cerebral ventricle larger than the right | . Dilated aortic root  . Mitral valve prolapse | Yes | . Scoliosis (mild)  . Pectus excavatum  . Hexadactyly (feet)  . Larges hands and feet | . Wide neck  . Cryptorchidism  . Telecanthus | ND | |
|  | 11 | Familial | M | 7 | No | No | . Down-slanting palpebral fissures  . Eversion of lateral lower eyelids  . Broad nose  . Thick helices  . Short and pointed chin  . Arched palate  . Low posterior hairline | CLS, SNF | Mild developmental delay | CT-scan normal | . No  . ECG normal | ND | . Pectus excavatum  . Hypoplastic 12^th^ ribs  . Large hands and feet  . Cubitus valgus | . Broad neck  . Telecanthus  . Pigmented lesion of iris | ND | |
|  | 12 | Familial | F | 16 | No | No | . Down-slanting palpebral fissures  . Eversion of lateral lower eyelids  . Broad nose  . Thick helices  . Narrow pointed chin  . Arched palate  . Low posterior hairline | CLS, SNF | Dull intellect | CT-scan: mild right temporal atrophy | No | ND | . Scoliosis  . Broad distal phalanges | . Broad neck  . Telecanthus | ND | |
| 1985/Saul (Am J Med Genet) | 13 | ND | ND | 2 | ND | ND | . Hypertelorism  . Depressed nasal root  . Broad nasal tip | ND | Hypotonia (mild) | ND | Aortic stenosis | ND | ND | Hirschprung disease-like | ND | |
| 1987/ Quattrin (Am J Med Genet) | 14 | Familial | M | 3 | No | No | . Ptosis  . Hypertelorism  . Broad forehead  . Down-slanting palpebral fissures  . Epicanthal folds (upper and lower inner)  . Mid-face hypoplasia  . Cupid bow to the lip | CLS | Developmental delay  Seizures | CT-scan: left optic glioma | Echocardiography normal | No | Pectus excavatum | . Broad neck  . Left eyelid tumor  . Hypospadias  . Cryptorchidism | ND | |
|  | 15 | Familial | F | 28 | No | No | . Ptosis  . Hypertelorism  . Triangular-shaped face  . Down-slanting palpebral fissures  . Mid-face hypoplasia  . Pull-down of lower corner of palpebral fissures  . Ear lobules upturned | CLS, L, SNF | No | CT-scan: normal | ECG normal | ND | Scoliosis | . Short neck  . Dark pigmentary changes around eyes  . Chest films normal | ND | |
| 1987/Meinecke (Am J Med Genet) | 16 | Familial | M | 5 | No | Yes | . Large cranium,  . Down-slanting palpebral fissures  . Epicanthus (bilateral)  . Malar hypoplasia  . Low-set and posteriorly angulated pinnae  . Wide and depressed nasal root,  . Anteverted nostrils | CLS, L | Delayed psychomotor and speech development (slight) | ND | No | Yes | Laxity of large and small joints | . Short and wide neck  . Inverse direction of hair growth (back of the neck)  . Muscular hypotonia | ND | |

| **Table S1. Epidemiological characteristics, clinical manifestations and molecular diagnostics in series or case reports of neurofibromatosis-Noonan syndrome previously reported (continued)** | | | | | | | | | | | | | | | |  |
| --- | --- | --- | --- | --- | --- | --- | --- | --- | --- | --- | --- | --- | --- | --- | --- | --- |
| Year/Reference | Case number | NF1 family history | Sex | Age (y) | Macrocrania | Short stature | Facial Noonan phenotype | Skin manifestations | Neurological manifestations | RMI and/or CT-scan abnormalities | Cardiac manifestations | Lisch nodules | Osteoarticular manifestations | Other findings/manifestations | Molecular diagnosis | |
| 1987/Shuper (Arch Dis Child) | 17 | Sporadic | M | 12 | No | Yes | . Ptosis  . Down-slanting palpebral fissures  . Low set ears  . Arched palate  . Coarse hair  . Low posterior hairline | CLS | Learning difficulties | CT-scan: normal | Echocardiography normal | ND | . Pectus excavatum  . Cubitus valgus | . Short neck  . Pterygium coli  . Delayed puberty | ND | |
| 1988/Abuelo and Meryash (Am J Med Genet) | 18 | Familial | M | 18 | No | Yes | . Coarse face  . Ptosis  . Downslanting palpebral fissures  . Epicanthal folds (bilateral)  . Low-set and posteriorly rotated pinnae with upturned lobe  . Broad nasal bridge  . Broad and thick nasal tip  . Cupid-bow configuration of the lips  . Low posterior hairline  . Coarse and curly scalp hair | CLS, L, SNF, SCNF | Moderate retarded of intelligence | ND | . Atrial septal defect  . Valvular and supravalvular pulmonar stenosis | No | . Pectus carinatum (superior)  . Pectus axcavatum (inferior) | . Short and webbed neck  . Hydrocele (18 y)  . Inguinal hernia (8 y)  . Hearing loss (unilateral)  . Single horizontal palmar crease (right) and hypoplastic proximal crease (left) | ND | |
|  | 19 | Familial | M | 47 | Yes | No | . Low-set ears  . Prominent nasolabial folds | CLS, L, SNF, SCNF | ND | ND | No | ND | No | No | ND | |
| 1989/Borochowitz (Neurofibromatosis) | 20 | Sporadic | M | 15 | No | Yes | . Mid face hypoplasia  . Downslanting palpebral fissures  . Ptosis  . Hypertelorism  . Low set ears | CLS, L | No | CT-scan normal | . Pulmonic stenosis (cardiac catheterization)  . Left axis deviation (electrocardiogram) | No | . Pectus excavatum  . Bilateral cubitus valgus  . Short left 4^th^ metacarpal | Left undescended testes | ND | |
| 1992/Stern (J Med Genet) | 21 | Familial | M | 69 | Yes | Yes | . Ptosis  . Triangular face  . Slanted palpebral fissures  . Prominent nasolabial folds | CLS, L, SNF, PNF | Learning disability | ND | No | ND | . Scoliosis  . Decrease muscle tone | . Broad neck  . Hearing loss | NF1 variant (DNA haplotype analysis) | |
|  | 22 | Familial | M | 1 | No | Yes | . Prominent forehead  . Triangular face  . Down slanting palpebral fissures  . Low set ears | CLS | . Hypotonia  . Developmental delay | ND | No | No | No | External ear anomaly | NF1 variant (DNA haplotype analysis) | |
| 1993/Tassabehji (Am J Hum Genet) | 23 | Familial | F | 26 | ND | ND | . Low-set posteriorly rotated ears  . High nasal bridge | CLS, L | Learning difficulties (IQ=56) | ND | ND | No | ND | Squint | NF1 variant tandem duplication in frame (42 bases, exon 28) | |
|  | 24 | Familial | M | 3.5 | ND | ND | . Ptosis  . Hypertelorism  . Low-set posteriorly rotated ears  . Epicanthic folds  . High nasal bridge | CLS | Learning difficulties | CT-scan normal | Valvular pulmonary stenosis (echocardiography) | No | . Pectus carinatum  . Cubitus valgus (mild) | . Bilateral undescended testes  . Squint | NF1 variant tandem duplication in frame (42 bases, exon 28) | |
|  | 25 | Familial | F | 3.5 | ND | ND | . Hypertelorism  . Low-set posteriorly rotated ears  . Epicanthic folds  . High nasal bridge | CLS | Learning difficulties | CT-scan normal | No | No | Pectus carinatum | Squint | NF1 variant tandem duplication in frame (42 bases, exon 28) | |

| **Table S1. Epidemiological characteristics, clinical manifestations and molecular diagnostics in series or case reports of neurofibromatosis-Noonan syndrome previously reported (continued)** | | | | | | | | | | | | | | | |  |
| --- | --- | --- | --- | --- | --- | --- | --- | --- | --- | --- | --- | --- | --- | --- | --- | --- |
| Year/Reference | Case number | NF1 family history | Sex | Age (y) | Macrocrania | Short stature | Facial Noonan phenotype | Skin manifestations | Neurological manifestations | RMI and/or CT-scan abnormalities | Cardiac manifestations | Lisch nodules | Osteoarticular manifestations | Other findings/manifestations | Molecular diagnosis | |
| 1994/Kayes (Am J Hum Genet) | 26 | Sporadic | M | 20 | No | No | . Hypertelorism  . Ptosis  . Micrognathia | CLS, SNF, PNF | IQ:46 | No optic glioma | No | ND | No | Broad neck | NF1 deletion | |
|  | 27 | Sporadic | M | 25 | Yes | No | . Hypertelorism  . Micrognathia (+/-) | CLS, SNF, PNF | ND | No optic glioma | No | ND | No | No | NF1 deletion | |
|  | 28 | Sporadic | M | 5 | Yes | No | . Ptosis  . Hypertelorism  . Down-slanting palpebral fissures  . Micrognathia | CLS, SNF, PNF | Learning disability | ND | No | ND | No | Broad neck | NF1 deletion | |
|  | 29 | ND | F | 29 | No | Yes | . Ptosis  . Down-slanting palpebral fissures  . Micrognathia  . Low posterior hairline | CLS, SNF, PNF | Learning disability | No optic glioma | No | ND | . Scoliosis  . Shield chest  . Cubitus valgus  . Short fingers | Broad neck | NF1 deletion | |
|  | 30 | ND | M | 16 | Yes | Yes | . Ptosis (+/-)  . Hypertelorism  . Down-slanting palpebral fissures (+/-)  . Micrognathia | CLS, SNF, PNF | IQ59 | ND | No | ND | Scoliosis  Cubitus valgus | Broad neck | NF1 deletion | |
| 1995/Buehning (Pediatr Dermatol) | 31 | ND  (possibility of NS in patient’s mother and one sibling) | M | 25 | No | No | . Brachycephaly  . Ptosis  . Epicanthal folds  . Midfacial hypoplasia  . Double eyelashes  . Low-set ears | CLS, L, SNF, PNF | Developmental delay  Seizure | Cerebral imaging: absence of corpus callosum, bilateral optic atrophy | . Valvular pulmonar stenosis  . Dysplastic mitral valve | Yes | . Scoliosis  . Pectus excavatum  . Syndactyly of the toes  . Bilateral equinus deformities of the feet | . Broad neck  . Transverse palmar crease  . Mild conductive hearing loss  . Severe myopia  . Bilateral corneal opacities  . Visual field defects | No NF1 gene locus abnormalities | |
| 1996/Bahuau (Am J Med Genet) | 32 | Familial | M | 0.25 | Yes | Yes | . High, broad and wrinkled forehead  . Down-slanting palpebral fissures  . Small nose with anteverted nares  . Midface hypoplasia  . Low-set and posteriorly angulated ears  . Prominent anthelix, thick and detached lobule  . Micrognathia | ND | ND | CT-scan: communicating hydrocephalus | Hypertrophic cardiomyopathy | ND | . Shield chest  . Hypoplastic and excessively spaced nipples  . Short humerus and femur  . Short hands and fingers | . Short neck with excess of nuchal skin  . Pyelic dilatation of right kidney  . Mild decrease in some coagulation factors | NF1 variant (R816X)  Exclusion of allelism of Noonan syndrome (C12q) | |
| 1996/Colley (Clin Genet) | 33-45 | ND | ND | ND | ND | ND | More than 5 features of the following (see also osteoarticular manifestations):  . Ptosis  . Hypertelorism  . Epicanthic folds  . Down-slanting palpebral fissures  . Malar hypoplasia  . Low-set posteriorly rotated ears,  . Low posterior hair line | ND | ND | ND | More than 5 features of the following (see also skin and osteoarticular manifestations):  Cardiac defect | ND | More than 5 features of the following (see also skin and cardiac manifestations):  . Pectus abnormality  . Cubitus valgus  . Short stature | More than 5 features of the following (see also skin, cardiac and osteoarticular manifestations):  . Cryptorchidism  . Genital anomaly, | ND | |
| 1996/Wu (Hum Mut) | 46 | Sporadic | F | 32 | Yes | No | . Hypertelorism  . Coarse face | CLS (x4), L | Mildly mentally handicapped | CT-scan and MRI normal | . Left ventricular hypetrophy  . Aortic valvular and subvalvularstenosis | No | No | Broad neck | NF1 variant p.M1035R (exon 18) | |

| **Table S1. Epidemiological characteristics, clinical manifestations and molecular diagnostics in series or case reports of neurofibromatosis-Noonan syndrome previously reported (continued)** | | | | | | | | | | | | | | | |  |
| --- | --- | --- | --- | --- | --- | --- | --- | --- | --- | --- | --- | --- | --- | --- | --- | --- |
| Year/Reference | Case number | NF1 family history | Sex | Age (y) | Macrocrania | Short stature | Facial Noonan phenotype | Skin manifestations | Neurological manifestations | RMI and/or CT-scan abnormalities | Cardiac manifestations | Lisch nodules | Osteoarticular manifestations | Other findings/manifestations | Molecular diagnosis | |
| 1998/Bahuau (Am J Med Genet A) | 47 | Familial | ND | ND | ND | ND | . Large forehead  . Midface hypoplasia  . Downslanting palpebral fissures | ND | ND | ND | ND | ND | No | No | NF1 variant c2446T p.Arg816* (exon 16)  Exclusion of allelism of NS (C12q) | |
| 2003/Baralle (Am J Med Genet) | 48 | Sporadic | M | 20 | ND | - | . Ptosis  . Downslanting palpebral fissures | CLS, L, SNF | Need extrahelp at mainstream school | ND | Atrial septal defect | Yes | . Scoliosis  . Structural cervical vertebral abnormality | . Short/broad neck  . Widely space nipples | NF1 variant (2-bp insertion in exon 23-2, 4095 ins TG) | |
|  | 49 | ND | M | 18 | ND | + | . Ptosis  . Low-set posteriorly rotated ears | CLS, L | No | ND | . Coarctation of aorta | ND | No | No | PTPN11 not mutated | |
|  | 50 | Sporadic | M | 7 | ND | - | . Ptosis  . Hypertelorism  . Low-set posteriorly rotated ears | CLS, L | No | ND | . Echocardiography normal | ND | No | No | PTPN11 not mutated | |
|  | 51 | ND | M | 7 | ND | - | . Ptosis  . Downslanting palpebral fissures  . Low-set posteriorly rotated ears | CLS | No | ND | Echocardiography normal | ND | No | Webbed neck | PTPN11 not mutated | |
|  | 52 | ND | M | 6 | ND | - | . Ptosis  . Epicanthic folds  . Low set ears  . Low posterior hairline | CLS | No | ND | Pulmonary valvular stenosis | ND | No | No | NF1 variant (3-bp deletion in exon 25, 4312 del GAA) | |
|  | 53 | ND | M | 9 | ND | - | . Ptosis  . Malar hypoplasia | CLS, L | Developmental delay | MRI of the brain: normal | Echocardiography normal | ND | . Scoliosis  . Pectus excavatum | Keratosis pilaris | PTPN11 not mutated | |
| 2003/Castle (J Med Genet) | 54 | Familial | M | 6 | No | No | Facial dysmorphism suggesting Noonan syndrome | CLS, SNF | No | Intracranial tumours  Hydrocephalus secondary to aqueduct stenosis | No cardiac abnormality | No | No | No | NF1 variant (IVS 29+1 G-C, inactivating splice site) | |
|  | 55 | ND | M | 20 | No | No | ND | ND | Learning difficulties | Mild hydrocephalus with aqueduct stenosis | No cardiac abnormality | ND | Pectus excavatum | No | NF1 variant (microdeletion 2133delCG) | |
|  | 56 | ND | M | 10 | No | No | Facial dysmorphism | ND | No | - | Atrial septal defect | ND | No | Widely spaced nipples | NF1 variant (microinsertion 4083insTG) | |
| 2004/Yazdizadeh (Oral Surg Oral Med Oral Pathol Oral Radiol Endod) | 57 | Familial | M | 22 | ND | Yes | . Hypertelorism  . Downslanting palpebral fissures  . Broad nasal bridge  . Posteriorly angulated ears  . Large mouth  . Deep palate  . Low posterior hairline | CLS, SNF, SCNF, PNF | . Low intelligence  . Moderate mental retardation | ND | No | ND | . Scoliosis  . Kyphosis  . Pectus excavatum | . Short neck with mild webbing  . Central giant cell granuloma  . Spread nipples  . Blind (left eye) | ND | |
| 2005/Bertola (Am J Med Genet) | 58 | ND | F | 14 | No | Yes | . High forehead  . Downslanting palpebral fissures  . Posteriorly angulated ears  . Low posterior hairline | CLS, L, SNF | No | Yes  MRI of the brain: pilocystic astrocytoma | . Pulmonary valvular stenosis  . Coarctation of aorta | Yes | Pectus carinatum | . Short neck  . Umbilical hernia | PTPN11 mutated (transversion 1909A-G; Gln510Arg)  NF1 mutated (transversion, 2531A-G, Leu844Arg) | |
| 2005/De Luca (Am J Hum Genet) | 59 | Sporadic | M | 9 | Yes | No | . Ptosis  . Hypertelorism  . Low-set posteriorly rotated ears  . Thick lips  . Low posterior hairline | CLS, L, SNF | No | Yes  MRI of the brain: bilateral optic gliomas and UBOs | Echocardiography normal | No | No | Hairy naevus | NF1 c.581T-G, L194R (exon 4b, missense)  No variation of coding sequence of PTPN11 (exon 1-15 and flanking intronic stretches) | |

| **Table S1. Epidemiological characteristics, clinical manifestations and molecular diagnostics in series or case reports of neurofibromatosis-Noonan syndrome previously reported (continued)** | | | | | | | | | | | | | | | |  |
| --- | --- | --- | --- | --- | --- | --- | --- | --- | --- | --- | --- | --- | --- | --- | --- | --- |
| Year/Reference | Case number | NF1 family history | Sex | Age (y) | Macrocrania | Short stature | Facial Noonan phenotype | Skin manifestations | Neurological manifestations | RMI and/or CT-scan abnormalities | Cardiac manifestations | Lisch nodules | Osteoarticular manifestations | Other findings/manifestations | Molecular diagnosis | |
| 2005/De Luca (Am J Hum Genet) | 60 | Sporadic | F | 6.5 | Yes | No | . Ptosis  . Hypertelorism  . Downslanting palpebral fissures  . Epicanthic folds  . Flat nasal bridge  . Low-set posteriorly rotated ears  . Thick lips | CLS, L, SNF | Learning diffculties | MRI of the brain: unilateral optic gliomas and UBOs | Echocardiography normal | No | Pectus/thoracic abnormality | No | NF1 1721+3A-G (exon 11, splicing/truncating)  No variation of coding sequence of PTPN11 (exon 1-15 and flanking intronic stretches) | |
|  | 61 | Sporadic | M | 6 | No | Yes | . Hypertelorism  . Downslanting palpebral fissures  . Malar hypoplasia  . Low-set posteriorly rotated ears  . Low posterior hairline | CLS | Learning diffculties | MRI of the brain: mild thickening of optic nerves | Echocardiography normal | No | No | . Short/broad/webbed neck  . Hypotonia  . Hyperextensible joints | NF1 1756delACTA (exon 12a, deletion/truncating)  No variation of coding sequence of PTPN11 (exon 1-15 and flanking intronic stretches) | |
|  | 62 | Sporadic | F | 2.2 | Yes | No | . Ptosis  . Hypertelorism  . Downslanting palpebral fissures  . Epicanthic folds  . Low-set posteriorly rotated ears  . Thick lips  . Low posterior hairline | CLS | No | MRI of the brain: UBOs | Pulmonary valvular stenosis | No | No | Short/broad/webbed neck | NF1 1862delC (exon 12b, deletion/truncating)  No variation of coding sequence of PTPN11 (exon 1-15 and flanking intronic stretches) | |
|  | 63 | Sporadic | M | 8 | Yes | Yes | . Hypertelorism  . Downslanting palpebral fissures  . Malar hypoplasia  . Low-set posteriorly rotated ears  . High arched palate  . Macrostomia  . Large ears, malocclusion  . Low posterior hairline | CLS, L | . Mild mental retardation  . Learning difficulties | MRI of the brain normal | Echocardiography normal | No | . Scoliosis  . Pectus/thoracic abnormality  . Cubitus valgus | Short/broad/webbed neck | NF1 2153delA (exon 13, deletion/truncating)  No variation of coding sequence of PTPN11 (exon 1-15 and flanking intronic stretches) | |
|  | 64 | Familial | F | 7 | No | Yes | . Hypertelorism  . Downslanting palpebral fissures  . Epicanthic folds  . Low-set posteriorly rotated ears  . Low posterior hairline | CLS, L | Learning difficulties | MRI of the brain normal | Pulmonary valvular stenosis | ND | . Scoliosis  . Cubitus valgus | . Short/broad/webbed neck  . Myopia | NF1 2970delAAT, p.Met992del (exon 17, inframe deletion)  No variation of coding sequence of PTPN11 (exon 1-15 and flanking intronic stretches) | |
|  | 65 | Familial | M | 11 | Yes | Yes | . Hypertelorism  . Downslanting palpebral fissures  . Epicanthic folds  . Low-set posteriorly rotated ears  . Low posterior hairline | CLS, L | . Mental retardation  . Learning difficulties | MRI of the brain normal | Echocardiography normal | Yes | . Scoliosis  . Cubitus valgus | . Short/broad/webbed neck  . Cryptorchidism | NF1 2970delAAT, p.Met992del (exon 17, inframe deletion)  No variation of coding sequence of PTPN11 (exon 1-15 and flanking intronic stretches) | |

|  | 66 | Familial | F | 41 | No | Yes | . Ptosis  . Hypertelorism  . Downslanting palpebral fissures  . Malar hypoplasia  . Low-set posteriorly rotated ears  . Low posterior hairline | CLS, L, SNF | No | MRI of the brain normal | Echocardiography normal | Yes | . Scoliosis  . Pectus/thoracic abnormality  . Cubitus valgus | . Short/broad/webbed neck  . Neuroblastoma | NF1 2970delAAT, p.Met992del (exon 17, inframe deletion)  No variation of coding sequence of PTPN11 (exon 1-15 and flanking intronic stretches) |
| --- | --- | --- | --- | --- | --- | --- | --- | --- | --- | --- | --- | --- | --- | --- | --- |
|  | 67 | Familial | F | 18 | Yes | No | . Ptosis  . Hypertelorism  . Downslanting palpebral fissures  . Epicanthic folds  . Thick lips  . Low-set posteriorly rotated ears  . Low posterior hairline | CLS, L, SNF | No | MRI of the brain: UBOs, syringomyelia | Mitral valve prolapse | Yes | Scoliosis | Short/broad/webbed neck | NF1 4243G-T, pE1415* (exon 24, nonsense/truncating)  No variation of coding sequence of PTPN11 (exon 1-15 and flanking intronic stretches) |

| **Table S1. Epidemiological characteristics, clinical manifestations and molecular diagnostics in series or case reports of neurofibromatosis-Noonan syndrome previously reported (continued)** | | | | | | | | | | | | | | | |
| --- | --- | --- | --- | --- | --- | --- | --- | --- | --- | --- | --- | --- | --- | --- | --- |
| Year/Reference | Case number | NF1 family history | Sex | Age (y) | Macrocrania | Short stature | Facial Noonan phenotype | Skin manifestations | Neurological manifestations | RMI and/or CT-scan abnormalities | Cardiac manifestations | Lisch nodules | Osteoarticular manifestations | Other findings/manifestations | Molecular diagnosis |

| 2005/De Luca (Am J Hum Genet) | 68 | Familial | M | 13 | Yes | No | . Hypertelorism  . Downslanting palpebral fissures  . Low-set posteriorly rotated ears  . Low posterior hairline | CLS | Learning difficulties | MRI of the brain: UBOs | Echocardiography normal | Yes | No | No | NF1 4243G-T, pE1415* (exon 24, nonsense/truncating)  No variation of coding sequence of PTPN11 (exon 1-15 and flanking intronic stretches) |
| --- | --- | --- | --- | --- | --- | --- | --- | --- | --- | --- | --- | --- | --- | --- | --- |
|  | 69 | Familial | M | 47 | Yes | No | . Hypertelorism  . Low-set posteriorly rotated ears  . Low posterior hairline | CLS, L, SNF | No | MRI of the brain normal | Echocardiography normal | Yes | No | No | NF1 4243G-T, pE1415* (exon 24, nonsense/truncating)  No variation of coding sequence of PTPN11 (exon 1-15 and flanking intronic stretches) |
|  | 70 | Sporadic | M | 2.2 | Yes | No | . Ptosis  . Hypertelorism  . Downslanting palpebral fissures  . Epicanthic folds  . Thick lips  . Low-set posteriorly rotated ears  . Low posterior hairline | CLS | Hyperactivity | MRI of the brain normal | Pulmonary valvular stenosis | No | No | Short/broad/webbed neck | NF1 4267A-G, p.Lys1423Glu (exon 24, missense)  No variation of coding sequence of PTPN11 (exon 1-15 and flanking intronic stretches) |
|  | 71 | Sporadic | F | 6 | No | Yes | . Ptosis  . Hypertelorism  . Downslanting palpebral fissures  . Epicanthic folds  . Malar hypoplasia  . Low-set posteriorly rotated ears  . Low posterior hairline | CLS | No | MRI of the brain normal | Echocardiography normal | Yes | Pectus/thoracic abnormality | . Short/broad/webbed neck  . Eczema | NF1 4267A-G, p.Lys1423Glu (exon 24, missense)  No variation of coding sequence of PTPN11 (exon 1-15 and flanking intronic stretches) |
|  | 72 | Sporadic | F | 4 | Yes | No | . Ptosis  . Epicanthic folds, thick lips  . Low-set posteriorly rotated ears | CLS | No | MRI of the brain normal | Atrial septal defect | No | No | Sensorineural deafness | NF1 4289A-C, p.N1430T (exon 25, missense)  No variation of coding sequence of PTPN11 (exon 1-15 and flanking intronic stretches) |
|  | 73 | Sporadic | F | 40 | No | Yes | . Ptosis  . Hypertelorism  . Downslanting palpebral fissures  . Epicanthic folds  . Malar hypoplasia  . Low-set posteriorly rotated ears  . Low posterior hairline | CLS, L, SNF | Learning difficulties | MRI of the brain: optic glioma and UBOs | Echocardiography normal | Yes | . Scoliosis  . Pectus/thoracic abnormality  . Cubitus valgus | . Short/broad/webbed neck  . Rokitansky | NF1 4294G-C, p.V1432L (exon 25, missense)  No variation of coding sequence of PTPN11 (exon 1-15 and flanking intronic stretches) |
|  | 74 | Sporadic | F | 9.5 | Yes | Yes | . Ptosis  . Hypertelorism  . Downslanting palpebral fissures  . Low-set posteriorly rotated ears  . Low posterior hairline | CLS, L | No | MRI of the brain normal | Pulmonary valvular stenosis with dysplastic leaflets | No | . Scoliosis  . Pectus/thoracic abnormality  . Postaxial polydactyly of hands  . Retarded bone age | . Short/broad/webbed neck  . Keratosis pilaris  . Sensorineural deafness | NF1 4312delGAA, p.1438delE (exon 25, inframe deletion)  No variation of coding sequence of PTPN11 (exon 1-15 and flanking intronic stretches) |
|  | 75 | Sporadic | F | 38 | No | Yes | . Downslanting palpebral fissures  . Epicanthic folds  . Low-set posteriorly rotated ears  . High arched palate  . Low posterior hairline | CLS, L, SNF, PNF | No | MRI of the brain normal | Mitral valve prolapse | ND | Pectus/thoracic abnormality | Short/broad/webbed neck | NF1 5339T-G, p.L1780* (exon 29, nonsense/truncating) |
|  | 76 | Sporadic | M | 10 | Yes | No | . Ptosis  . Thick lips  . Low-set posteriorly rotated ears | CLS, L | No | MRI of the brain: UBOs | Arrythmia (right-branch block) | Yes | Pectus/thoracic abnormality | Renal cyst | NF1 6641 +1G-A (exon 35, splicing/truncating)  No variation of coding sequence of PTPN11 (exon 1-15 and flanking intronic stretches) |

| **Table S1. Epidemiological characteristics, clinical manifestations and molecular diagnostics in series or case reports of neurofibromatosis-Noonan syndrome previously reported (continued)** | | | | | | | | | | | | | | | |  |
| --- | --- | --- | --- | --- | --- | --- | --- | --- | --- | --- | --- | --- | --- | --- | --- | --- |
| Year/Reference | Case number | NF1 family history | Sex | Age (y) | Macrocrania | Short stature | Facial Noonan phenotype | Skin manifestations | Neurological manifestations | RMI and/or CT-scan abnormalities | Cardiac manifestations | Lisch nodules | Osteoarticular manifestations | Other findings/manifestations | Molecular diagnosis | |

| 2005/De Luca (Am J Hum Genet) | 77 | Familial | M | 17.7 | Yes | No | . Ptosis  . Hypertelorism  . Downslanting palpebral fissures  . Epicanthic folds  . Low-set posteriorly rotated ears  . Thick lips  . Low posterior hairline | CLS, L, SNF | . Mental retardation  . Learning difficulties  . Seizures | MRI of the brain normal | Echocardiography normal | No | . Scoliosis  . Pectus/thoracic abnormality | . Short/broad/webbed neck  . Bilateral conductive deafness | NF1 7877delG (exon 45, deletion/truncating)  No variation of coding sequence of PTPN11 (exon 1-15 and flanking intronic stretches) |
| --- | --- | --- | --- | --- | --- | --- | --- | --- | --- | --- | --- | --- | --- | --- | --- |
|  | 78 | Familial | M | 45 (same family 17.7) | Yes | No | . Ptosis  . Hypertelorism  . Downslanting palpebral fissures  . Thick lips  . Low-set posteriorly rotated ears  . Low posterior hairline | CLS, L, SNF | Learning difficulties | MRI of the brain normal | Echocardiography normal | Yes | Scoliosis | No | NF1 7877delG (exon 45, deletion/truncating)  No variation of coding sequence of PTPN11 (exon 1-15 and flanking intronic stretches) |
|  | 79 | Sporadic | M | 7 | No | No | . Ptosis  . Hypertelorism  . Downslanting palpebral fissures  . Sparse lateral eyebrows  . Low-set posteriorly rotated ears | CLS, L | . Mental retardation  . Learning difficulties | MRI of the brain: optic glioma and UBOs | Echocardiography normal | Yes | . Pectus/thoracic abnormality  . Cubitus valgus | No | NF1 partial deletion (< 1.7 Mb, between D17S1849 and D17S798)  No variation of coding sequence of PTPN11 (exon 1-15 and flanking intronic stretches) |
|  | 80 | Sporadic | M | 12 | No | Yes | . Ptosis  . Hypertelorism  . Downslanting palpebral fissures  . Epicanthic folds  . Low-set posteriorly rotated ears  . Low posterior hairline | CLS, L | Learning difficulties | MRI of the brain: UBOs | Echocardiography normal | No | Pectus/thoracic abnormality  Cubitus valgus | Short/broad/webbed neck | Five exonic and intragenic polymorphisms of NF1  No variation of coding sequence of PTPN11 (exon 1-15 and flanking intronic stretches) |
| 2006/Stevenson (Clin Genet) and 2007/Upadhyaya (Am J Hum Genet) | 81 | Familial | F | ND | Yes | No | . Ptosis  . Malar hypoplasia  . Posteriorly angulated ears | CLS, L | No | - | No | ND | No | Slightly broad neck | NF1 variant 2971delAAT, p.Met992del  PTPN11 haplotype analysis C12 (no segregation with Noonan features) |
|  | 82 | Familial | F | 14 | Yes | Yes | . Ptosis (mild right)  . Frontal upsweep to the hair, low nasal root,  . Down-slanting palpebral fissures  . Posteriorly rotated ears (slightly)  . Malar hypoplasia | CLS | No | MRI of the brain: normal | No | ND | Pectus (mild, chest) carinatum superiorly and excavatum inferiorly | . Broad neck (mild) with slight webbing contour  . Telecanthus | NF1 variant 2971delAAT, p.Met992del  PTPN11 haplotype analysis C12 (no segregation with Noonan features) |
|  | 83 | Familial | M | 13 | Yes | Yes | . Ptosis (bilateral)  . Frontal upsweep to the hair  . Down-slanting palpebral fissures  . Low nasal root | CLS, L | Speech therapy for articulation defects | MRI of the brain: normal | Pulmonary valvular stenosis | ND | Pectus excavatum | . Mild webbing contour to his neck  . Telecanthus | NF1 variant 2971delAAT, p.Met992del  PTPN11 haplotype analysis C12 (no segregation with Noonan features) |
|  | 84 | Familial | M | 10 | Yes | No | . Ptosis (mild)  . Low nasal root | CLS | Mild developmental delay (speech therapy) | ND | No | ND | Pectus excavatum | No | NF1 variant 2971delAAT, p.Met992del  PTPN11 haplotype analysis C12 (no segregation with Noonan features) |
|  | 85 | Familial | F | 8 | Yes | Yes | . Triangular face  . Ptosis (severe)  . Epicanthal folds  . Malar hypoplasia  . Posteriorly rotated ears (small and over-folded)  . Low nasal root | CLS | Mild developmental delay (speech delay) | ND | . Pulmonary valvular and supravalvular stenosis  . Atrial septal defect | ND | Pectus (mild) carinatum superiorly and excavatum inferiorly | . Neck webbing  . Telecanthus  . Sloped shoulders | NF1 variant 2971delAAT, p.Met992del  PTPN11 haplotype analysis C12 (no segregation with Noonan features) |

| **Table S1. Epidemiological characteristics, clinical manifestations and molecular diagnostics in series or case reports of neurofibromatosis-Noonan syndrome previously reported (continued)** | | | | | | | | | | | | | | | |  |
| --- | --- | --- | --- | --- | --- | --- | --- | --- | --- | --- | --- | --- | --- | --- | --- | --- |
| Year/Reference | Case number | NF1 family history | Sex | Age (y) | Macrocrania | Short stature | Facial Noonan phenotype | Skin manifestations | Neurological manifestations | RMI and/or CT-scan abnormalities | Cardiac manifestations | Lisch nodules | Osteoarticular manifestations | Other findings/manifestations | Molecular diagnosis | |

| 2006/Hüffmeier (Am J Med Genet) | 86 | Sporadic | M | 4 | No | No | . High broad forehead  . Ptosis  . Hypertelorism (subjective)  . Downslanting palpebral fissures  . Eyebrows medial thinning  . Broad nasal bridge  . Low set posteriorly rotated ears  . Low posterior hair line  . Frontal cowlick/sturdy hair | CLS (5) | Development remarkably delayed: spoke only few single words at the age of 4 years, slightly delayed motor skills  Restless, “self-aggressive”, temper tantrums | ND | . Pulmonary valvular stenosis (resolved spontaneously by 4 years of life) | No | . Broad chest  . Pectus excavatum inferior  . Pectus carinatum superior | . Webbed neck  . Merckel diverticula  . Inverted nipples | NF1 variant L1196R(c.3587T>G, exon 21)  PTPN11 normal |
| --- | --- | --- | --- | --- | --- | --- | --- | --- | --- | --- | --- | --- | --- | --- | --- |
|  | 87 | Familial | M | 20 | No | Yes | . Ptosis  . Hypertelorism (subjective)  . High broad forehead  . Downslanting palpebral fissures  . Broad nasal bridge  . Low set posteriorly rotated ears  . Low posterior hair line | CLS, SNF | Low normal development | ND | No | No | . Scoliosis  . Broad chest  . Pectus excavatum inferior  . Pectus carinatum superior | . Webbed neck  . Corneal irregularity  . Ametropia  . Umbilical hernia  . Urethral valves | NF1 variant c.1756_1759del4, exon 21)  PTPN11 normal |
|  | 88 | Familial | M | 0.6 | No | No | . Hypertelorism (subjective)  . High broad forehead  . Downslanting palpebral fissures  . Broad nasal bridge  . Eyebrows medial thinning  . Low set posteriorly rotated ears  . Low posterior hair line | CLS | No | ND | Aortic insufficiency | No | . Pectus excavatum inferior  . Pectus carinatum superior | . Webbed neck  . Astigmatism | NF1 variant c.1756_1759del4, exon 21)  PTPN11 not performed |
|  | 89 | Familial | F | 37 | ND | No | . Ptosis  . Hypertelorism  . High broad forehead  . Downslanting palpebral fissures  . Broad nasal bridge  . Low posterior hair line | CLS, L, SNF | Normal development | ND | No | Yes | . Broad chest  . Pectus excavatum inferior | . Webbed neck | NF1 variant c.3060delA (exon 18)  PTPN11 not performed |
|  | 90 | Familial | F | 6 | Yes | No | . Ptosis  . Hypertelorism (subjective)  . High broad forehead  . Downslanting palpebral fissures  . Broad nasal bridge  . Eyebrows medial thinning  . Low posterior hair line | CLS, L, SNF | Normal development | No | No | Yes | . Scoliosis  . Broad chest  . Pectus excavatum inferior  . Pectus carinatum superior | . Webbed neck  . Enlarged tongue  . Large epidermal nevus (anterior neck) | NF1 variant c.3060delA (exon 18)  PTPN11 normal |
|  | 91 | Familial | M | 42 | Yes | Yes | . Ptosis  . Hypertelorism  . High broad forehead  . Downslanting palpebral fissures  . Broad nasal bridge  . Posteriorly rotated ears  . Low posterior hair line | CLS, L, SNF | Normal development | No | No | Yes | . Scoliosis  . Pectus excavatum inferior  . Pectus carinatum superior | Webbed neck | NF1 variant c.796_803del8 (exon 6)  PTPN11 normal |
|  | 92 | Sporadic | F | 20 | Yes | Yes | . Hypertelorism  . High broad forehead  . Downslanting palpebral fissures  . Eyebrows medial thinning  . Broad nasal bridge  . Low set posteriorly rotated ears  . Low posterior hair line | CLS, L, SNF | Intelligence below familiar background | No | No | Yes | . Scoliosis  . Broad chest  . Pectus excavatum inferior | . Webbed neck  . Hyperopia | NF1 whole gene deletion  PTPN11 normal |

| **Table S1. Epidemiological characteristics, clinical manifestations and molecular diagnostics in series or case reports of neurofibromatosis-Noonan syndrome previously reported (continued)** | | | | | | | | | | | | | | | |  |
| --- | --- | --- | --- | --- | --- | --- | --- | --- | --- | --- | --- | --- | --- | --- | --- | --- |
| Year/Reference | Case number | NF1 family history | Sex | Age (y) | Macrocrania | Short stature | Facial Noonan phenotype | Skin manifestations | Neurological manifestations | RMI and/or CT-scan abnormalities | Cardiac manifestations | Lisch nodules | Osteoarticular manifestations | Other findings/manifestations | Molecular diagnosis | |

| 2009/Nyström (Acta Paediatr) | 93 | Familial | F | 21 | ND | Yes | . Ptosis (bilateral)  . Hypertelorism  . Downslanting palpebral fissures  . Epicanthal folds  . Low set ears | CLS | . Increased reflexes  . Ataxic gait  . Headaches  . Signs of hydrocephalus (ventriculo-peritoneal shunt)  . Spastic paraparesis  . Mild mental retardation | MRI brain/spinal: syringomyelia, Arnold-Chiari I malformation, corpus callosum hypoplasia of, reduction brain’s white matter | . Pulmonary valvular stenosis  . Atrial septal defect | ND | . Scoliosis (regarded as secondary to Arnold-Chiari I)  . Shield chest  . Pectus/thoracic abnormalities | . Short/broad/webbed neck. Tendency of bleeding (thrombocytopenia)  . Premature menopause (15 y)  . Optic atrophy (right, hydrocephalus)  Anomalous disc  . High myopia, deep amblyopia and strabismus (left) | NF1 variant c.5425C>T, p.Arg1809Cyst (exon 38)  PTPN11 variant c.853T>C, p.F285L (exon 7) |
| --- | --- | --- | --- | --- | --- | --- | --- | --- | --- | --- | --- | --- | --- | --- | --- |
| 2009/Nyström (Clin Genet) | 94 | Familial | F | 63 | Yes | No | . Hypertelorism  . Downslanting palpebral fissures  . Low set ears  . Low posterior hairline | CLS | No | No | No | Yes | . Broad chest  . Cubitus valgus | . Short and webbed neck/pterygium colli  . Multiple naevi  . Growing mandible | NF1 variant c.4128C>T, p.L1390F (exon 24)  PTPN11, BRAF, RAF1, KRAS, MEK1, MEK2 and SPRED1 normal |
|  | 95 | Familial | F | 44 | Yes | No | . Hypertelorism  . Downslanting palpebral fissures  . Low set ears  . Low posterior hairline | CLS | No | No | . Pulmonary valvular stenosis  . Hypertrophic cardiomyopathy | ND | Broad chest | . Short, webbed neck/pterygium colli  . Multiple naevi  . Multiple lentigines  . Small feet  . Talipes cavus | NF1 variant c.4128C>T, p.L1390F (exon 24)  PTPN11, BRAF, RAF1, KRAS, MEK1, MEK2 and SPRED1 normal |
|  | 96 | Familial | F | 42 | No | Yes | . Hypertelorism  . Low posterior hairline | CLS, L | No | No | Pulmonary valvular stenosis | Yes | . Broad chest  . Cubitus valgus | . Short neck  . Small feet  . Hypermobility of jaws, thumbs, knees  . Colon irritable | NF1 variant c.4128C>T, p.L1390F (exon 24)  PTPN11, BRAF, RAF1, KRAS, MEK1, MEK2 and SPRED1 normal |
|  | 97 | Familial | F | 24 | No | No | Low set ears | CLS | No | No | Ventricular septal defect | ND | No | . Webbed neck/pterygium colli | NF1 variant c.4128C>T, p.L1390F (exon 24)  PTPN11, BRAF, RAF1, KRAS, MEK1, MEK2 and SPRED1 normal |
|  | 98 | Familial | F | 21 | Yes | No | . Hypertelorism  . Downslanting palpebral fissures  . Low set ears  . Low posterior hairline | CLS (3), L | No | No | No | Yes | No | Webbed neck/pterygium colli | NF1 variant c.4128C>T, p.L1390F (exon 24)  PTPN11, BRAF, RAF1, KRAS, MEK1, MEK2 and SPRED1 normal |
|  | 99 | Familial | M | 19 | Yes | No | . Hypertelorism  . Downslanting palpebral fissures,  . Low set ears  . Low posterior hairline | CLS, L | Muscular hypotonia | No | Pulmonary valvular stenosis | ND | Broad chest | . Short neck  . Webbed neck/pterygium colli  . Undescended testes  . Multiple naevi | NF1 variant c.4128C>T, p.L1390F (exon 24)  PTPN11, BRAF, RAF1, KRAS, MEK1, MEK2 and SPRED1 normal |
|  | 100 | Familial | M | 1.75 | Yes | Yes | . Hypertelorism  . Ptosis  . Downslanting palpebral fissures  . Low set ears  . Low posterior hairline | CLS (2-3) | . Seizures  . Motor delay  . Retarded speech development | No | No | ND | Broad chest | . Short and webbed neck/pterygium colli | NF1 variant c.4128C>T, p.L1390F (exon 24)  PTPN11, BRAF, RAF1, KRAS, MEK1, MEK2 and SPRED1 normal |
| 2009/Thiel (Am J Med Genet) | 101 | Familial | F | 1.5 | No | Yes | . Hypertelorism  . Broad and flat nasal bridge  . Small mouth  . Low set ears  . Low posterior hairline | CLS | Delayed psychometric development | MRI of brain: bilateral optic nerve gliomas | Pulmonary valvular stenosis | ND | . Mild funnel chest  . Clinodactyly (V)  . Tapering fingers  . Wide internipple distance  . Optic atrophy of the fundus | Short neck | NF1 variant c.4661+1G>C (splice site variant, exon 27)  PTPN11 variant c.5C>T (p.Thr2Ile) |
|  | 102 | Familial | F | 32 | No | Yes | Downslanting palpebral fissures | CLS, L | No | No | No | ND | No | . Mild blepharoptosis  . Mild myopia | NF1 variant c.4661+1G>C (exon 27)  PTPN11 normal |

| **Table S1. Epidemiological characteristics, clinical manifestations and molecular diagnostics in series or case reports of neurofibromatosis-Noonan syndrome previously reported (continued)** | | | | | | | | | | | | | | | |  |
| --- | --- | --- | --- | --- | --- | --- | --- | --- | --- | --- | --- | --- | --- | --- | --- | --- |
| Year/Reference | Case number | NF1 family history | Sex | Age (y) | Macrocrania | Short stature | Facial Noonan phenotype | Skin manifestations | Neurological manifestations | RMI and/or CT-scan abnormalities | Cardiac manifestations | Lisch nodules | Osteoarticular manifestations | Other findings/manifestations | Molecular diagnosis | |

| 2011/Prada Am J Med Genet | 103 | Familial | M | 0.1 | No | No | . Hypertelorism  . Epicanthic folds (bilateral)  . Broad nasal nasal bridge  . Broad nasal tip  . Anteverted nares  . Short philtrum  . Low set posteriorly rotated ears | CLS, SNF (?), PNF (?) | No | No optic glioma | Hypertrophic cardiomyopathy | ND | Pectus carinatum | . Single palmar crease  . Thick and short fingers  . Cryptorchidism  . Macropenis  . Congenital hypothyroidism | NF1 variant c.922A>G, p.Asn1430Asp  PTPN11 variant c.922A>G, p.Asn308Asp |
| --- | --- | --- | --- | --- | --- | --- | --- | --- | --- | --- | --- | --- | --- | --- | --- |
| 2011/Reig (Dermatol Online J) | 104 | Sporadic | M | 17 | Yes | Yes | . Triangular face  . Low-set posteriorly rotated ears | CLS, L, SNF | Delayed psychomotor development and language (slight) | No | . Hypertrophic cardiomyopathy  . Pulmonary stenosis  . Septal ventricular defect | ND | Pectus carinatum | . Short neck  . Growth hormone deficiency  . Strabismus | NF1 variant c.7192-7193delCT |
| 2012/Yimenicioglu (Childs Nerv Syst) | 105 | Sporadic | M | 14 | No | Yes | . Triangular face  . Ptosis (left)  . Prominent forehead  . Small ears  . Deep set eyes  . Downslanting palpebral fissures  . Maxillary hypoplasia  . High nasal bridge  . Short philtrum | CLS, SNF | Articulation problems | MRI: ventricular hydrocephaly | Pulmonary valvular stenosis | Yes | . Scoliosis (mild, thoracic)  . Pectus carinatum  . Long, hyperextensible fingers  . Lobulated and septated cyst of femoral bone | . Simian crease on the left hand  . Webbed neck | NF1 variant c.7549C>T (p.R2517X, exon 51)  PTPN11, RAF1 (exons 7,12,14 and 17), KRAS Isoform B (exons 2, 3, and 5), SOS1 (exons 3-11, 13-14, and 16), and SHOC2 (exon 2) normal |
| 2012/Croonen (Clin Dysmorphol) | 106 | Sporadic | M | 5 | No | Yes | . Hypertelorism  . Ptosis  . Epicanthus  . Downslanting palpebral fissures  . Small right orbit  . Low-set ears | CLS | Language and speech delay development | MRI of brain normal | No | ND | Pectus excavatum | . Webbed neck  . Wide internipple distance  . Cryptochidism  . Ureterocele | NF1 c.3586C>T, p.Leu1196Phe  PTPN11, RAF1, KRAS, SOS1, BRAF, MAP2K1, NRAS, SHOC2, CBL: no variant |
| 2013/Ben-Shachar (Eur J Hum Genet) | 107 | Familial | ND | ND | ND | ND | ND | ND | No | ND | No | ND | ND | ND | NF1 c.2970del AAT (in frame) |
|  | 108 | Sporadic | ND | ND | ND | ND | ND | ND | No | ND | No | ND | ND | ND | NF1 exon 14 deletion (in-frame) |
|  | 109 | Familial | ND | ND | ND | ND | ND | ND | No | ND | No | ND | ND | ND | NF1 7702C>T, p.Q2568X (non-sense) |
|  | 110 | Sporadic | ND | ND | ND | ND | ND | ND | No | ND | No | ND | ND | ND | NF1 c.7324del CTT (in-frame) |
| 2014/Ekvall (Am J Med Genet A) | 111 | Familial | F | 69 | Yes | No | ND | CLS, L | School problems | ND | No | ND | Thoracic deformity | No | NF1 variant c.5425C>T, p.Arg1809Cyst (exon 38)  PTPN11, SOS1, BRAF, RAF1, KRAS, MEK1, MEK2, NRAS, CBL (exons 7-9), SHOC2 (exon2) and SPRED1 normal |
|  | 112 | Familial | M | 43 | Yes | No | . Hypertelorism (slight)  . Ptosis  . Downslanting palpebral fissures  . Low posterior hairline | CLS, L | . School problems  . IQ:70 | ND | No | Yes | No | . Short neck  . Multiple naevi | NF1 variant c.5425C>T, p.Arg1809Cyst (exon 38)  PTPN11, SOS1, BRAF, RAF1, KRAS, MEK1, MEK2, NRAS, CBL (exons 7-9), SHOC2 (exon2) and SPRED1 normal |
|  | 113 | Familial | M | 9 | Yes | Yes | Low posterior hairline | CLS | . Motor delay  . Retarded speech development  . School problems  . IQ:76 | ND | No | Yes | No | . Short broad neck  . Refraction error | NF1 variant c.5425C>T, p.Arg1809Cyst (exon 38)  PTPN11, SOS1, BRAF, RAF1, KRAS, MEK1, MEK2, NRAS, CBL (exons 7-9), SHOC2 (exon2) and SPRED1 normal |
|  | 114 | Familial | M | 42 | Yes | No | ND | CLS, L, SNF | No | ND | No | Yes | No | . Lipoma  . Fibroma | NF1 variant c.6789_6792delITTAC (p.Y2264Tfs*6, exon 37)  SPRED1 normal |

| **Table S1. Epidemiological characteristics, clinical manifestations and molecular diagnostics in series or case reports of neurofibromatosis-Noonan syndrome previously reported (continued)** | | | | | | | | | | | | | | | |  |
| --- | --- | --- | --- | --- | --- | --- | --- | --- | --- | --- | --- | --- | --- | --- | --- | --- |
| Year/Reference | Case number | NF1 family history | Sex | Age (y) | Macrocrania | Short stature | Facial Noonan phenotype | Skin manifestations | Neurological manifestations | RMI and/or CT-scan abnormalities | Cardiac manifestations | Lisch nodules | Osteoarticular manifestations | Other findings/manifestations | Molecular diagnosis | |

| 2014/Ekvall (Am J Med Genet A) | 115 | Familial | F | 2 | Yes | Yes | . Hypertelorism  . Ptosis (slight, bilateral)  . Bitemporal narrowing  . Downslanting palpebral fissures  . Low set ears  . Thick helices  . Thin lips with downturned corners  . Low posterior hairline | CLS | No | ND | . Pulmonary valvular stenosis  . Ventricular septal defect | Yes | No | . Short broad neck  . Diabetes type I | NF1 variant c.6789_6792delITTAC (p.Y2264Tfs*6, exon 37)  PTPN11, SOS1, BRAF, RAF1, KRAS, MEK1, MEK2, NRAS, CBL (exons 7-9), SHOC2 (exon2) and SPRED1 normal |
| --- | --- | --- | --- | --- | --- | --- | --- | --- | --- | --- | --- | --- | --- | --- | --- |
|  | 116 | Familial | M | 1 | No | No | . Hypertelorism  . Downslanting palpebral fissures | CLS | No | ND | No | ND | No | No | NF1 variant c.6789_6792delITTAC (p.Y2264Tfs*6, exon 37) |
|  | 117 | Familial | F | 11 | No | Yes | . Ptosis  . Downslanting palpebral fissures  . Low set ears  . Low posterior hairline | CLS, SNF | Motor delay | MRI of the brain: T2-weighted images (UBO) | No | Yes | . Broad chest  . Delayed skeletal development | . Short neck  . Hamartoma  . Partial growth hormone deficiency  . Hyperopia  . Strabismus | NF1 variant c.2991-1G>A (intron 17)  PTPN11, SOS1, BRAF, RAF1, KRAS, MEK1, MEK2, NRAS, CBL (exons 7-9), SHOC2 (exon2) and SPRED1 normal |
| 2015/Pinna (Eur J Hum Genet) | 118 | Sporadic | M | 16 | Yes | No | . Triangular face  . Ptosis  . Low set posteriorly rotated ears  . Close palate | CLS, L | Learning problems | No optic glioma | No pulmonary stenosis | No | . Scoliosis  . Pectus excavatum  . Scapular winging, | . Mild joint laxity  . Skin hyperelasticity, easy  . Extensibility of ears  . Flat feet  . Celiac disease | NF1 variant c.5425C>T, p.Arg1809Cyst (exon 38) |
|  | 119 | Sporadic | F | 10 | No | No | . Hypertelorism  . Low set posteriorly rotated ears | CLS, L | No | No optic glioma | No pulmonary stenosis | No | Pectus excavatum | Short neck | NF1 variant c.5425C>T, p.Arg1809Cyst (exon 38) |
|  | 120 | Familial | F | 35 | No | Yes | . Ptosis  . Low set posteriorly rotated ears | CLS, L | No | ND | No pulmonary stenosis | ND | No | No | NF1 variant c.5425C>T, p.Arg1809Cyst (exon 38) |
|  | 121 | Familial | M | 6 | No | No | . Hypertelorism  . Low set posteriorly rotated ears | CLS, L | No | ND | No pulmonary stenosis | No | No | No | NF1 variant c.5425C>T, p.Arg1809Cyst (exon 38) |
| 2015/Santoro (Eur J Hum Genet) | 122 | Familial | M | 41 | No | No | Low set posteriorly rotated ears | CLS, L | No | ND | No pulmonary stenosis | No | No | . Short neck  . Lipomas  . Wide-set nipples | NF1 variant c.5425C>T, p.Arg1809Cyst (exon 38) |
|  | 123 | Familial | F | 40 | Yes | No | . Hypertelorism  . Low set posteriorly rotated ears  . Bulbous nasal tip | CLS, L | Learning difficulties | ND | No pulmonary stenosis | No | No | . Lipomas  . Ectopic kidney | NF1 variant c.5426G>T, p.Arg1809Leu (exon 38) |
|  | 124 | Familial | F | 3 | Yes | No | . Hypertelorism  . Bulbous nasal tip | CLS | No | ND | No pulmonary stenosis | No | No | No | NF1 variant c.5426G>T, p.Arg1809Leu (exon 38) |
| 2015/Rojnueangnit (Hum Mutat) | 125 | Familial | F | 0-1 | No | Yes | . Ptosis  . Downslanting fissures | CLS | Mild development delay | ND | No | No | No | No | NF1 variant c.5426G>T, p.Arg1809Leu (exon 38)  PTPN11 testing negative |
|  | 126 | Familial | M | 2-4 | No | No | . Hypertelorism  . Mid-facial hypoplasia  . Low-set ears | CLS | . Speech delay  . Learning disability | No optic glioma (MRI) | No | Yes | . Scoliosis  . Pectus carinatum | No | NF1 variant c.5426G>T, p.Arg1809Cys (exon 38)  PTPN11 testing negative |
|  | 127 | Familial | F | 5-8 | No | No | . Mid-facial hypoplasia  . Low-set ears | CLS, L | Learning disability | ND | No | No | No | No | NF1 variant c.5426G>T, p.Arg1809Cys (exon 38)  PTPN11 testing negative |
|  | 128 | Familial | F | 9-13 | Yes | Yes | . Mid-facial hypoplasia  . Low-set ears | CLS | No | No optic glioma (MRI) | No | No | No | No | NF1 variant c.5426G>T, p.Arg1809Cys (exon 38)  PTPN11 testing negative |
|  | 129 | Familial | F | 5-8 | No | Yes | . Hypertelorism  . Mid-facial hypoplasia | CLS, L | No | No optic glioma (MRI) | . Pulmonary stenosis  . Atrium septal defect | No | No | Venous malformation | NF1 variant c.5426G>T, p.Arg1809Leu (exon 38)  PTPN11 testing negative |

| **Table S1. Epidemiological characteristics, clinical manifestations and molecular diagnostics in series or case reports of neurofibromatosis-Noonan syndrome previously reported (continued)** | | | | | | | | | | | | | | | |  |
| --- | --- | --- | --- | --- | --- | --- | --- | --- | --- | --- | --- | --- | --- | --- | --- | --- |
| Year/Reference | Case number | NF1 family history | Sex | Age (y) | Macrocrania | Short stature | Facial Noonan phenotype | Skin manifestations | Neurological manifestations | RMI and/or CT-scan abnormalities | Cardiac manifestations | Lisch nodules | Osteoarticular manifestations | Other findings/manifestations | Molecular diagnosis | |

| 2015/Rojnueangnit (Hum Mutat) | 130 | Familial | M | 19-26 | No | Yes | . Hypertelorism  . Mid-facial hypoplasia | CLS, L | No | ND | No | No | No | . Webbed neck  . Becker’s nevus | NF1 variant c.5426G>T, p.Arg1809Leu (exon 38)  PTPN11 testing negative |
| --- | --- | --- | --- | --- | --- | --- | --- | --- | --- | --- | --- | --- | --- | --- | --- |
|  | 131 | Familial | M | 14-18 | No | Yes | . Hypertelorism  . Low set ears | CLS, L | Learning disability | ND | No | ND | No | No | NF1 variant c.5426G>T, p.Arg1809Cys (exon 38) |
|  | 132 | Familial | F | 36-50 | No | No | . Hypertelorism  . Low set ears | CLS, L | Learning disability | ND | No | ND | No | No | NF1 variant c.5426G>T, p.Arg1809Cys (exon 38)  PTPN11 testing negative |
|  | 133 | Familial | F | 9-13 | No | Yes | . Hypertelorism  . Low set ears | CLS, L | Learning disability | No optic glioma (MRI) | No | ND | No | No | NF1 variant c.5426G>T, p.Arg1809Cys (exon 38) |
|  | 134 | Familial | F | 2-4 | No | No | . Hypertelorism  . Low set ears  . Mid-facial hypoplasia | CLS | No | ND | Pulmonary stenosis | No | No | . Webbed neck  . Twenty nail dystrophy | NF1 variant c.5426G>T, p.Arg1809Cys (exon 38)  PTPN11, SOS1, RAF1 testing negative |
|  | 135 | Familial | F | 36-50 | ND | ND | . Hypertelorism  . Low set ears | CLS, L | No | ND | No | ND | No | . Webbed neck | NF1 variant c.5426G>T, p.Arg1809Cys (exon 38) |
|  | 136 | Familial | F | 9-13 | No | Yes | Hypertelorism | CLS, L | No | No optic glioma (MRI) | No | No | No | . Webbed neck  . Naevus anemicus | NF1 variant c.5426G>T, p.Arg1809Gly (exon 38) |
|  | 137 | Familial | F | 36-50 | ND | Yes | Hypertelorism | CLS, L | No | No optic glioma (MRI) | No | No | No | . Webbed neck  . Fibroadenoma breast | NF1 variant c.5426G>T, p.Arg1809Gly (exon 38) |
|  | 138 | Familial | M | 36-50 | ND | No | . Hypertelorism  . Low set ears | CLS (<6), L | No | No optic glioma (MRI) | No | No | No | . Webbed neck  . Diabetes | NF1 variant c.5426G>T, p.Arg1809Gly (exon 38) |
|  | 139 | Familial | M | 14-18 | No | Yes | . Hypertelorism  . Down-slanting palpebral fissures  . Epicanthus folds | CLS, L | . Learning disability  . DFSIQ = 88 | No optic glioma (MRI) | No | No | No | No | NF1 variant c.5426G>T, p.Arg1809Cys (exon 38) |
|  | 140 | Familial | F | 14-18 | No | Yes | Hypertelorism | CLS, L | . Learning disability,  . DFSIQ = 67 | . Arnold Chiari type 1  . Meningocele (T10-T11)  . G. epilepsy | No | ND | No | No | NF1 variant c.5426G>T, p.Arg1809Cys (exon 38) |
|  | 141 | Familial | F | 27-35 | ND | No | ND | CLS, L | No | ND | . Pulmonary stenosis  . Pulmonary artery ectasia | No | No | Webbed neck | NF1 variant c.5426G>T, p.Arg1809Cys (exon 38) |
|  | 142 | Familial | M | 0-2 | No | Yes | . Ptosis  . Mid-facial hypoplasia  . Low set ears | CLS | Speech delay | ND | No | No | No | Dysgenesis extraocular muscles | NF1 variant c.5426G>T, p.Arg1809Cys (exon 38) |
|  | 143 | Familial | M | 0-2 | No | Yes | . Hypertelorism  . Ptosis  . Low set ears | CLS | Speech delay | ND | No | No | No | . Webbed neck  . Dysgenesis extraocular muscles | NF1 variant c.5426G>T, p.Arg1809Cys (exon 38) |
|  | 144 | Familial | F | 5-8 | No | Yes | . Hypertelorism  . Ptosis  . Epicanthus folds | CLS, L | . Learning disability  . IQ = 58  . Tics  . Attention deficit hyperactivity disorder | ND | Pulmonary stenosis (valvular and supravalvular) | No | Pectus excavatum | No | NF1 variant c.5426G>T, p.Arg1809Ser (exon 38) de novo  Father (NF1 splice mutation c.6365-2A>G) |
|  | 145 | Sporadic | M | 5-8 | ND | ND | Low-set ears | CLS | . Learning disability  . Abnormal development | No optic glioma (MRI) | Pulmonary stenosis | No | No | No | NF1 variant c.5426G>T, p.Arg1809Cys (exon 38)  PTPN11 testing negative |

| **Table S1. Epidemiological characteristics, clinical manifestations and molecular diagnostics in series or case reports of neurofibromatosis-Noonan syndrome previously reported (continued)** | | | | | | | | | | | | | | | |  |
| --- | --- | --- | --- | --- | --- | --- | --- | --- | --- | --- | --- | --- | --- | --- | --- | --- |
| Year/Reference | Case number | NF1 family history | Sex | Age (y) | Macrocrania | Short stature | Facial Noonan phenotype | Skin manifestations | Neurological manifestations | RMI and/or CT-scan abnormalities | Cardiac manifestations | Lisch nodules | Osteoarticular manifestations | Other findings/manifestations | Molecular diagnosis | |

| 2015/Rojnueangnit (Hum Mutat) | 146 | Sporadic | F | 9-13 | Yes | No | ND | CLS, L | . Learning disability  . Speech delay | ND | Pulmonary stenosis | ND | 2^nd^ and 5^th^ short toes bilaterally | Short neck | NF1 variant c.5426G>T, p.Arg1809Cys (exon 38)  PTPN11 testing negative |
| --- | --- | --- | --- | --- | --- | --- | --- | --- | --- | --- | --- | --- | --- | --- | --- |
|  | 147 | Sporadic | M | 5-8 | ND | Yes | ND | CLS, L | Learning disability | ND | Pulmonary stenosis | ND | No | Short neck | NF1 variant c.5426G>T, p.Arg1809Cys (exon 38)  PTPN11 testing negative |
|  | 148 | Sporadic | M | 14-18 | No | No | . Hypertelorism  . Downslanted palpebral fissures | CLS, L | . Motor delay  . Learning disability  . Asperger  . Anxiety | No optic glioma (MRI) | No | Yes (1) | . Scoliosis  . Pectus excavatum | . Prominent pituitary  . Iris freckles | NF1 variant c.5426G>T, p.Arg1809Cys (exon 38) |
|  | 149 | Sporadic | F | 9-13 | No | Yes | . Hypertelorism  . Midface hypoplasia  . Low-set ears | CLS, L | . Learning disability  . Speech delay  . Attention deficit hyperactivity disorder  . Velopharyngeal insufficiency  . Nasal speech  . Speech apraxia | No optic glioma (MRI) | Echocardiography normal | ND | Pectus excavatum | . Short neck  . Webbed neck  . Arterial hypertension (essential) | NF1 variant c.5426G>T, p.Arg1809Cys (exon 38) |
|  | 150 | Sporadic | M | 14-18 | No | Yes | ND | CLS | Learning disability | ND | No | No | Pectus excavatum | Webbed neck | NF1 variant c.5426G>T, p.Arg1809Cys (exon 38) |
|  | 151 | Sporadic | F | 9-13 | ND | ND | . Hypertelorism  . Ptosis  . Posteriorly rotated ears  . Low posterior hairline  . Bright blue eyes | CLS | Learning disability | ND | No | ND | Scoliosis | No | NF1 variant c.5426G>T, p.Arg1809Cys (exon 38)  PTPN11 testing negative |
|  | 152 | Sporadic | M | 5-8 | ND | ND | Hypertelorism | CLS, L | No | ND | Pulmonary stenosis | ND | No | No | NF1 variant c.5426G>T, p.Arg1809Cys (exon 38)  PTPN11 testing negative |
|  | 153 | ND | F | 0-2 | No | No | Low set ears | CLS | . Developmental delay  . Gross and fine motor delays | ND | Ventricular septal defect | ND | No | . Sacral dimple  . Failure to thrive | NF1 variant c.5426G>T, p.Arg1809Cys (exon 38) |
|  | 154 | ND | F | 5-8 | ND | ND | . Hypertelorism  . Low set ears  . Low posterior hair line | CLS | No | Multifocal cortical dysplasia | No | No | No | . Webbed neck  . Ectopic right kidney | NF1 variant c.5426G>T, p.Arg1809Cys (exon 38)  PTPN11 testing negative |
| 2016/Vuralli (J Clin Res Pediatr Endocrinol) | 155 | Familial | F | 13 | Yes | Yes | . Hypertelorism  . Downslanted palpebral fissures  . Mid-facial hypoplasia  . Prominent nasolabial folds  . Low-set and posteriorly rotated ears  . Low nasal root  . Low posterior hairline | CLS, L | No | . Anterior pituaitary hypoplasia  . Hyperintense signals | Echocardiography normal | No | . Cubitus valgus  . Brachy- and clinodactyly | . Short and webbed neck  . Growth hormone deficiency  . Widely space nipples | NF1 c.7846C>T, pArg2616*  PTPN11 testing negative |
|  | 156 | Familial | M | ND | Yes | ND | . Prominent nasolabial folds,  . Low nasal root  . Low-set ears | CLS | No | ND | ND | ND | Cubitus valgus | Short neck | NF1 c.7846C>T, pArg2616*  PTPN11 normal |
| 2016/Yapijakis (in Vivo) | 157 | Familial | M | 40 | ND | ND | ND | CLS, SNF | Epileptic crises (grand mal) | Mass of the right frontotemporal region | No | ND | . Osseous lesion left parietal bone  . Hyperostosis of ulna in both hands | No | NF1 p.R1947* (exon 31, truncating)  PTPN11 normal |
|  | 158 | Familial | M | 8 | ND | Yes | . Hypertelorism  . Low-set ears  . Low nasal bridge  . Highly arched palate  . Increased width of mouth | CLS, SNF | Moderate mental deficiency | ND | No | ND | No | . Myopia  . Strabismus | NF1 p.R1947* (exon 31, truncating)  PTPN11 normal |

| **Table S1. Epidemiological characteristics, clinical manifestations and molecular diagnostics in series or case reports of neurofibromatosis-Noonan syndrome previously reported (continued)** | | | | | | | | | | | | | | | |  |
| --- | --- | --- | --- | --- | --- | --- | --- | --- | --- | --- | --- | --- | --- | --- | --- | --- |
| Year/Reference | Case number | NF1 family history | Sex | Age (y) | Macrocrania | Short stature | Facial Noonan phenotype | Skin manifestations | Neurological manifestations | RMI and/or CT-scan abnormalities | Cardiac manifestations | Lisch nodules | Osteoarticular manifestations | Other findings/manifestations | Molecular diagnosis | |

| 2016/Yapijakis (in Vivo) | 159 | Familial | M | 7 | ND | Yes | . Hypertelorism  . Low-set ears  . Low nasal bridge  . Highly arched palate  . Increased width of mouth | CLS, SNF | Moderate mental deficiency | ND | No | ND | No | Hypoplastic distal phalanges and nails of hands | NF1 p.R1947* (exon 31, truncating)  PTPN11 normal |
| --- | --- | --- | --- | --- | --- | --- | --- | --- | --- | --- | --- | --- | --- | --- | --- |
| 2018/Koczkowska (Am J Hum Genet) | 160 | Proven sporadic | F | 14-18 | No | Yes | . Hypertelorism  . Down-slanting palpebral fissures | CLS, L, SNF, PNF | School for blind | MRI: Optic glioma (nerve, chiasm) | . Pulmonary stenosis  . Aortic coarctation | ND | . Pectus carinatum  . Bowed long bones | No | NF1 c.2531T>G, p.Leu844Arg  PTPN11 c.1529A>G, p.Gln510Arg (previously reported by Bertola (2005)) |
|  | 161 | Familial | F | >26 | ND | ND | . Hypertelorism  . Bilateral epicanthus | CLS, L, SNF, SCNF | No | ND | No | Yes | Scoliosis | Cervical neurofibroma (C1-C2) | NF1 c.2531T>G, p.Leu844Arg |
|  | 162 | Proven sporadic | M | 14-18 | No | No | Ptosis | CLS (<6), PNFs | Attention deficit disorder | No optic glioma | Hypertension | ND | Scoliosis | Spinal neurofibroma | NF1 c.2534G>A, p.Cys845Tyr |
|  | 163 | Reported sporadic | F | 5-8 | Yes | No | . Ptosis  . Grey irisses | CLS | No | MRI: Optic glioma | Hypertension | Yes | No | . Precocious puberty  . Juvenile xanthogranuloma | NF1 c.2540T>G, p.Leu847Arg |
|  | 164 | Proven sporadic | F | 5-8 | ND | No | . Hypertelorism  . Ptosis  . Bitemporal narrowing  . Trigonocephaly | CLS, PNF | . Abnormal development  . Gross, fine motor delays  . Learning disabilities  . Attention deficit hyperactivity disorder  . Speech delay | ND | No | ND | Scoliosis | Spinal neurofibroma (T8-T11) | NF1 c.2540T>G, p.Leu847Arg |
|  | 165 | Reported sporadic | M | >26 | Yes | No | . Hypertelorism  . Down-slanting palpebral fissures  . Prominent nose | CLS, L | . Abnormal development  . Learning disabilities  . Attention deficit hyperactivity disorder  . Speech delay | No optic glioma | No | No | . Scoliosis  . Pectus excavatum  . Lumbar vertebral scalloping  . Spina bifida occulta (S1-S2) | .Spitz nevus  . Esotropia (left eye)  . Amblyopa  . Astigmatism | NF1 c.2540T>C, p.Leu847Pro  PTPN11 and SPRED1 testing negative |
|  | 166 | Reported sporadic | M | 0-2 | No | Yes | . Midface hypoplasia  . Low set ears | CLS, L | No | ND | Hypertrophic cardiomyopathy | ND | No | No | NF1 c.2540T>C, p.Leu847Pro  PTPN11 and SPRED1 testing negative |
|  | 167 | Reported sporadic | M | 19-26 | Yes | Yes | Hypertelorism | CLS, L, SNF | . Learning disabilities  . Attention deficit disorder  . Autism spectrum | No optic glioma | No | ND | . Scoliosis  . Cherubism | Rectal mass | NF1 c.2540T>C, p.Leu847Pro  PTPN11 and SPRED1 testing negative |
|  | 168 | Familial | M | 19-26 | Yes | No | . Ptosis  . Low set ears  . Down-slanting palpebral fissures | CLS, L, SNF SCNF, PNF | Abnormal development delay | Optic glioma (?) | No | ND | Scoliosis | . Non ossifying fibroma (femur/knee)  . Spinal neurofibroma (L4-L5, T11-12) | NF1 c.2540T>C, p.Leu847Pro  KIT missense alteration c.1195G>A, p.Val399Ile |
|  | 169 | Proven sporadic | M | 5-8 | No | Yes | Hypertelorism | CLS (<6) | Learning disabilities | No optic glioma | No | - | No | No | NF1 c.2543G>A ;4463G>A, p.Gly848Glu, p.Arg1488His  PTPN11 testing negative |
| 2018/Trevisson (Mol Genet Genomic Med) | 170 | Familial | F | 10 | No | No | . Hypertelorism  . Down-slanting palpebral fissures | CLS | No learning disabilities | No optic glioma | No pulmonary stenosis  Echocardiography normal | ND | No | No | NF1 c.3112A>G, p.Arg1038Gly  BRAF, CBL, CDC42, HRAS, KRAS, LZTR1, MAP2K1, MAP2K2, MAP3K8, MYST4, NRAS, PTPN11, RAF1, RASA2, RIT1, RRAS, SHOC2, SOS1, SOS2, SPRED1, SPRY1: no variant |
|  | 171 | Familial | M | 2 | Yes | No | . Frontal bossing  . Hypertelorism  . Down-slanting palpebral fissures  . Posteriorly rotated ears  . Mild retrognathia | CLS | No | No optic glioma | No pulmonary stenosis  Echocardiography normal | ND | No | No | NF1 c.3112A>G, p.Arg1038Gly  BRAF, CBL, CDC42, HRAS, KRAS, LZTR1, MAP2K1, MAP2K2, MAP3K8, MYST4, NRAS, PTPN11, RAF1, RASA2, RIT1, RRAS, SHOC2, SOS1, SOS2, SPRED1, SPRY1: no variant |

| **Table S1. Epidemiological characteristics, clinical manifestations and molecular diagnostics in series or case reports of neurofibromatosis-Noonan syndrome previously reported (continued)** | | | | | | | | | | | | | | | |  |
| --- | --- | --- | --- | --- | --- | --- | --- | --- | --- | --- | --- | --- | --- | --- | --- | --- |
| Year/Reference | Case number | NF1 family history | Sex | Age (y) | Macrocrania | Short stature | Facial Noonan phenotype | Skin manifestations | Neurological manifestations | RMI and/or CT-scan abnormalities | Cardiac manifestations | Lisch nodules | Osteoarticular manifestations | Other findings/manifestations | Molecular diagnosis | |

| 2018/Trevisson (Mol Genet Genomic Med) | 172 | Familial | F | 41 | No | No | . Hypertelorism  . Down-slanting palpebral fissures | CLS, L | No learning disabilities | No optic glioma | No pulmonary stenosis  Echocardiography normal | ND | No | No | NF1 c.3112A>G, p.Arg1038Gly  BRAF, CBL, CDC42, HRAS, KRAS, LZTR1, MAP2K1, MAP2K2, MAP3K8, MYST4, NRAS, PTPN11, RAF1, RASA2, RIT1, RRAS, SHOC2, SOS1, SOS2, SPRED1, SPRY1: no variant |
| --- | --- | --- | --- | --- | --- | --- | --- | --- | --- | --- | --- | --- | --- | --- | --- |
|  | 173 | Familial | F | 67 | No | No | . Hypertelorism  . Down-slanting palpebral fissures | CLS | No learning disabilities | No optic glioma | No pulmonary stenosis | ND | No | No | NF1 c.3112A>G, p.Arg1038Gly  BRAF, CBL, CDC42, HRAS, KRAS, LZTR1, MAP2K1, MAP2K2, MAP3K8, MYST4, NRAS, PTPN11, RAF1, RASA2, RIT1, RRAS, SHOC2, SOS1, SOS2, SPRED1, SPRY1: no variant |
|  | 174 | Sporadic | F | 30 | Yes | Yes | . Hypertelorism  . Down-slanting palpebral fissures | CLS, L | No learning disabilities | FASIs (lenticular at brain MRI) | No pulmonary stenosis  Echocardiography normal | ND | No | . Pubertal delay  . Lipoma (back) | NF1 c.3112A>G, p.Arg1038Gly  BRAF, CBL, CDC42, HRAS, KRAS, LZTR1, MAP2K1, MAP2K2, MAP3K8, MYST4, NRAS, PTPN11, RAF1, RASA2, RIT1, RRAS, SHOC2, SOS1, SOS2, SPRED1, SPRY1: no variant |
| 2019/Baquedano Lobera (BMC Pediatrics) | 175 | Sporadic | F | 3 | Yes | Yes | . Prominent forehead  . Hypertelorism  . Ptosis  . Down-slanting palpebral fissures  . Low earlobes  . Long philtrum | CLS | Axial hypotonia | ND | Pulmonary valvular stenosis | ND | Skeletal dysplasia  Short metacarpals  Rhizomelic shortness of lower limbs, arched legs | Separated nipples | NF1 c.7756G>T, p.E2586* (exon 53)  KRAS p.Val14Ile |
| 2019/Koczkowska (Genet Med) | 176 | ND | F | 19-26 | Yes | No | . Midface hypoplasia  . Low set ears | CLS, L | Abnormal (FSQI 48) | ND | No | ND | Winging of the scapula | Webbed neck | NF1 c.2970_2972del, p.Met992del  PTPN11 and SPRED1 testing negative |
|  | 177 | Reported sporadic | M | 14-18 | Yes | No | . Frontal bossing  . Bitemporal narrowing  . Hypertelorism  . Downslanting palpebral fissures | CLS, L | . Learning disabilities  . Motor apraxia | No optic glioma (MRI) | No | No | No | Oligodendroglioma | NF1 c.2970_2972del, p.Met992del  PTPN11 and SPRED1 testing negative |
|  | 178 | Familial | M | 9-13 | No | Yes | . Hypertelorism  . Midface hypoplasia | CLS, L | Learning disabilities | ND | ND | Yes | Pectus excavatum | No | NF1 c.2970_2972del, p.Met992del  PTPN11 and SPRED1 testing negative |
|  | 179 | Familial | M | 9-13 | No | Yes | . Hypertelorism  . Large mouth  . Blue irides | CLS, L | Severe intellectual disability | No optic glioma (MRI) | No | No | Scoliosis  Pectus carinatum | Delayed puberty | NF1 c.2970_2972del, p.Met992del  PTPN11 and SPRED1 testing negative  aCGH: copy number abnormalities: 1q21.1 deletion and 12q23.2 duplication |
|  | 180 | Familial | F | >26 | No | - | . Hypertelorism  . Blue irides | CLS, L | Learning disabilities | ND | No | No | No | No | NF1 c.2970_2972del, p.Met992del  PTPN11 and SPRED1 testing negative  Deletion 1q21.1 |
|  | 181 | Reported sporadic | M | 9-13 | No | Yes | . Hypertelorism  . Ptosis  . Square upper helices and fleshy lobes | CLS | No | ND | Pulmonary stenosis | No | Prominent upper sternum with concavity of lower sternum | No | NF1 c.2970_2972del, p.Met992del  PTPN11 and SPRED1 testing negative |
|  | 182 | ND | M | >26 | Yes | No | . Hypertelorism  . Midface hypoplasia | CLS, L | . Learning disabilities  . Attention deficit hyperactivity disorder  . Speech delay | ND | ND | ND | No | Webbed neck | NF1 c.2970_2972del, p.Met992del  PTPN11 and SPRED1 testing negative |
|  | 183 | Familial | F | 5-8 | No | Yes | Low set ears | CLS, L | No | ND | Pulmonary stenosis | Yes | No | No | NF1 c.2970_2972del, p.Met992del  PTPN11 and SPRED1 testing negative |
|  | 184 | Familial | F | 5-8 | No | No | . Mild frontal bossing  . Epicanthal folds (bilateral)  . Wide nasal bridge | CLS | Speech delay | No optic glioma (MRI) | No | No | No | Sensorineural hearing loss  Large Mongolian spots  Delta-beta thalassemia | NF1 c.2970_2972del, p.Met992del  SPRED1 testing negative |

| **Table S1. Epidemiological characteristics, clinical manifestations and molecular diagnostics in series or case reports of neurofibromatosis-Noonan syndrome previously reported (continued)** | | | | | | | | | | | | | | | |  |
| --- | --- | --- | --- | --- | --- | --- | --- | --- | --- | --- | --- | --- | --- | --- | --- | --- |
| Year/Reference | Case number | NF1 family history | Sex | Age (y) | Macrocrania | Short stature | Facial Noonan phenotype | Skin manifestations | Neurological manifestations | RMI and/or CT-scan abnormalities | Cardiac manifestations | Lisch nodules | Osteoarticular manifestations | Other findings/manifestations | Molecular diagnosis | |

| 2019/Koczkowska (Genet Med) | 185 | Reported sporadic | F | >5 | No | Yes | Hypertelorism | CLS, L | . Learning disabilities  . Mild developmental delay | ND | No | ND | Scoliosis | Pilomyxoid astrocytoma of thoracic spine | NF1 c.2970_2972del, p.Met992del  PTPN11 and SPRED1 testing negative |
| --- | --- | --- | --- | --- | --- | --- | --- | --- | --- | --- | --- | --- | --- | --- | --- |
|  | 186 | ND | F | 2-4 | No | No | . Downslanting palpebral fissures  . Low set ears | CLS, L | No | - | ND | No | No | No | NF1 c.2970_2972del, p.Met992del  PTPN11 and SPRED1 testing negative |
|  | 187 | Reported sporadic | F | 14-18 | Yes | No | . Hypertelorism  . Downslanting palpebral fissures | CLS, L | Learning disabilities | No optic glioma (MRI) | No | No | No | No | NF1 c.2970_2972del, p.Met992del |
|  | 188 | Familial | F | 5-8 | No | Yes | . Hypertelorism  . Downslanting palpebral fissures  . Epicanthic folds  . Low-set posteriorly rotated ears  . Low posterior hairline | CLS, L | Learning disabilities | No optic glioma (MRI) | Pulmonary stenosis | Yes | Scoliosis | Webbed neck | NF1 c.2970_2972del, p.Met992del  PTPN11 testing negative  Previously reported by De Luca (2005) |
|  | 189 | Familial | F | >26 | No | Yes | . Hypertelorism  . Ptosis  . Downslanting palpebral fissures  . Midface hypoplasia  . Low-set posteriorly rotated ears  . Low posterior hairline | CLS, L | No | ND | No | Yes | . Scoliosis  . Pectus abnormality | . Webbed neck  . Cryptorchidism  . Neuroblastoma | NF1 c.2970_2972del, p.Met992del  PTPN11 testing negative  Previously reported by De Luca (2005) |
|  | 190 | Reported sporadic | M | >26 | ND | No | . Hypertelorism  . Ptosis  . Downslanting palpebral fissures  . Low set ears  . Low posterior hairline  . Blue irides | CLS | . Learning disabilities  . Psychiatric problems | No optic glioma (MRI) | No | No | No | . Multiple subcutaneous lipomas  . Gastro-esophageal reflux  . Postural tremor of hands  . Bilateral carpal tunnel syndrome | NF1 c.2970_2972del, p.Met992del |
| 2019/Pinna (Genes) | 191 | Sporadic | F | ND | ND | Yes | No detail (Noonan syndrome facial features) | CLS, L, SNF | No | No optic glioma | . Pulmonary valve stenosis  . Pulmonary valve dysplasia  . Mitral valve prolapse | Yes | No | Leukemia | NF1 c.499_502delTGTT, p.Cys167GlnfsTer10 (frameshift) |
|  | 192 | Familial | F | 15 | ND | Yes | No detail (Noonan syndrome facial features) | CLS, L, SNF, SCNF | Learning disabilities | No optic glioma | Pulmonary valve stenosis | Yes | Scoliosis | No | NF1 c.908T>C, p.Leu303Pro (missense/in frame indel) |
|  | 193 | Familial | M | 10 | ND | Yes | No detail (Noonan syndrome facial features) | CLS, L | Learning disabilities | No optic glioma | Mitral valve insufficiency  Tricuspid valve insufficiency | ND | No | No | NF1 c.1246C>T, p.Arg416Ter (nonsense) |
|  | 194 | ND | M | 11 | ND | No | No detail (Noonan syndrome facial features) | CLS, L | No | No optic glioma | Mitral valve insufficiency | ND | No | No | NF1 c.1246C>T, p.Arg416Ter (nonsense) |
|  | 195 | Sporadic | F | 3 | ND | No | No detail (Noonan syndrome facial features) | CLS | No | ND | Patent foramen ovale | No | No | No | NF1 c.1541_1542delAG, p.Gln514ArgfsTer43 (frameshift) |
|  | 196 | ND | F | 15 | ND | Yes | No detail (Noonan syndrome facial features) | CLS, L, SNF, SCNF | No | No optic glioma | Atrial septal defect ostium secundum | No | No | No | NF1 c.1603C>T, p.Gln535Ter (nonsense) |
|  | 197 | Sporadic | M | 3 | ND | No | No detail (Noonan syndrome facial features) | CLS, L | Learning disabilities | Optic glioma | Pulmonary valve stenosis | No | No | No | NF1 c.1642G>C, p.Ala548Pro (missense/in frame indel) |
|  | 198 | Sporadic | M | 6 | No | Yes | No detail (Noonan syndrome facial features) | CLS | Learning disabilities | No optic glioma | Pulmonary valve stenosis | No | No | No | NF1 c.1756_1759delACTA, p.Thr586ValfsTer18 (frameshift) |
|  | 199 | Sporadic | F | 6 | Yes | No | No detail (Noonan syndrome facial features) | CLS, L | No | No optic glioma | Pulmonary valve stenosis | No | No | Popliteal schwannoma | NF1 c.1862delC, p.Cys622ValfsTer9 (frameshift) |

| **Table S1. Epidemiological characteristics, clinical manifestations and molecular diagnostics in series or case reports of neurofibromatosis-Noonan syndrome previously reported (continued)** | | | | | | | | | | | | | | | |  |
| --- | --- | --- | --- | --- | --- | --- | --- | --- | --- | --- | --- | --- | --- | --- | --- | --- |
| Year/Reference | Case number | NF1 family history | Sex | Age (y) | Macrocrania | Short stature | Facial Noonan phenotype | Skin manifestations | Neurological manifestations | RMI and/or CT-scan abnormalities | Cardiac manifestations | Lisch nodules | Osteoarticular manifestations | Other findings/manifestations | Molecular diagnosis | |

| 2019/Pinna (Genes) | 200 | ND | M | 8 | ND | Yes | No detail (Noonan syndrome facial features) | CLS, L | Learning disabilities | Optic glioma | Patent foramen ovale | No | No | No | NF1 c.2330G>A, p.Trp777Ter (missense/in frame indel) |
| --- | --- | --- | --- | --- | --- | --- | --- | --- | --- | --- | --- | --- | --- | --- | --- |
|  | 201 | ND | F | 12 | ND | No | No detail (Noonan syndrome facial features) | CLS, L | No | ND | . Mitral valve dysplasia  . Bicuspid aortic valve | No | No | No | NF1 C.2410-1G>C (splicing) |
|  | 202 | Familial | F | 13 | No | Yes | No detail (Noonan syndrome facial features) | CLS, L | Learning disabilities | No optic glioma | Pulmonary valve stenosis | Yes | Scoliosis | Myopia | NF1 c.2970_2972delAAT, p.Met992del (missense/in frame indel) |
|  | 203 | ND | F | 8 | ND | Yes | No detail (Noonan syndrome facial features) | CLS, L | No | No optic glioma | Pulmonary valve stenosis | No | Scoliosis | No | NF1 c.3113+1G>A (splicing) |
|  | 204 | ND | M | 33 | ND | Yes | No detail (Noonan syndrome facial features) | CLS, L, SNF, SCNF | Learning disabilities | No optic glioma | Pulmonary valve stenosis | No | Scoliosis | Myelofibrosis | NF1 c.3445A>G, p.Met1149Val (missense/in frame indel) |
|  | 205 | ND | F | ND | ND | No | No detail (Noonan syndrome facial features) | CLS | No | No optic glioma | Pulmonary valve stenosis | No | No | No | NF1 c.3587T>G, p.Leu1196Arg (missense/in frame indel) |
|  | 206 | Familial | F | 3 | No | Yes | No detail (Noonan syndrome facial features) | CLS | Learning disabilities | ND | . Pulmonary valve stenosis  . Atrial septal defect | ND | No | No | NF1 c.3635delT, p.Val1212AlafsTer3 (frameshift) |
|  | 207 | Familial | F | 3 | No | Yes | No detail (Noonan syndrome facial features) | CLS | Learning disabilities | ND | . Pulmonary valve stenosis  . Atrial septal defect | ND | No | No | NF1 c.3635delT, p.Val1212AlafsTer3 (frameshift) |
|  | 208 | ND | M | ND | ND | No | No detail (Noonan syndrome facial features) | CLS, L, SNF, SCNF PNF | Learning disabilities | No optic glioma | Mitral valve dysplasia | No | No | No | NF1 c.3974+1G>A (splicing) |
|  | 209 | Sporadic | F | 3 | ND | ND | No detail (Noonan syndrome facial features) | CLS, L | No | No optic glioma | Pulmonary valve stenosis | No | No | No | NF1 c.4076delC, p.Pro1359LeufsTer26 (frameshift) |
|  | 210 | Sporadic | F | 54 | Yes | Yes | No detail (Noonan syndrome facial features) | CLS, L, SNF, PNF | No | Optic glioma | Mitral valve prolapse | Yes | No | Thoracic anomalies | NF1 c.4107C>A, p.Tyr1369Ter (nonsense) |
|  | 211 | Sporadic | M | 21 | ND | Yes | No detail (Noonan syndrome facial features) | CLS, L | No | ND | . Pulmonary valve dysplasia  . Aortic valve dysplasia | No | No | Pilomatrixoma | NF1 c.4160C>A, p.Ala1387Asp (missense/in frame indel) |
|  | 212 | Familial | F | 13 | ND | Yes | No detail (Noonan syndrome facial features) | CLS, L | Learning disability | ND | Pulmonary valve stenosis | No | No | No | NF1 c.4173A>T, p.Arg1391Ser (missense/in frame indel) |
|  | 213 | Familial | F | 7 | ND | No | No detail (Noonan syndrome facial features) | CLS, L | No | ND | Pulmonary valve stenosis | No | No | No | NF1 c.4173A>T, p.Arg1391Ser (missense/in frame indel) |
|  | 214 | Familial | F | 18 | Yes | No | No detail (Noonan syndrome facial features) | CLS, L SNF | No | No optic glioma | Mitral valve prolapse | Yes | Scoliosis | No | NF1 c.4243G>T, p.Glu1415Ter (nonsense) |
|  | 215 | Sporadic | M | 17 | Yes | No | No detail (Noonan syndrome facial features) | CLS, L | Learning disability | No optic glioma | Pulmonary valve stenosis | Yes | No | No | NF1 c.4267A>G***, p.Lys1423Glu (missense/in frame indel) |
|  | 216 | Sporadic | F | 27 | Yes | No | No detail (Noonan syndrome facial features) | CLS | No | No optic glioma | Atrial septal defect | No | No | No | NF1 c.4289A>C, p.Asn1430Thr (missense/in frame indel) |
|  | 217 | Familial | F | 6 | ND | No | No detail (Noonan syndrome facial features) | CLS, L | Learning disability | ND | Pulmonary valve stenosis | ND | ND | No | NF1 c.4306A>G, p.Lys1436Glu (missense/in frame indel) |
|  | 218 | Sporadic | F | 9.5 | Yes | Yes | No detail (Noonan syndrome facial features) | CLS, L | No | ND | Pulmonary valve stenosis | Yes | Scoliosis | No | NF1 c.4312_4314delGAA, p.Glu1438del (missense/in frame indel) |

| **Table S1. Epidemiological characteristics, clinical manifestations and molecular diagnostics in series or case reports of neurofibromatosis-Noonan syndrome previously reported (continued)** | | | | | | | | | | | | | | | |  |
| --- | --- | --- | --- | --- | --- | --- | --- | --- | --- | --- | --- | --- | --- | --- | --- | --- |
| Year/Reference | Case number | NF1 family history | Sex | Age (y) | Macrocrania | Short stature | Facial Noonan phenotype | Skin manifestations | Neurological manifestations | RMI and/or CT-scan abnormalities | Cardiac manifestations | Lisch nodules | Osteoarticular manifestations | Other findings/manifestations | Molecular diagnosis | |

| 2019/Pinna (Genes) | 219 | Sporadic | M | 9 | Yes | No | No detail (Noonan syndrome facial features) | CLS, L | No | ND | Pulmonary valve stenosis | No | No | No | NF1 c.4493G>A, p.Gly1498Glu (missense/in frame indel) |
| --- | --- | --- | --- | --- | --- | --- | --- | --- | --- | --- | --- | --- | --- | --- | --- |
|  | 220 | Sporadic | F | 38 | No | Yes | No detail (Noonan syndrome facial features) | CLS, L SNF, PNF | No | ND | Mitral valve thickening | ND | No | Thoracic anomalies | NF1 c.5339T>G, p.Leu1780Ter (nonsense) |
|  | 221 | ND | M | 18 | ND | No | No detail (Noonan syndrome facial features) | CLS, L, SNF, SCNF, PNF | Learning disability | Optic glioma | Mitral valve prolapse | Yes | No | No | NF1 c.5705C>G, p.Thr1902Arg (missense/in frame indel) |
|  | 222 | Sporadic | M | 22 | Yes | No | No detail (Noonan syndrome facial features) | CLS | ND | ND | Mitral valve prolapse | Yes | Scoliosis | No | NF1 c.6709C>T, p.Arg2237Ter (nonsense) |
|  | 223 | ND | F | 10 | ND | No | No detail (Noonan syndrome facial features) | CLS, L | No | ND | . Mitral valve prolapse  . Mitral valve insufficiency | No | No | No | NF1 c.6789_6792delTTAC, p.Tyr2264ThrfsTer5 (frameshift) |
|  | 224 | Sporadic | M | 2 | ND | ND | No detail (Noonan syndrome facial features) | CLS, L | No | Optic glioma | Pulmonary valve stenosis | No | No | No | NF1 c.7291C>T, p.Arg2431Cys (missense/in frame indel) |
|  | 225 | ND | F | 17 | ND | Yes | No detail (Noonan syndrome facial features) | CLS, L, NF | Learning disability | No optic glioma | Mitral valve insufficiency | Yes | Scoliosis | . Hypertension  . Renal artery stenosis | NF1 c.7486C>T, p.Arg2616Ter (nonsense) |
|  | 226 | ND | M | 6 | ND | Yes | No detail (Noonan syndrome facial features) | CLS, L | No | ND | Patent ductus arteriosus | No | Scoliosis | No | NF1 7846C>T, p.Arg2616Ter (nonsense) |
|  | 227 | Familial | F | ND | ND | Yes | No detail (Noonan syndrome facial features) | CLS, L, NF-C, NF-SC | . Learning disability  . Intellectual disability | No optic glioma | Polyvalvular dysplasia with mild interventricular septal hypertrophy | Yes | No | No | NF1 (whole gene deletion) |
| 2020/Koczkowska (Hum Mutation) | 228 | Familial | M | <2 | No | Yes | . Midface hypoplasia  . Low set ears | CLS (<6) | . Motor delay  . Speech delay | ND | No | No | No | No | NF1 c.3445A>G, p.Met1149Val |
|  | 229 | Proven Sporadic | F | <2 | Yes | No | . Coarse look  . Hypertelorism  . Low posterior hairline | CLS, L | Hydrocephalus | ND | No | No | No | No | NF1 c.3445A>G, p.Met1149Val  PTPN11, SOS1, SOS2, RAF1, BRAF, SHOC2, CBL, KRAS, MAP2K2, PPP1CB, RRAS, RIT1, HRAS, MAP2K1, RASA2, SPRED1: no variant |
|  | 230 | Unknown | M | 5-8 | Yes | Yes | Low set ears | CLS, L | Speech delay | No optic glioma (MRI) | Atrial septal defect | No | . Sphenoid swing dysplasia  . Scoliotic attitude | No | NF1 c.3445A>G, p.Met1149Val |
|  | 231 | Reported sporadic | F | 5-8 | Yes | No | . Coarse face  . High frontal hairline  . Frontal bossing  . Bitemporal narrowing  . Downslanting palpebral fissures  . Bulbous nasal tip | CLS, L, P-NF | No | No optic glioma (MRI) | Atrial septal defect | No | Sphenoid dysplasia | . Sacral plan angioma  . Right duplex kidney | NF1 c.3445A>G, p.Met1149Val  PTPN11, SOS1, SOS2, RAF1, BRAF, SHOC2, CBL, KRAS, MAP2K2, PPP1CB, RRAS, RIT1, HRAS, MAP2K1, RASA2, SPRED1: no variant |
|  | 232 | Familial | M | 5-8 | No | No | Hypertelorism | CLS, L | No | ND | No | ND | Pectus excavatum | No | NF1 c.3445A>G, p.Met1149Ile  PTPN11, SOS1, SOS2, RAF1, BRAF, SHOC2, CBL, KRAS, MAP2K2, PPP1CB, RRAS, RIT1, HRAS, MAP2K1, RASA2, SPRED1: no variant |
|  | 233 | Familial | M | 5-8 | No | Yes | Hypertelorism | CLS | . Headaches  . Speech delay  . Learning disability | No optic glioma (MRI) | No | ND | . Joint hypermobility  . Mild flat feet | No | NF1 c.3445A>G, p.Met1149Ile  SPRED1 : no variant |

| **Table S1. Epidemiological characteristics, clinical manifestations and molecular diagnostics in series or case reports of neurofibromatosis-Noonan syndrome previously reported (continued)** | | | | | | | | | | | | | | | |  |
| --- | --- | --- | --- | --- | --- | --- | --- | --- | --- | --- | --- | --- | --- | --- | --- | --- |
| Year/Reference | Case number | NF1 family history | Sex | Age (y) | Macrocrania | Short stature | Facial Noonan phenotype | Skin manifestations | Neurological manifestations | RMI and/or CT-scan abnormalities | Cardiac manifestations | Lisch nodules | Osteoarticular manifestations | Other findings/manifestations | Molecular diagnosis | |

| 2020/Koczkowska (Hum Mutation) | 234 | Familial | M | 5-8 | No | No | Ptosis | CLS | . Speech delay  . Learning disability | ND | No | ND | . Pectus excavatum  . Genu valgum  . Clinodactyly | Webbed neck | NF1 c.3445A>G, p.Met1149Ile  PTPN11, SOS1, SOS2, RAF1, BRAF, SHOC2, CBL, KRAS, MAP2K2, PPP1CB, RRAS, RIT1, HRAS, MAP2K1, RASA2, SPRED1: no variant |
| --- | --- | --- | --- | --- | --- | --- | --- | --- | --- | --- | --- | --- | --- | --- | --- |
|  | 235 | Familial | M | 9-13 | Yes | No | . Hypertelorism  . Ptosis  . Downslanting palpebral fissures  . Midface hypoplasia  . Low set ears | CLS, L | . Learning disability  . Fine motor delay | No optic glioma (MRI) | No | ND | No | No | NF1 c.3445A>G, p.Met1149Val |
|  | 236 | Familial | M | 9-13 | Yes | No | . Hypertelorism  . Midface hypoplasia  . Low set ears | CLS (<6) | No | ND | No | ND | No | No | NF1 c.3445A>G, p.Met1149Val |
|  | 237 | Familial | M | 9-13 | No | No | . Hypertelorism  . Downslanting palpebral fissures | CLS | . Headaches  . Speech delay  . Learning disability | No optic glioma (MRI) | No | No | Pectus excavatum | Musculoskeletal pains | NF1 c.3445A>G, p.Met1149Ile |
|  | 238 | Familial | M | 14-18 | No | Yes | . Hypertelorism  . Ptosis  . Bitemporal narrowing  . Dolichocephaly  . Downslanting palpebral fissures  . Midface hypoplasia  . Low set ears | CLS, L, SNF, SCNF | . Epilepsy  . Fine motor delay  . Learning disability | No optic glioma (MRI) | No | ND | Pectus excavatum | No | NF1 c.3445A>G, p.Met1149Val  PTPN11, SOS1, SOS2, RAF1, BRAF, SHOC2, CBL, KRAS, MAP2K2, PPP1CB, RRAS, RIT1, HRAS, MAP2K1, RASA2, SPRED1: no variant |
|  | 239 | Familial | M | 14-18 | Yes | No | . Hypertelorism  . Midface hypoplasia  . Low set ears | CLS, L | Learning disability | ND | No | No | No | Webbed neck | NF1 c.3445A>G, p.Met1149Val  PTPN11, SOS1, SOS2, RAF1, BRAF, SHOC2, CBL, KRAS, MAP2K2, PPP1CB, RRAS, RIT1, HRAS, MAP2K1, RASA2, SPRED1: no variant |
|  | 240 | Familial | M | 14-18 | ND | ND | Possible (no details) | CLS, L | Learning disability | ND | ND | ND | No | . Pilomatrixoma  . Marfanoid habitus | NF1 c.3445A>G, p.Met1149Val |
|  | 241 | Reported sporadic | M | 14-18 | No | No | . Ptosis  . Low-set ears | CLS, L | . Speech delay  . Learning disability | No optic glioma (MRI) | No | No | Scoliotic attitude | . Splenomegaly  . Achromic nevus | NF1 c.3445A>G, p.Met1149Val |
|  | 242 | Familial | F | 19-26 | ND | ND | . Hypertelorism  . Midface hypoplasia  . Low set ears | CLS, L | . Speech delay  . Learning disability | ND | No | ND | No | . Webbed neck | NF1 c.3445A>G, p.Met1149Val  PTPN11, SOS1, SOS2, RAF1, BRAF, SHOC2, CBL, KRAS, MAP2K2, PPP1CB, RRAS, RIT1, HRAS, MAP2K1, RASA2, SPRED1: no variant |
|  | 243 | Familial | F | >26 | ND | ND | . Hypertelorism  . Ptosis  . Downslanting palpebral fissures  . Midface hypoplasia  . Low set ears | CLS | Learning disability | ND | No | ND | No | No | NF1 c.3445A>G, p.Met1149Val |
|  | 244 | Unknown | M | >26 | ND | ND | Possible (no details) | CLS, L, SNF, SCNF | Learning disability | ND | No | ND | Pectus excavatum | . Poor vision  . Strabism | NF1 c.3445A>G, p.Met1149Val |
|  | 245 | ND | F | >26 | ND | No | . Hypertelorism  . Midface hypoplasia  . Low set ears | CLS (<6) | No | ND | No | ND | No | Lipomas | NF1 c.3445A>G, p.Met1149Val |
|  | 246 | Reported sporadic | M | >26 | Yes | No | . Ptosis  . Downslanting palpebral fissures | CLS, L, SNF-C | Learning disability | No optic glioma (MRI) | Hypertrophic cardiomyopathy | ND | Pectus excavatum | . Short neck  . Wide-set nipples  . Recurrent kidney stones  . Esotropia | NF1 c.3445A>G, p.Met1149Val  PTPN11, SOS1, SOS2, RAF1, BRAF, SHOC2, CBL, KRAS, MAP2K2, PPP1CB, RRAS, RIT1, HRAS, MAP2K1, RASA2, SPRED1: no variant |

| **Table S1. Epidemiological characteristics, clinical manifestations and molecular diagnostics in series or case reports of neurofibromatosis-Noonan syndrome previously reported (continued)** | | | | | | | | | | | | | | | |  |
| --- | --- | --- | --- | --- | --- | --- | --- | --- | --- | --- | --- | --- | --- | --- | --- | --- |
| Year/Reference | Case number | NF1 family history | Sex | Age (y) | Macrocrania | Short stature | Facial Noonan phenotype | Skin manifestations | Neurological manifestations | RMI and/or CT-scan abnormalities | Cardiac manifestations | Lisch nodules | Osteoarticular manifestations | Other findings/manifestations | Molecular diagnosis | |

| 2020/Koczkowska (Hum Mutation) | 247 | ND | F | >26 | Yes | ND | . Frontal bossing  . Hypertelorism  . Ptosis  . Low set ears | CLS, L | Seizure disorder | ND | No | ND | No | No | NF1 c.3445A>G, p.Met1149Val  PTPN11, SOS1, SOS2, RAF1, BRAF, SHOC2, CBL, KRAS, MAP2K2, PPP1CB, RRAS, RIT1, HRAS, MAP2K1, RASA2, SPRED1: no variant |
| --- | --- | --- | --- | --- | --- | --- | --- | --- | --- | --- | --- | --- | --- | --- | --- |
|  | 248 | Familial | F | >26 | No | No | . Downslanting palpebral fissures  . Low set ears  . Low posterior hairline | CLS | No | ND | No | No | Webbed neck | No | NF1 c.3445A>G, p.Met1149Thr  PTPN11, SOS1, SOS2, RAF1, BRAF, SHOC2, CBL, KRAS, MAP2K2, PPP1CB, RRAS, RIT1, HRAS, MAP2K1, RASA2, SPRED1: no variant |
|  | 249 | Familial | F | >26 | No | No | . Hypertelorism  . Downslanting palpebral fissures | CLS, L | No | ND | No | ND | No | No | NF1 c.3445A>G, p.Met1149Ile  PTPN11, SOS1, SOS2, RAF1, BRAF, SHOC2, CBL, KRAS, MAP2K2, PPP1CB, RRAS, RIT1, HRAS, MAP2K1, RASA2, SPRED1: no variant |
|  | 250 | Familial | F | >26 | No | Yes | Hypertelorism | CLS | No | ND | No | ND | No | Lipomas | NF1 c.3445A>G, p.Met1149Ile |
|  | 251 | Reported sporadic | F | <2 | Yes | No | . Upslanting palpebral fissures  . Midface hypoplasia  . Low posterior hairline | CLS | . Gross motor delay  . Speech delay | No optic glioma (MRI) | Pulmonary stenosis | - | No | . Webbed neck  . Juvenile xanthogranuloma | NF1 c.3826C>G, p.Arg1276Gly  PTPN11, SOS1, SOS2, RAF1, BRAF, SHOC2, CBL, KRAS, MAP2K2, PPP1CB, RRAS, RIT1, HRAS, MAP2K1, RASA2, SPRED1: no variant |
|  | 252 | Reported sporadic | F | <2 | No | No | . Triangular face  . Ptosis  . Downslanting palpebral fissures  . Blue irides | CLS | No | ND | No | ND | No | No | NF1 c.3827G>A, p.Arg1276Gln |
|  | 253 | Familial | M | <2 | No | No | Low set ears | CLS | No | ND | No | ND | Pectus excavatum | No | NF1 c.3827G>A, p.Arg1276Gln  PTPN11, SOS1, SOS2, RAF1, BRAF, SHOC2, CBL, KRAS, MAP2K2, PPP1CB, RRAS, RIT1, HRAS, MAP2K1, RASA2, SPRED1: no variant |
|  | 254 | Familial | F | 2-4 | No | No | . Hypertelorism  . Low set ears | CLS, L | No | ND | Pulmonary stenosis | ND | No | No | NF1 c.3827G>A, p.Arg1276Gln |
|  | 255 | Reported sporadic | F | 2-4 | ND | No | . Downslanting palpebral fissures  . Epicanthal folds  . Low set ears  . Tented upper lip | CLS, L | Mild developmental delay | ND | No | Yes | No | No | NF1 c.3827G>A, p.Arg1276Gln  PTPN11, SOS1, SOS2, RAF1, BRAF, SHOC2, CBL, KRAS, MAP2K2, PPP1CB, RRAS, RIT1, HRAS, MAP2K1, RASA2, SPRED1: no variant |
|  | 256 | Proven sporadic | F | 5-8 | No | Yes | Possible (no details) | CLS, L | Developmental delay in gross motor skills | ND | No | Yes | No | No | NF1 c.3826C>G, p.Arg1276Gly |
|  | 257 | Familial | F | 5-8 | No | No | . Hypertelorism  . Low set ears | CLS, L | No | ND | Pulmonary stenosis | No | No | No | NF1 c.3827G>A, p.Arg1276Gln  PTPN11, SOS1, SOS2, RAF1, BRAF, SHOC2, CBL, KRAS, MAP2K2, PPP1CB, RRAS, RIT1, HRAS, MAP2K1, RASA2, SPRED1: no variant |
|  | 258 | Proven sporadic | F | 5-8 | Yes | Yes | Possible (no details) | CLS, L | No | No optic glioma (MRI) | Pulmonary stenosis | Yes | Leg length discrepancy | No | NF1 c.3827G>A, p.Arg1276Gln |
|  | 259 | Reported sporadic | M | 5-8 | Yes | Yes | Possible (no details) | CLS, L | No | No optic glioma (MRI) | No | No | Pectus excavatum | No | NF1 c.3827G>A, p.Arg1276Gln |
|  | 260 | Familial | M | 5-8 | Yes | No | . Hypertelorism  . Downslanting palpebral fissures | CLS, L | No | ND | . Hypertrophic cardiomyopathy  . Ventricular septal defect | No | No | Vitiligo (?) | NF1 c.3827G>A, p.Arg1276Gln  PTPN11, SOS1, SOS2, RAF1, BRAF, SHOC2, CBL, KRAS, MAP2K2, PPP1CB, RRAS, RIT1, HRAS, MAP2K1, RASA2, SPRED1: no variant |
|  | 261 | Familial | F | 5-8 | Yes | No | . Coarse face  . Frontal bossing | CLS, L | . Attention deficit hyperactivity disorder  . Gross motor delay  . Migraines | No optic glioma (MRI) | No | Yes | Scoliosis | No | NF1 c.3827G>A, p.Arg1276Gln |
|  | 262 | Familial | F | 5-8 | No | Yes | Hypertelorism | CLS, L | No | ND | No | ND | No | No | NF1 c.3827G>A, p.Arg1276Gln |

| **Table S1. Epidemiological characteristics, clinical manifestations and molecular diagnostics in series or case reports of neurofibromatosis-Noonan syndrome previously reported (continued)** | | | | | | | | | | | | | | | |  |
| --- | --- | --- | --- | --- | --- | --- | --- | --- | --- | --- | --- | --- | --- | --- | --- | --- |
| Year/Reference | Case number | NF1 family history | Sex | Age (y) | Macrocrania | Short stature | Facial Noonan phenotype | Skin manifestations | Neurological manifestations | RMI and/or CT-scan abnormalities | Cardiac manifestations | Lisch nodules | Osteoarticular manifestations | Other findings/manifestations | Molecular diagnosis | |

| 2020/Koczkowska (Hum Mutation) | 263 | Proven sporadic | M | 5-8 | No | Yes | . Hypertelorism  . Downslanting palpebral fissures | CLS, L | No | ND | Pulmonary stenosis | No | . Pectus excavatum  . Kyphosis | No | NF1 c.3827G>A, p.Arg1276Gln  PTPN11, SOS1, SOS2, RAF1, BRAF, SHOC2, CBL, KRAS, MAP2K2, PPP1CB, RRAS, RIT1, HRAS, MAP2K1, RASA2, SPRED1: no variant |
| --- | --- | --- | --- | --- | --- | --- | --- | --- | --- | --- | --- | --- | --- | --- | --- |
|  | 264 | Familial | M | 5-8 | No | No | Possible (no details) | CLS, L | . Attention deficit hyperactivity disorder  . Learning disabilities  . Speech delay | ND | No | ND | . Scoliosis  . Pectus excavatum | No | NF1 c.3827G>A, p.Arg1276Gln  PTPN11, SOS1, SOS2, RAF1, BRAF, SHOC2, CBL, KRAS, MAP2K2, PPP1CB, RRAS, RIT1, HRAS, MAP2K1, RASA2, SPRED1: no variant |
|  | 265 | Reported sporadic | M | 5-8 | No | No | . High forehead  . Mild brachycephaly  . Downslanting palpebral fissures  . Low set ears  . Dysplastic ears with small canals | CLS | . Gross and fine motor delays  . Speech delay | ND | . Tricuspid atresia  . Hypoplastic right heart syndrome | No | No | No | NF1 c.3827G>A, p.Arg1276Gln  PTPN11, SOS1, SOS2, RAF1, BRAF, SHOC2, CBL, KRAS, MAP2K2, PPP1CB, RRAS, RIT1, HRAS, MAP2K1, RASA2, SPRED1: no variant |
|  | 266 | Reported sporadic | F | 5-8 | No | Yes | . Hypertelorism  . Ptosis  . Downslanting palpebral fissures | CLS, L | Developmental dificulties | No optic glioma | No | No | . Scoliosis  . Pectus excavatum | No | NF1 c.3827G>A, p.Arg1276Gln  PTPN11, SOS1, SOS2, RAF1, BRAF, SHOC2, CBL, KRAS, MAP2K2, PPP1CB, RRAS, RIT1, HRAS, MAP2K1, RASA2, SPRED1: no variant |
|  | 267 | Reported sporadic | ND | 5-8 | ND | ND | . Coarse face  . Low posterior hairline | CLS, L | No | ND | No | Yes | No | No | NF1 c.3827G>A, p.Arg1276Gln |
|  | 268 | Reported sporadic | M | 9-13 | No | Yes | . Hypertelorism  . Downslanting palpebral fissures | CLS | . Gross motor delay  . Speech delay  . Learning disabilty | ND | Supravalvular aortic stenosis | ND | No | . Webbed neck . Possible spinal neurofibromas | NF1 c.3827G>A, p.Arg1276Gln  PTPN11, SOS1, SOS2, RAF1, BRAF, SHOC2, CBL, KRAS, MAP2K2, PPP1CB, RRAS, RIT1, HRAS, MAP2K1, RASA2, SPRED1: no variant |
|  | 269 | Familial | F | 9-13 | No | Yes | . Hypertelorism  . Downslanting palpebral fissures | CLS, L, SNF | . Attention disorder deficit  . Autism  . Learning disability | ND | No | ND | Sphenoid wing dysplasia | No | NF1 c.3827G>A, p.Arg1276Gln  PTPN11, SOS1, SOS2, RAF1, BRAF, SHOC2, CBL, KRAS, MAP2K2, PPP1CB, RRAS, RIT1, HRAS, MAP2K1, RASA2, SPRED1: no variant |
|  | 270 | Familial | M | 9-13 | No | Yes | Possible (no details) | CLS | Developmental delay | ND | . Pulmonary stenosis  . Left ventricular hypertrophy  . Aortic stenosis and septal defect | No | Scoliosis | No | NF1 c.3827G>A, p.Arg1276Gln  PTPN11, SOS1, SOS2, RAF1, BRAF, SHOC2, CBL, KRAS, MAP2K2, PPP1CB, RRAS, RIT1, HRAS, MAP2K1, RASA2, SPRED1: no variant |
|  | 271 | ND | M | 9-13 | Yes | Yes | . Hypertelorism  . Low set ears | CLS, L | . Learning disability  . Abnormal development | ND | No | ND | Pectus excavatum | No | NF1 c.3827G>A, p.Arg1276Gln  PTPN11, SOS1, SOS2, RAF1, BRAF, SHOC2, CBL, KRAS, MAP2K2, PPP1CB, RRAS, RIT1, HRAS, MAP2K1, RASA2, SPRED1: no variant |
|  | 272 | Familial | F | 9-13 | ND | No | . Bossed forehead  . Ptosis  . Downslanting palpebral fissures | CLS, L | Learning disability | ND | No | No | No | Hypertension | NF1 c.3827G>A, p.Arg1276Gln  PTPN11, SOS1, SOS2, RAF1, BRAF, SHOC2, CBL, KRAS, MAP2K2, PPP1CB, RRAS, RIT1, HRAS, MAP2K1, RASA2, SPRED1: no variant |
|  | 273 | Reported sporadic | F | 9-13 | ND | No | Possible (no details) | CLS | No | No optic glioma (MRI) | No | Yes | No | . Incomplete extension of knee joint  . Congenital melanocytic nevus | NF1 c.3827G>A, p.Arg1276Gln |
|  | 274 | Reported sporadic | M | 9-13 | No | No | Downslanting palpebral fissures | CLS | . Learning disability  . Delayed for age | No optic glioma (MRI) | No | ND | No | No | NF1 c.3827G>A, p.Arg1276Gln |
|  | 275 | Familial | M | 9-13 | No | Yes | Midface hypoplasia | CLS, L | . Attention disorder deficit  . Learning disability | ND | No | No | . Scoliosis  . Sphenoid wing dysplasia | No | NF1 c.3827G>T, p.Arg1276Leu |
|  | 276 | Familial | M | 14-18 | No | No | Possible (no details) | CLS, L | No | No optic glioma (MRI) | No | No | No | Spinal neurofibromas | NF1 c.3826C>G, p.Arg1276Gly |

| **Table S1. Epidemiological characteristics, clinical manifestations and molecular diagnostics in series or case reports of neurofibromatosis-Noonan syndrome previously reported (continued)** | | | | | | | | | | | | | | | |  |
| --- | --- | --- | --- | --- | --- | --- | --- | --- | --- | --- | --- | --- | --- | --- | --- | --- |
| Year/Reference | Case number | NF1 family history | Sex | Age (y) | Macrocrania | Short stature | Facial Noonan phenotype | Skin manifestations | Neurological manifestations | RMI and/or CT-scan abnormalities | Cardiac manifestations | Lisch nodules | Osteoarticular manifestations | Other findings/manifestations | Molecular diagnosis | |

| 2020/Koczkowska (Hum Mutation) | 277 | Familial | F | 14-18 | No | Yes | . Hypertelorism  . Ptosis | CLS, L | Learning disability | ND | No | No | Scoliosis | No | NF1 c.3826C>G, p.Arg1276Gly |
| --- | --- | --- | --- | --- | --- | --- | --- | --- | --- | --- | --- | --- | --- | --- | --- |
|  | 278 | Reported sporadic | M | 14-18 | ND | Yes | . Ptosis | CLS, L | . Attention disorder deficit  . Learning disability | Glioma | . Pulmonary stenosis  . Aortic stenosis | No | . Scoliosis  . Pectus excavatum | . Webbed neck  . Renal artery stenosis | NF1 c.3827G>A, p.Arg1276Gln  PTPN11, SOS1, SOS2, RAF1, BRAF, SHOC2, CBL, KRAS, MAP2K2, PPP1CB, RRAS, RIT1, HRAS, MAP2K1, RASA2, SPRED1: no variant |
|  | 279 | Familial | M | 14-18 | ND | ND | . Hypertelorism  . Low set ears  . Dropping lids | CLS, L | Learning disability | Ependymoma supratentorial anaplastic | No | No | Pectus excavatum | No | NF1 c.3827G>A, p.Arg1276Gln  PTPN11, SOS1, SOS2, RAF1, BRAF, SHOC2, CBL, KRAS, MAP2K2, PPP1CB, RRAS, RIT1, HRAS, MAP2K1, RASA2, SPRED1: no variant |
|  | 280 | Proven sporadic | M | 14-18 | ND | No | . Low set ears  . Midface depression | CLS, L | No | No optic glioma (MRI) | No | No | No | No | NF1 c.3827G>A, p.Arg1276Gln  PTPN11, SOS1, SOS2, RAF1, BRAF, SHOC2, CBL, KRAS, MAP2K2, PPP1CB, RRAS, RIT1, HRAS, MAP2K1, RASA2, SPRED1: no variant |
|  | 281 | ND | F | 14-18 | No | No | . Low posterior hairline  . Midface hypoplasia | CLS, L | No | ND | No | No | Scoliosis | No | NF1 c.3827G>A, p.Arg1276Gln  PTPN11, SOS1, SOS2, RAF1, BRAF, SHOC2, CBL, KRAS, MAP2K2, PPP1CB, RRAS, RIT1, HRAS, MAP2K1, RASA2, SPRED1: no variant |
|  | 282 | Reported sporadic | M | 19-26 | Yes | No | . Low posterior hairline | CLS, PNF | Learning disability | ND | No | No | Pectus excavatum | Webbed neck | NF1 c.3827G>A, p.Arg1276Gln  PTPN11, SOS1, SOS2, RAF1, BRAF, SHOC2, CBL, KRAS, MAP2K2, PPP1CB, RRAS, RIT1, HRAS, MAP2K1, RASA2, SPRED1: no variant |
|  | 283 | Familial | F | 19-26 | No | No | . Hypertelorism  . Short palpebral fissures  . Low set ears | CLS, L | No | No optic glioma (MRI) | No | Yes | No | No | NF1 c.3827G>A, p.Arg1276Gln |
|  | 284 | Familial | M | >26 | Yes | No | . Ptosis  . Midface hypoplasia  . Low set ears  . Jaw asymmetry | CLS, L | No | ND | No | No | Scoliosis | No | NF1 c.3827G>A, p.Arg1276Gln |
|  | 285 | Familial | F | >26 | ND | ND | Possible (no details) | CLS, L | Learning disability | ND | No | ND | No | Astrocytoma | NF1 c.3827G>A, p.Arg1276Gln |
|  | 286 | Familial | F | >26 | No | Yes | Possible (no details) | CLS | No | No optic glioma (MRI) | Pulmonary stenosis | No | . Scoliosis  . Osteopenia | . Spinal neurofibromas  . Hypertension | NF1 c.3827G>A, p.Arg1276Gln |
|  | 287 | Reported sporadic | M | <2 | ND | ND | . Ptosis  . Low set ears | CLS (<6) | Abnormal development | ND | No | ND | No | No | NF1 c.4267A>G, p.Lys1423Glu  PTPN11, SOS1, SOS2, RAF1, BRAF, SHOC2, CBL, KRAS, MAP2K2, PPP1CB, RRAS, RIT1, HRAS, MAP2K1, RASA2, SPRED1: no variant |
|  | 288 | Reported sporadic | M | <2 | No | Yes | . Ptosis  . Low set ears | CLS, L | . Delayed for age  . Hypotonic | ND | Pulmonary stenosis | ND | No | Left midbrain lesion likely low-grade glioma | NF1 c.4267A>G, p.Lys1423Glu |
|  | 289 | Reported sporadic | F | <2 | No | No | . Ptosis  . Downslanting palpebral fissures  . Low set ears | CLS (<6), L | No | No optic glioma (MRI) | No | ND | No | No | NF1 c.4267A>G, p.Lys1423Glu  PTPN11, SOS1, SOS2, RAF1, BRAF, SHOC2, CBL, KRAS, MAP2K2, PPP1CB, RRAS, RIT1, HRAS, MAP2K1, RASA2, SPRED1: no variant |
|  | 290 | Proven sporadic | M | <2 | ND | ND | . Hypertelorism  . Ptosis  . Low set ears  . Low posterior hairline | CLS, L | No | No optic glioma (MRI) | No | ND | No | No | NF1 c.4267A>G, p.Lys1423Glu |
|  | 291 | ND | M | 2-4 | ND | ND | No details | CLS, L | . Speech delay  . Attention deficit hyperactivity disorder | ND | Pulmonary valve stenosis | Yes | . Pectus carinatum  . Right tibial torsion | No | NF1 c.4267A>G, p.Lys1423Glu  PTPN11, SOS1, SOS2, RAF1, BRAF, SHOC2, CBL, KRAS, MAP2K2, PPP1CB, RRAS, RIT1, HRAS, MAP2K1, RASA2, SPRED1: no variant |

| **Table S1. Epidemiological characteristics, clinical manifestations and molecular diagnostics in series or case reports of neurofibromatosis-Noonan syndrome previously reported (continued)** | | | | | | | | | | | | | | | |  |
| --- | --- | --- | --- | --- | --- | --- | --- | --- | --- | --- | --- | --- | --- | --- | --- | --- |
| Year/Reference | Case number | NF1 family history | Sex | Age (y) | Macrocrania | Short stature | Facial Noonan phenotype | Skin manifestations | Neurological manifestations | RMI and/or CT-scan abnormalities | Cardiac manifestations | Lisch nodules | Osteoarticular manifestations | Other findings/manifestations | Molecular diagnosis | |

| 2020/Koczkowska (Hum Mutation) | 292 | Reported sporadic | F | 2-4 | No | Yes | . Hypertelorism  . Midface hypoplasia  . Low set ears | CALs | . Speech delay  . Learning disability  . Abnormal development | ND | Pulmonary stenosis | ND | | No | | No | | NF1 c.4267A>G, p.Lys1423Glu  PTPN11, SOS1, SOS2, RAF1, BRAF, SHOC2, CBL, KRAS, MAP2K2, PPP1CB, RRAS, RIT1, HRAS, MAP2K1, RASA2, SPRED1: no variant | |  |
| --- | --- | --- | --- | --- | --- | --- | --- | --- | --- | --- | --- | --- | --- | --- | --- | --- | --- | --- | --- | --- |
|  | 293 | Reported sporadic | F | 2-4 | No | No | . Hypertelorism  . Ptosis | CLS, L, PNF | Attention deficit hyperactivity disorder | No optic glioma (MRI) | No | | Yes | | Sphenoid wing dysplasia | | No | | NF1 c.4267A>G, p.Lys1423Glu | |
|  | 294 | Reported sporadic | M | 2-4 | ND | ND | . Hypertelorism  . Ptosis  . Midface hypoplasia | CLS, L | No | No optic glioma (MRI) | No | | ND | | No | | No | | NF1 c.4267A>G, p.Lys1423Thr  PTPN11, SOS1, SOS2, RAF1, BRAF, SHOC2, CBL, KRAS, MAP2K2, PPP1CB, RRAS, RIT1, HRAS, MAP2K1, RASA2, SPRED1: no variant | |
|  | 295 | Proven sporadic | M | 5-8 | Yes | No | . Hypertelorism  . Midface hypoplasia  . Low set ears | CLS, L, SCNF, PNF | Speech delay | ND | No | | ND | | No | | Spinal neurofibromas | | NF1 c.4267A>G, p.Lys1423Glu  PTPN11, SOS1, SOS2, RAF1, BRAF, SHOC2, CBL, KRAS, MAP2K2, PPP1CB, RRAS, RIT1, HRAS, MAP2K1, RASA2, SPRED1: no variant | |
|  | 296 | Proven sporadic | F | 5-8 | No | Yes | . Hypertelorism  . Low set ears | CLS, L, SNF, SCNF | . Attention deficit hyperactivity disorder  . Learning disability | ND | No | | ND | | No | | No | | NF1 c.4267A>G, p.Lys1423Glu  PTPN11, SOS1, SOS2, RAF1, BRAF, SHOC2, CBL, KRAS, MAP2K2, PPP1CB, RRAS, RIT1, HRAS, MAP2K1, RASA2, SPRED1: no variant | |
|  | 297 | Reported sporadic | F | 5-8 | ND | ND | . Hypertelorism  . Low set ears | CLS | No | ND | No | | ND | | Pectus excavatum | | No | | NF1 c.4267A>G, p.Lys1423Glu  PTPN11, SOS1, SOS2, RAF1, BRAF, SHOC2, CBL, KRAS, MAP2K2, PPP1CB, RRAS, RIT1, HRAS, MAP2K1, RASA2, SPRED1: no variant | |
|  | 298 | Familial | F | 9-13 | ND | No | . Hypertelorism  . Ptosis  . Low set ears | CLS, L | . Learning disability  . Delayed for age | No optic glioma (MRI) | No | | ND | | No | | No | | NF1 c.4267A>G, p.Lys1423Gln | |
|  | 299 | Reported sporadic | M | 9-13 | ND | Yes | . Ptosis  . Downslanting palpebral fissures  . Epicanthal folds  . Low set ears  . Low posterior hairline | CLS, SNF, SCNF, PNFs | . Abnormal development  . Attention deficit hyperactivity disorder | ND | No | | ND | | Pectus excavatum | | Webbed neck | | NF1 c.4267A>G, p.Lys1423Glu  PTPN11, SOS1, SOS2, RAF1, BRAF, SHOC2, CBL, KRAS, MAP2K2, PPP1CB, RRAS, RIT1, HRAS, MAP2K1, RASA2, SPRED1: no variant | |
|  | 300 | Reported sporadic | F | 9-13 | No | Yes | Possible | CLS, L | No | Optic glioma | Pulmonary stenosis | | ND | | Scoliosis | | No | | NF1 c.4267A>G, p.Lys1423Glu | |
|  | 301 | Reported sporadic | F | 9-13 | No | No | Downslanting palpebral fissures | CLS, L | Delayed for age | Optic glioma | No | | ND | | . Scoliosis  . Pectus excavatum | | Hypothalamic glioma | | NF1 c.4267A>G, p.Lys1423Glu  SPRED1 testing negative | |
|  | 302 | Familial | M | 9-13 | Yes | No | . Hypertelorism  . Ptosis  . Low set ears | CLS, L | Gross and fine motor delays | ND | . Pulmonary stenosis  . Ventricular septal defect | | ND | | Pectus excavatum | | Webbed neck | | NF1 c.4267A>G, p.Lys1423Glu  PTPN11, SOS1, SOS2, RAF1, BRAF, SHOC2, CBL, KRAS, MAP2K2, PPP1CB, RRAS, RIT1, HRAS, MAP2K1, RASA2, SPRED1: no variant | |
|  | 303 | Familial | M | 9-13 | Yes | No | . Hypertelorism  . Ptosis  . Downslanting palpebral fissures | CLS, L | Learning disability | . No optic glioma (MRI)  . FASI | No | | ND | | No | | No | | NF1 c.4267A>G, p.Lys1423Glu | |
|  | 304 | Familial | M | 9-13 | No | No | . High and large forehead  . Ptosis  . Telecanthus  . Epicanthus (bilateral)  . Posteriorly rotated ears  . Short nose | CLS | . Attention deficit hyperactivity disorder  . Fine motor delay | No optic glioma (MRI) | No | | ND | | Pectus excavatum/carinatum | | No | | NF1 c.4267A>G, p.Lys1423Glu | |

| **Table S1. Epidemiological characteristics, clinical manifestations and molecular diagnostics in series or case reports of neurofibromatosis-Noonan syndrome previously reported (continued)** | | | | | | | | | | | | | | | |  |
| --- | --- | --- | --- | --- | --- | --- | --- | --- | --- | --- | --- | --- | --- | --- | --- | --- |
| Year/Reference | Case number | NF1 family history | Sex | Age (y) | Macrocrania | Short stature | Facial Noonan phenotype | Skin manifestations | Neurological manifestations | RMI and/or CT-scan abnormalities | Cardiac manifestations | Lisch nodules | Osteoarticular manifestations | Other findings/manifestations | Molecular diagnosis | |

| 2020/Koczkowska (Hum Mutation) | 305 | Reported sporadic | M | 14-18 | No | No | . Ptosis  . Low set ears | CLS, L | Learning disability | ND | . Bicuspid aortic valve  . Aortic regurgitation  . Aortic root dilatation  parachute deformity of mitral valve | ND | Pectus excavatum | No | NF1 c.4267A>G, p.Lys1423Glu  PTPN11, SOS1, SOS2, RAF1, BRAF, SHOC2, CBL, KRAS, MAP2K2, PPP1CB, RRAS, RIT1, HRAS, MAP2K1, RASA2, SPRED1: no variant |
| --- | --- | --- | --- | --- | --- | --- | --- | --- | --- | --- | --- | --- | --- | --- | --- |
|  | 306 | Reported sporadic | M | 14-18 | ND | No | . Hypertelorism  . Midface hypoplasia  . Low set ears | CLS, L, PNF | Attention deficit disorder | No optic glioma (MRI) | Pulmonary stenosis | ND | No | No | NF1 c.4267A>G, p.Lys1423Glu  PTPN11, SOS1, SOS2, RAF1, BRAF, SHOC2, CBL, KRAS, MAP2K2, PPP1CB, RRAS, RIT1, HRAS, MAP2K1, RASA2, SPRED1: no variant |
|  | 307 | Reported sporadic | F | 14-18 | ND | ND | . Downslanting palpebral fissures  . Broad nose  . Bulbous tip of nose  . Malocclusion of the teeth | CLS, L, PNFs | . Attention deficit disorder  . Mild mental retardation  . Seizures  . Mild tremor | No optic glioma (MRI) | Pulmonary stenosis | ND | . Lordosis  . Kyphosis | . Webbed neck  . Leg pain | NF1 c.4267A>G, p.Lys1423Glu  PTPN11, SOS1, SOS2, RAF1, BRAF, SHOC2, CBL, KRAS, MAP2K2, PPP1CB, RRAS, RIT1, HRAS, MAP2K1, RASA2, SPRED1: no variant |
|  | 308 | Reported sporadic | M | 14-18 | No | No | . Hypertelorism  . Ptosis  . Downslanting palpebral fissures  . Low set ears | CLS, L | . Attention deficit hyperactivity disorder  . Learning disability | No optic glioma (MRI) | Pulmonary stenosis | Yes | No | No | NF1 c.4267A>G, p.Lys1423Glu  PTPN11 no variant |
|  | 309 | Proven sporadic | M | 19-26 | No | No | Possible  Abnormal ear shape | CLS | No | ND | No | ND | No | . Spinal neurofibromas  . Pilocytic astrocytoma  . Non-ossifying fibromas | NF1 c.4267A>G, p.Lys1423Glu  SPRED1 testing negative |
|  | 310 | Reported sporadic | M | >26 | Yes | No | . Hypertelorism  . Low set ears | CLS, L, SNF | No | ND | No | ND | No | . Non-ossifying fibromas  . Multiple giant cell tumors (jaw)  . History of rhabdomyosarcoma  . Bone cysts | NF1 c.4267A>G, p.Lys1423Glu  PTPN11, SOS1, SOS2, RAF1, BRAF, SHOC2, CBL, KRAS, MAP2K2, PPP1CB, RRAS, RIT1, HRAS, MAP2K1, RASA2, SPRED1: no variant |
|  | 311 | ND | M | >26 | Yes | No | . Downslanting palpebral fissures  . Low set ears | CLS, L, SNF, SCNF | No | . Brain stem glioma  . No optic glioma (MRI) | No | ND | No | Spinal neurofibromas | NF1 c.4267A>G, p.Lys1423Glu  PTPN11, SOS1, SOS2, RAF1, BRAF, SHOC2, CBL, KRAS, MAP2K2, PPP1CB, RRAS, RIT1, HRAS, MAP2K1, RASA2, SPRED1: no variant |
|  | 312 | Reported sporadic | M | >26 | No | No | Ptosis | CLS, L, SNF, SCNF | . Attention deficit hyperactivity disorder  . Learning disability | No optic glioma (MRI) | Pulmonary stenosis | Yes | No | No | NF1 c.4267A>G, p.Lys1423Glu  PTPN11 no variant |
|  | 313 | Reported sporadic | M | >26 | ND | No | . Hypertelorism  . Ptosis | CLS, L, SNF, SCNF | No | . No optic glioma (MRI)  . Meningioma  . Motor polyneuropathy with myelin damage | No | Yes | No | Colon adenocarcinoma | NF1 c.4267A>G, p.Lys1423Glu |
| 2020/Zhang (BMC Pediatrics) | 314 | Sporadic | M | 1.25 | No | Yes | No details | CLS | No | RMI: FASIs | Atrial septal defect | ND | Pectus excavatum | Cryptochordism | NF1 deletion (exon 1-58)  PTPN11, SOS1, RAF1, BRAF, SHOC2, KRAS, HRAS, MAP2K2, PPP1CB, RRAS, MAP2K1, SPRED1: no variant |
| 2020/Isik (J Clin Res Pediatr Endocrinol) | 315 | Familial | F | 7 | Yes | Yes | . Broad forehead  . Hypertelorism  . Sparse eyebrows  . Depressed nasal bridge  . Low set ears  . Deeply grooved philtrum | CLS, L, PNF | No | RMI: hamartoma | Echocardiography normal | No | . Scoliosis  . Pectus excavatum  . Kyphosis | . Short and webbed neck  . Spina bifida  . Filum terminale lipoma | NF1 c.3052_3056delTTAGT, p.Leu1018*  PTPN11 no variant |

| **Table S1. Epidemiological characteristics, clinical manifestations and molecular diagnostics in series or case reports of neurofibromatosis-Noonan syndrome previously reported (end)** | | | | | | | | | | | | | | | |  |
| --- | --- | --- | --- | --- | --- | --- | --- | --- | --- | --- | --- | --- | --- | --- | --- | --- |
| Year/Reference | Case number | NF1 family history | Sex | Age (y) | Macrocrania | Short stature | Facial Noonan phenotype | Skin manifestations | Neurological manifestations | RMI and/or CT-scan abnormalities | Cardiac manifestations | Lisch nodules | Osteoarticular manifestations | Other findings/manifestations | Molecular diagnosis | |

| 2021/D’Amico (Clin Gen) | 316 | Familial | F | 10 | ND | Yes | . Ptosis  . Downslanting palpebral fissures  . High palate | CLS, L, PNFs (2) | . Developmental delay  . Learning problems  . Intellectual disability | MRI: pilocytic astrocytomas in cerebellar hemispheres  . Optic glioma . FASIs | Pulmonary stenosis | Yes | . Scoliosis  . Pectus excavatum | Enlarged spleen | NF1 c.1964del, p.Gly655Glufs*33  PTPN11 c.209A>G, p.Lys70Arg |
| --- | --- | --- | --- | --- | --- | --- | --- | --- | --- | --- | --- | --- | --- | --- | --- |
|  | 317 | Familial | M | 12 | ND | Yes | . Downslanting palpebral fissures  . Low set ears | CLS, L | Developmental delay | MRI: pilocytic astrocytomas in cerebellar hemispheres  . FASIs | Pulmonary stenosis | Yes | . Scoliosis  . Pectus excavatum | Enlarged spleen | NF1 c.1964del, p.Gly655Glufs*33  PTPN11 c.209A>G, p.Lys70Arg |
|  | 318 | Sporadic | F | 11 | Yes | Yes | . Triangular face  . Broad forehead | CLS, L | Learning problems | MRI: optic glioma, pilocytic astrocytomas  . FASIs | Atrial septal defect | Yes | . Scoliosis  . Pectus carinatum | . Enlarged spleen  . Bilateral blue sclera | NF1 c.7778delA, p.Lys2593Argfs*10  PTPN11 c.794G>A, p.Arg265Gln |
| 2022/Qin (J Clin Res Pediatr Endocrinol) | 319 | Sporadic | M | 12 | Yes (relative) | No | . Triangular face.  . Broad forehead  . Low-set and posteriorly rotated ears  . Sparse eyebrows | CLS, L | No | Abnormal nodular signals in bilateral basal ganglia-thalamus region | No | Yes | Scoliosis | . Spinal NF (?)  . Growth hormone deficiency | NF1 c.6189C>G, p.tyr2063*  Whole-exome sequencing: PTPN11 no variant |
| 2023/Dalili (Human Genomics) | 320 | Sporadic | F | 7 | No | Yes | . Broad forehead.  . Hypertelorism  . Epicanthal folds  . Low set ears | CLS | . Developmental delay  . Learning disability  . Inadequate speech skill | Brain RMI normal | . Pulmonary stenosis  . Tricuspid regurgitation | No | No | . Webbed neck  . Dark brown macules (tongue, buccal surface and sclera) | NF1 c.4375-4377delGAA, p.Glu1459del (Whole-exome sequencing) |
| 2023/Mohapatra and Samantaray (Cureus) | 321 | ND | F | 16 | ND | Yes | . Hypertelorism  . Telecanthus  . Pointed pinna | CLS | No | MRI: moderate communicating hydrocephalus with periventricular interstitial edema | No  ECG normal | No | No | Primary amenorrhoea | NF1 c.5592_5596delTTTAA (p.Asn1864Lysfs*26) (Whole genome sequencing) |

**References**

1. Allanson JE et al. [Noonan phenotype associated with neurofibromatosis.](https://pubmed.ncbi.nlm.nih.gov/2411134/) Am J Med Genet. 1985; 21:457-62.
2. Abuelo DN, Meryash DL. [Neurofibromatosis with fully expressed Noonan syndrome.](https://pubmed.ncbi.nlm.nih.gov/3135755/) Am J Med Genet. 1988; 29:937-41.
3. Bahuau M et al. [Exclusion of allelism of Noonan syndrome and neurofibromatosis-type 1 in a large family with Noonan syndrome-neurofibromatosis association.](https://pubmed.ncbi.nlm.nih.gov/8985499/) Am J Med Genet. 1996; 66:347-55.
4. Bahuau M et al. [Novel recurrent nonsense mutation causing neurofibromatosis type 1 (NF1) in a family segregating both NF1 and Noonan syndrome.](https://pubmed.ncbi.nlm.nih.gov/9475595/) Am J Med Genet. 1998; 75:265-72.
5. Baquedano Lobera I et al. [The Arg1038Gly missense variant in the NF1 gene causes a mild phenotype without neurofibromas.](https://pubmed.ncbi.nlm.nih.gov/30843352/) BMC Pediatr. 2019; 19:92.
6. Baralle D et al. [Different mutations in the NF1 gene are associated with Neurofibromatosis-Noonan syndrome (NFNS).](https://pubmed.ncbi.nlm.nih.gov/12707950/) Am J Med Genet A. 2003; 119A:1-8.
7. Ben-Shachar S et al. [Increased rate of missense/in-frame mutations in individuals with NF1-related pulmonary stenosis: a novel genotype-phenotype correlation.](https://pubmed.ncbi.nlm.nih.gov/23047742/) Eur J Hum Genet. 2013; 21:535-9.
8. Bertola DR et al. [Neurofibromatosis-Noonan syndrome: molecular evidence of the concurrence of both disorders in a patient.](https://pubmed.ncbi.nlm.nih.gov/15948193/) Am J Med Genet A. 2005; 136:242-5.
9. [Borochowitz Z et al. The neurofibromatosis-Noonan syndrome: genetic heterogeneity versus clinical variability. Case report and review of the literature.](https://pubmed.ncbi.nlm.nih.gov/2518512/) Neurofibromatosis. 1989; 2:309-14.
10. Buehning L, Curry CJ. [Neurofibromatosis-Noonan syndrome.](https://pubmed.ncbi.nlm.nih.gov/7501563/) Pediatr Dermatol. 1995; 12:267-71.
11. Castle B et al. [Evaluation of genotype-phenotype correlations in neurofibromatosis type 1.](https://pubmed.ncbi.nlm.nih.gov/14569132/) J Med Genet. 2003; 40:e109.
12. Colley A et al. [Neurofibromatosis/Noonan phenotype: a variable feature of type 1 neurofibromatosis.](https://pubmed.ncbi.nlm.nih.gov/8740913/) Clin Genet. 1996; 49:59-64.
13. Croonen EA et al. [Patient with a neurofibromatosis type 1 mutation but a clinical diagnosis of Noonan syndrome.](https://pubmed.ncbi.nlm.nih.gov/22664660/) Clin Dysmorphol. 2012; 21:212-4.
14. D'Amico A et al. [Clinical variability of neurofibromatosis 1: A modifying role of cooccurring PTPN11 variants and atypical brain MRI findings.](https://pubmed.ncbi.nlm.nih.gov/34346503/) Clin Genet. 2021; 100:563-72.
15. Dalili S et al. [Neurofibromatosis-Noonan syndrome and growth deficiency in an Iranian girl due to a pathogenic variant in NF1 gene.](https://pubmed.ncbi.nlm.nih.gov/36803953/) Hum Genomics. 2023; 17:12.
16. De Luca A et al. [NF1 gene mutations represent the major molecular event underlying neurofibromatosis-Noonan syndrome.](https://pubmed.ncbi.nlm.nih.gov/16380919/) Am J Hum Genet. 2005; 77:1092-101.
17. Ekvall S et al. [Novel association of neurofibromatosis type 1-causing mutations in families with neurofibromatosis-Noonan syndrome.](https://pubmed.ncbi.nlm.nih.gov/24357598/) Am J Med Genet A. 2014; 164A:579-87.
18. Hüffmeier U et al. [A variable combination of features of Noonan syndrome and neurofibromatosis type I are caused by mutations in the NF1 gene.](https://pubmed.ncbi.nlm.nih.gov/17103458/) Am J Med Genet A. 2006; 140:2749-56.
19. Işık E et al. [A Neurofibromatosis Noonan Syndrome Patient Presenting with Abnormal External Genitalia.](https://pubmed.ncbi.nlm.nih.gov/31088041/) J Clin Res Pediatr Endocrinol. 2020; 12:113-6.
20. Kayes LM et al. [Deletions spanning the neurofibromatosis 1 gene: identification and phenotype of five patients.](https://pubmed.ncbi.nlm.nih.gov/8116612/) Am J Hum Genet. 1994; 54:424-36.
21. Kaplan P, Rosenblatt B. [A distinctive facial appearance in neurofibromatosis von Recklinghausen.](https://pubmed.ncbi.nlm.nih.gov/3927724/) Am J Med Genet. 1985; 21:463-70.
22. Koczkowska M et al. [Genotype-Phenotype Correlation in NF1: Evidence for a More Severe Phenotype Associated with Missense Mutations Affecting NF1 Codons 844-848.](https://pubmed.ncbi.nlm.nih.gov/29290338/) Am J Hum Genet. 2018; 102:69-87.
23. Koczkowska M et al. [Expanding the clinical phenotype of individuals with a 3-bp in-frame deletion of the NF1 gene (c.2970_2972del): an update of genotype-phenotype correlation.](https://pubmed.ncbi.nlm.nih.gov/30190611/) Genet Med. 2019; 21:867-76.
24. Koczkowska M et al. [Clinical spectrum of individuals with pathogenic NF1 missense variants affecting p.Met1149, p.Arg1276, and p.Lys1423: genotype-phenotype study in neurofibromatosis type 1.](https://pubmed.ncbi.nlm.nih.gov/31595648/) Hum Mutat. 2020; 41:299-315.
25. Meinecke P. [Evidence that the "neurofibromatosis-Noonan syndrome" is a variant of von Recklinghausen neurofibromatosis.](https://pubmed.ncbi.nlm.nih.gov/3105316/) Am J Med Genet. 1987; 26:741-5.
26. Mendez HM. [The neurofibromatosis-Noonan syndrome.](https://pubmed.ncbi.nlm.nih.gov/3927725/) Am J Med Genet. 1985; 21:471-6.
27. [Mohapatra I, Samantaray SR. Neurofibromatosis-Noonan Syndrome With Primary Amenorrhoea: A Case Report.](https://pubmed.ncbi.nlm.nih.gov/37602003/) Cureus. 2023; 15:e42098.
28. [Nyström AM et al. Noonan syndrome and neurofibromatosis type I in a family with a novel mutation in NF1.](https://pubmed.ncbi.nlm.nih.gov/19845691/) Clin Genet. 2009; 76:524-34.
29. Nyström AM et al. [A severe form of Noonan syndrome and autosomal dominant café-au-lait spots - evidence for different genetic origins.](https://pubmed.ncbi.nlm.nih.gov/19120036/) Acta Paediatr. 2009; 98:693-8.
30. [Opitz JM, Weaver DD. The neurofibromatosis-Noonan syndrome.](https://pubmed.ncbi.nlm.nih.gov/3927726/) Am J Med Genet. 1985; 21:477-90.
31. [Pinna V et al. p.Arg1809Cys substitution in neurofibromin is associated with a distinctive NF1 phenotype without neurofibromas.](https://pubmed.ncbi.nlm.nih.gov/25370043/) Eur J Hum Genet. 2015; 23:1068-71.
32. Pinna V et al. [Prevalence, Type, and Molecular Spectrum of NF1 Mutations in Patients with Neurofibromatosis Type 1 and Congenital Heart Disease.](https://pubmed.ncbi.nlm.nih.gov/31487937/) Genes (Basel). 2019; 10:675.
33. Prada CE et al. [Lethal presentation of neurofibromatosis and Noonan syndrome.](https://pubmed.ncbi.nlm.nih.gov/21567923/) Am J Med Genet A. 2011; 155A:1360-6.
34. Qin S et al. [A Novel Heterozygous NF1 Variant in a Neurofibromatosis-Noonan Syndrome Patient with Growth Hormone Deficiency: A Case Report.](https://pubmed.ncbi.nlm.nih.gov/35633639/) J Clin Res Pediatr Endocrinol. 2023; 15:438-43.
35. Quattrin T et al. [Vertical transmission of the neurofibromatosis/Noonan syndrome.](https://pubmed.ncbi.nlm.nih.gov/3105315/) Am J Med Genet. 1987; 26:645-9.
36. Reig I et al. [Neurofibromatosis-Noonan syndrome: case report and clinicopathogenic review of the Neurofibromatosis-Noonan syndrome and RAS-MAPK pathway.](https://pubmed.ncbi.nlm.nih.gov/21549079/) Dermatol Online J. 2011; 17:4.
37. Rojnueangnit K et al. [High Incidence of Noonan Syndrome Features Including Short Stature and Pulmonic Stenosis in Patients carrying NF1 Missense Mutations Affecting p.Arg1809: Genotype-Phenotype Correlation.](https://pubmed.ncbi.nlm.nih.gov/26178382/) Hum Mutat. 2015; 36:1052-63.
38. Santoro C et al. [Arg(1809) substitution in neurofibromin: further evidence of a genotype-phenotype correlation in neurofibromatosis type 1.](https://pubmed.ncbi.nlm.nih.gov/25966637/) Eur J Hum Genet. 2015; 23:1460-1.
39. Saul RA. [Noonan syndrome in a patient with hyperplasia of the myenteric plexuses and neurofibromatosis.](https://pubmed.ncbi.nlm.nih.gov/3927727/) Am J Med Genet. 1985; 21:491-2.
40. Shuper A et al. [Noonan's syndrome and neurofibromatosis.](https://pubmed.ncbi.nlm.nih.gov/3103548/) Arch Dis Child. 1987; 62:196-8.
41. Stern HJ et al. [Clinical variability of type 1 neurofibromatosis: is there a neurofibromatosis-Noonan syndrome?](https://pubmed.ncbi.nlm.nih.gov/1348094/) J Med Genet. 1992; 29:184-7.
42. Stevenson DA et al. [Clinical and molecular aspects of an informative family with neurofibromatosis type 1 and Noonan phenotype.](https://pubmed.ncbi.nlm.nih.gov/16542390/) Clin Genet. 2006; 69:246-53.
43. Tassabehji M et al. [Tandem duplication within a neurofibromatosis type 1 (NF1) gene exon in a family with features of Watson syndrome and Noonan syndrome.](https://pubmed.ncbi.nlm.nih.gov/8317503/) Am J Hum Genet. 1993; 53:90-5.
44. Thiel C et al. [Independent NF1 and PTPN11 mutations in a family with neurofibromatosis-Noonan syndrome.](https://pubmed.ncbi.nlm.nih.gov/19449407/) Am J Med Genet A. 2009; 149A:1263-7.
45. Trevisson E et al. [Rasopathies case report : concurrence of two pathogenic variations de novo in NF1 and KRAS genes in a patient.](https://pubmed.ncbi.nlm.nih.gov/30953504/) Mol Genet Genomic Med. 2019; 7:e616.
46. Upadhyaya M et al. [An absence of cutaneous neurofibromas associated with a 3-bp inframe deletion in exon 17 of the NF1 gene (c.2970-2972 delAAT): evidence of a clinically significant NF1 genotype-phenotype correlation.](https://pubmed.ncbi.nlm.nih.gov/17160901/) Am J Hum Genet. 2007; 80:140-51.
47. Vurallı D et al. [Growth Hormone Deficiency in a Child with Neurofibromatosis-Noonan Syndrome.](https://pubmed.ncbi.nlm.nih.gov/26758488/) J Clin Res Pediatr Endocrinol. 2016; 8:96-100.
48. Wu R et al. [Neurofibromatosis type I gene mutation in a patient with features of LEOPARD syndrome.](https://pubmed.ncbi.nlm.nih.gov/8807336/) Hum Mutat. 1996; 8:51-6.
49. Yapijakis C et al. [Is Neurofibromatosis Type 1-Noonan Syndrome a Phenotypic Result of Combined Genetic and Epigenetic Factors?](https://pubmed.ncbi.nlm.nih.gov/27107091/) In Vivo. 2016; 30:315-20.
50. Yazdizadeh M et al. [A case of neurofibromatosis-Noonan syndrome with a central giant cell granuloma.](https://pubmed.ncbi.nlm.nih.gov/15356469/) Oral Surg Oral Med Oral Pathol Oral Radiol Endod. 2004; 98:316-20.
51. Yimenicioğlu S et al. [A new nonsense mutation in the NF1 gene with neurofibromatosis-Noonan syndrome phenotype.](https://pubmed.ncbi.nlm.nih.gov/22965773/) Childs Nerv Syst. 2012; 28:2181-3.
52. Zhang Z et al. [Chinese patient with neurofibromatosis-Noonan syndrome caused by novel heterozygous NF1 exons 1-58 deletion: a case report.](https://pubmed.ncbi.nlm.nih.gov/32357851/) BMC Pediatr. 2020; 20:190.
