# Supplementary material for: Neurofibromatosis-Noonan syndrome: a prospective monocentric study of 26 patients and literature review
Source: Orphanet J Rare Dis. 2025 Apr 27;20:201. doi: 10.1186/s13023-025-03706-3 (PMC12036184; doi:10.1186/s13023-025-03706-3)
Supplement: Supplementary file 2 — Supplementary Material 2 [file 13023_2025_3706_MOESM2_ESM.docx]

| **Table S2. Details of baseline, facial Noonan phenotype, clinical manifestations and molecular characteristics of the 26 patients with NF1 pathogenic variants** | | | | | | | | | | | | | | | | |
| --- | --- | --- | --- | --- | --- | --- | --- | --- | --- | --- | --- | --- | --- | --- | --- | --- |
| Case number | NF1 family history | Sex | Age (year) | Noonan phenotype | Macrocrania | Short stature | Facial Noonan phenotype | Clinical manifestations | | | | | | Cerebral CT-scan and/or MRI | *NF1* pathogenic variant | Pathogenic variant other RASopathies** |
|  |  |  |  |  |  |  |  | Skin* | Neurological | Cardiovascular | Ocular | Osteoarticular | Other |  |  |  |
| 1 | Sporadic | M | 6 | Suggestive | No | No | . Coarse face  . Ptosis (bilateral)  . Down-slanting palpebral fissures  . Epicanthal folds  . Bulbous nasal tip  . Wide prominent philtrum  . Micrognathia | L, CLS, SNF (>2), PNF | . Delayed psychomotor development  . Delayed language development | . Arterial hypertension  . Pulmonary arterial hypertension  . Moya Moya syndrome  . Aortic stenosis (TTE)  . Arterial renal stenosis  . ECG: incomplete branch block right ventricular hypertrophy | No Lisch nodule | No | No | . Ischemic strokes  . No optic glioma  . FASI (thalamic) | Deletion (type 1) | No |
| 2 | Familial | F | 45 | Suggestive | No | No | . Down-slanting palpebral fissures  . Low set posteriorly angulated ears  . Prominent nasolabial folds | L, CLS | No | No (heart auscultation) | No Lisch nodule | . Scoliosis  . Pectus excavatum | No | Normal | c.4267A>G, p.Lys1423Glu | No |
| 3 | Sporadic | M | 8 | Suggestive | Yes (+3 SD) | No | . Hypoplasia of the midface  . Triangular face  . Low set posteriorly angulated ears  . High and broad nasal bridge  . Wide and prominent philtrum  . Wide peaks to vermillion border of the upper lip  . Low posterior hairline | L, CLS, SNF (>2), PNFs (x2) | Attention deficit hyperactivity disorder | No (TTE and ECG normal) | No Lisch nodule | Pectus excavatum | No | . FASI (cerebellum, brain stem)  . No optic glioma | c.7267dup, p.Thr2423Asnfs*4 | No |
| 4 | Sporadic | M | 12 | Suggestive | Yes (+2.5 SD) | Yes (<2 SD) | . Facial asymmetry  . Down-slanting palpebral fissures  . Epicanthal folds  . Low set posteriorly angulated ears  . Midface hypoplasia  . Wide and prominent philtrum | L, CLS, SNF (>2) | Delayed psychomotor development | No (TTE and ECG normal) | No Lisch nodule | Pectus excavatum | Precocious puberty | . Optic glioma  . Astrocytoma (cerebellum) | c.910C>T, p.Arg304* | No |
| 5 | Sporadic | F | 9 | Typical | Yes (+2.5 SD) | Yes (< 2 SD) | . Prominent and high forehead  . Ptosis  . Hypertelorism  . Down-slanting palpebral fissures  . Low set posteriorly angulated ears | L, CLS, PNFs (x3) | Attention deficit hyperactivity disorder | No (TTE and ECG normal) | . Coloboma (bilateral)  . No Lisch nodule | No | No | . Optic glioma  . FASI (cerebellum, globus pallidus, sylvian aqueduct) | c.499_502del, p.Cys167Glnfs*10 | No |
| 6 | Familial | M | 8 | Typical | Yes (+2 SD) | No | . Facial asymmetry  . Ptosis  . Hypertelorism  . Down-slanting palpebral fissures  . Eversion of the lateral eyelid  . Epicanthal folds  . Low set posteriorly angulated ears  . Wide and prominent philtrum  . Micrognathia | L, CLS, PNFs (x2) | No | . Pulmonary stenosis (supravalvular; TTE)  . ECG normal | ND | Pectus excavatum | No | . Spheno-orbital dysplasia  . Arachnoid cysts  . FASI (cerebellum, globus pallidus)  . No optic glioma | c.3827G>A, p.Arg1276Gln | No |
| 7 | Sporadic | M | 9 | Typical | Yes (+2 SD) | No | . Prominent and high forehead  . Hypertelorism  . Hypoplasia of the midface  . Down-slanting palpebral fissures  . Epicanthal folds  . High and broad nasal bridge  . Bulbous nasal tip | L, CLS, SNF (>2), SCNF | Attention deficit hyperactivity disorder | No (TTE and ECG normal) | No Lisch nodule | . Scoliosis  . Pectus excavatum | Malignant peripheral nerve sheath tumor (leg) | . FASI (cerebellum, globus pallidus, brain stem)  . No optic glioma | c.61-2A>G p.Leu21Aspfs*18 | No |
| 8 | Sporadic | M | 8 | Suggestive | No | No | . Facial asymmetry  . Prominent and high forehead | L, CLS, SCNF (1) | No | No (TTE and ECG normal) | No Lisch nodule | No | No | ND | c.5005_5006del, p.(Thr1669Glnfs*4) | No |
| Abbreviations: F, female; M, male; L, lentigines; CLS, café-au-lait spots; CT, computed tomography; ECG, electrocardiogram; FASI, focal areas of signal intensity; ND, not done; PNF, plexiform neurofibroma; RMI, resonance magnetic imaging; SCNF, subcutaneous neurofibromas; SD, standard deviation; SNF, superficial neurofibromas; TTE, transthoracic echocardiography.  * Lentigines (axillary and/or inguinal) and café-au-lait spots (over 5 mm in diameter in prepubertal individuals and over 15 mm in greatest diameter in postpubertal individuals) were consistently more than 2 and 5 in number, respectively.  **Including pathogenic variants of the following genes, involved in the RAS-MAPkinases pathway: *PTPN11*, *SOS1*, *SOS2*, *SHOC2*, *CBL*, *HRAS*, *NRAS*, *KRAS*, *RIT1*, *RRAS*, *RRAS2*, *BRAF*, *RAF1*, *MAP2K1*, *MAP2K2*, *SPRED1*, *SPRED2*, *NF1*, *PPP1CB*, and *LZTR1*. | | | | | | | | | | | | | | | | |

| **Table S2. Details of baseline, facial Noonan phenotype, clinical manifestations and molecular characteristics of the 26 patients with NF1 pathogenic variants (continued)** | | | | | | | | | | | | | | | | |
| --- | --- | --- | --- | --- | --- | --- | --- | --- | --- | --- | --- | --- | --- | --- | --- | --- |
| Case number | NF1 family history | Sex | Age (year) | Noonan phenotype | Macrocrania | Short stature | Facial Noonan phenotype | Clinical manifestations | | | | | | Cerebral CT-scan and/or MRI | NF1 pathogenic variant | Pathogenic variant other RASopathies* |
|  |  |  |  |  |  |  |  | Skin | Neurological | Cardiovascular | Ocular | Osteoarticular | Other |  |  |  |
| 9 | Familial | F | 27 | Suggestive | No | No | . Prominent and high forehead  . High anterior hairline  . Hypertelorism  . Low set posteriorly angulated ears  . Bulbous nasal tip | L, CLS, SCNF, PNFs (2) | No | No (TTE and ECG normal) | ND | No | Subcutaeous venous malformation (right temporal) | . Spheno-maxillary dysplasia  . FASI (frontal sub-cortical)  . No optic glioma | c.889-2A>G, p.Lys297_Lys354del | No |
| 10 | Familial | M | 47 | Suggestive | No | No | . High anterior hairline  . Low set posteriorly angulated ears  . Prominent nasolabial folds | L, CLS, SNF (>2) | No | No (TTE and ECG normal) | ND | No | Ischemic cardiopathy (smoking habit) | ND | c.4735A>T, p.(Lys1579*) | ND |
| 11 | Sporadic | M | 10 | Typical | Yes (SD ND) | No | . Prominent and high forehead  . Frontal bossing  . Hypertelorism  . Triangular face  . Low set posteriorly angulated ears  . Small and pointed chin | L, CLS, SNF (>2), PNFs (2) | Concentration disorders | No (TTE and ECG normal) | Lisch nodules | No | No | . FASI (cerebellum, thalamic, lenticular nucleus)  . No optic glioma | c.4318dup, p.(Met1440Asnfs*6) | No |
| 12 | Familial | M | 48 | Suggestive | Yes (+2 SD) | No | . High anterior hairline  . Down-slanting palpebral fissures  . Prominent nasal folds  . Bulbous nasal tip  . Wide peaks to vermillion border of the upper lip | L, CLS, SNF (>2), SCNF, PNF | No | . Mitral valvular prolapse (minimal) (TTE)  . Incomplete branch block right (ECG) | Lisch nodules | Pectus excavatum | Arterial hypertension | . FASI (thalamic)  . No optic glioma | c.7528C>T, p.Gln2510* | No |
| 13 | Sporadic | M | 10 | Suggestive | Yes (+2 SD) | No | . Prominent and high forehead  . Frontal bossing  . Triangular face  . Midface hypoplasia  . High and broad nasal bridge  . Wide and prominent philtrum  . Small and pointed chin | L, CLS | Learning disability | No (TTE and ECG normal) | Lisch nodules | . Scoliosis (severe)  . Pectus excavatum | Spinal neurofibromas | Optic glioma | c.1260+1604A>G, p.Ser421Leufs*4 | No |
| 14 | Sporadic | M | 7 | Suggestive | No | No | . Ptosis  . Down-slanting palpebral fissures | L, CLS | . Attention deficit hyperactivity disorder  . Learning disability  . Intellectual disability | No (TTE and ECG normal) | No Lisch nodule | Pectus excavatum | No | Normal | c.4180A>C, p.(Asn1394His) | No |
| 15 | Familial | M | 3.5 | Typical | No | Yes (< 2 SD) | . Frontal bossing  . Hypertelorism  . Down-slanting palpebral fissures  . Low set posteriorly angulated ears | CLS | No | No (TTE and ECG normal) | No Lisch nodules | Pectus excavatum | No | ND | c.3832A>T, p.(Asn1278Tyr) | No |
| 16 | Sporadic | M | 10 | Suggestive | No | No | . Facial asymmetry  . Epicanthus  . Down-slanting palpebral fissures  . Thickened upper helix  . Micrognathia | L, CLS, PNF | Learning disability | No (TTE and ECG normal) | Lisch nodules | Pectus excavatum (minimal) | No | ND | c.3827G>A, p.Arg1276Gln | No |
| 17 | Sporadic | F | 8 | Suggestive | Yes (+2.8 SD) | No | . Triangular face  . Hypertelorism  . Down-slanting palpebral fissures  . Epicanthus  . Wide and prominent philtrum  . Micrognathia | L, CLS, PNF | Learning disability | No (TTE and ECG normal) | No Lisch nodules | . Pectus excavatum  . Kyphosis (dorsal) | No | . Optic glioma  . FASI (cerebellum, brain stem, basal ganglia) | c.5206-1G>A, p? | No |
| Abbreviations: F, female; M, male; L, lentigines; CLS, café-au-lait spots; CT, computed tomography; ECG, electrocardiogram; FASI, focal areas of signal intensity; ND, not done; PNF, plexiform neurofibroma; RMI, resonance magnetic imaging; SCNF, subcutaneous neurofibromas; SD, standard deviation; SNF, superficial neurofibromas; TTE, transthoracic echocardiography.  * Lentigines (axillary and/or inguinal) and café-au-lait spots (over 5 mm in diameter in prepubertal individuals and over 15 mm in greatest diameter in postpubertal individuals) were consistently more than 2 and 5 in number, respectively.  **Including pathogenic variants of the following genes, involved in the RAS-MAPkinases pathway: *PTPN11*, *SOS1*, *SOS2*, *SHOC2*, *CBL*, *HRAS*, *NRAS*, *KRAS*, *RIT1*, *RRAS*, *RRAS2*, *BRAF*, *RAF1*, *MAP2K1*, *MAP2K2*, *SPRED1*, *SPRED2*, *NF1*, *PPP1CB*, and *LZTR1*. | | | | | | | | | | | | | | | | |
|  | | | | | | | | | | | | | | | | |

| **Table S2. Details of baseline, facial Noonan phenotype, clinical manifestations and molecular characteristics of the 26 patients with NF1 pathogenic variants (end)** | | | | | | | | | | | | | | | | |
| --- | --- | --- | --- | --- | --- | --- | --- | --- | --- | --- | --- | --- | --- | --- | --- | --- |
| Case number | NF1 family history | Sex | Age (year) | Noonan phenotype | Macrocrania | Short stature | Facial Noonan phenotype | Clinical manifestations | | | | | | Cerebral CT-scan and/or MRI | NF1 pathogenic variant | Pathogenic variant other RASopathies* |
|  |  |  |  |  |  |  |  | Skin | Neurological | Cardiovascular | Ocular | Osteoarticular | Other |  |  |  |
| 18 | Familial | M | 9 | Typical | No | No | . Prominent and high forehead  . Ptosis  . Hypertelorism  . High and broad nasal bridge  . Hypoplasia of the midface  . Small and pointed chin | L, CLS | . Attention deficit hyperactivity disorder  . Learning disability  . Dysgraphia | No (TTE and ECG normal) | No Lisch nodules | Pectus excavatum | No | Normal | c.5425C>T, p.Arg1809Cys | No |
| 19 | Familial | M (father of 18) | 45 | Suggestive | No | No | . Frontal bossing  . Hypertelorism  . Hypoplasia of the midface  . Low set posteriorly angulated ears  . Prominent nasolabial folds | L, CLS | No | No (heart auscultation) | No Lisch nodules | Pectus excavatum | No | ND | c.5425C>T, p.Arg1809Cys | ND |
| 20 | Familial | M (brother of 18) | 12 | Typical | No | Yes (< 2 SD) | . Ptosis  . Hypertelorism  . Down slanting palpebral fissures  . Low set posteriorly angulated ears  . Wide and prominent philtrum  . Micrognathia | L, CLS | . Attention deficit hyperactivity disorder  . Learning disability | No (TTE and ECG normal) | No Lisch nodules | Pectus excavatum | Scaphocephaly (craniostenosis history) | Optic glioma (bilateral) | c.5425C>T, p.Arg1809Cys | No |
| 21 | Sporadic | M | 19 | Suggestive | Yes (+2 SD) | No | . Flat occiput  . Down slanting palpebral fissures  . Eversion of the lateral eyelid  . Low set posteriorly angulated ears  . Wide and prominent philtrum | L, CLS, SNF (>2), SCNF | . Attention deficit hyperactivity disorder  . Learning disability  . Mild mental retardation  . Psychiatric auto-aggressive behaviour | . Pulmonary stenosis (TTE)  . ECG normal | . Nystagmus  . No Lisch nodules | . Pectus excavatum  . Scoliosis  . Flat feet | No | . Optic glioma  . FASI (cerebellum, brain stem) | Deletion (type 1) | No |
| 22 | Familial | M | 14 | Typical | Yes (+2.4 SD) | No | . Prominent and high forehead  . Ptosis  . Triangular face  . Low set posteriorly angulated ears  . Wide and prominent philtrum | L, CLS | . Attention deficit hyperactivity disorder  . Learning disability | No (TTE and ECG normal) | ND | Kyphosis (lumbar) | Dystonia (DYT1 pathogen variant) | Normal | c.5425C>T, p.Arg1809Cys | No |
| 23 | Familial | F | 11 | Suggestive | No | No | . Low set posteriorly angulated ears  . High and broad nasal bridge  . Wide and prominent philtrum  . Micrognathia | L, CLS | No | No (heart auscultation) | No Lisch nodules | No | No | FASI (cerebellum) | c.5425C>T, p.Arg1809Cys | No |
| 24 | Familial | F | 1 | Suggestive | No | Yes (< 2 SD) | . Hypertelorism  . Low set posteriorly angulated ears  . High and broad nasal bridge  . Triangular face  . Micrognathia | L, CLS | No | No (TTE and ECG normal) | No Lisch nodules | Pectus excavatum | No | ND | c.889-2A>G, p.Lys297_Lys354del | No |
| 25 | Sporadic | F | 18 | Suggestive | No | No | . Coarse face  . Ptosis  . Hypertelorism  . Bulbous nasal tip  . Wide and prominent philtrum  . Micrognathia | L, CLS, SNF (>2), PNFs (x2) | . Attention deficit hyperactivity disorder  . Learning disability  . Mild mental retardation | No (TTE and ECG normal) | Lisch nodules | Hollow feet varus | No | Normal | c.(1721+1_1722-1)_(*1_?), p.?  Deletion (exons 16-58) | No |
| 26 | Familial | M | 46 | Suggestive | No | Yes (< 2 SD) | . Facial asymmetry  . Ptosis  . Hypertelorism  . Triangular face  . Down slanting palpebral fissures  . Low set posteriorly angulated ears  . Wide and prominent philtrum  . Prominent nasolabial folds | L, CLS, SNF (>2), SCNF | . Mild mental retardation  . Impairement of concentration | . Mitral valvular prolapse  . Ascending aorta dilation (TTE)  . ECG: normal | ND | Scoliosis | No | Hydrocephaly  Pheochromocytoma | c.2033dup, p.Ile679Aspfs*21 | No |
| Abbreviations: F, female; M, male; L, lentigines; CLS, café-au-lait spots; CT, computed tomography; ECG, electrocardiogram; FASI, focal areas of signal intensity; ND, not done; PNF, plexiform neurofibroma; RMI, resonance magnetic imaging; SCNF, subcutaneous neurofibromas; SD, standard deviation; SNF, superficial neurofibromas; TTE, transthoracic echocardiography.  * Lentigines (axillary and/or inguinal) and café-au-lait spots (over 5 mm in diameter in prepubertal individuals and over 15 mm in greatest diameter in postpubertal individuals) were consistently more than 2 and 5 in number, respectively.  **Including pathogenic variants of the following genes, involved in the RAS-MAPkinases pathway: *PTPN11*, *SOS1*, *SOS2*, *SHOC2*, *CBL*, *HRAS*, *NRAS*, *KRAS*, *RIT1*, *RRAS*, *RRAS2*, *BRAF*, *RAF1*, *MAP2K1*, *MAP2K2*, *SPRED1*, *SPRED2*, *NF1*, *PPP1CB*, and *LZTR1*. | | | | | | | | | | | | | | | | |
