# Supplementary material for: Neurofibromatosis-Noonan syndrome: a prospective monocentric study of 26 patients and literature review
Source: Orphanet J Rare Dis. 2025 Apr 27;20:201. doi: 10.1186/s13023-025-03706-3 (PMC12036184; doi:10.1186/s13023-025-03706-3)
Supplement: Supplementary file 3 — Supplementary Material 3 [file 13023_2025_3706_MOESM3_ESM.docx]

| **Table S3. Details of scoring facial Noonan-like phenotype of the 26 patients NF-NS with *NF1* pathogenic variants** | | | | | | | | | | | | | | | |
| --- | --- | --- | --- | --- | --- | --- | --- | --- | --- | --- | --- | --- | --- | --- | --- |
| Case number | Sex | Age (year) | Facial Noonan phenotype | Global score established by the team of geneticists and dermatologist | Face2Gene Gestalt analysis* | Score established by each geneticist and dermatologist | | | | | | |  |  |  |
|  |  |  |  |  |  | 1 | 2 | 3 | 4 | 5 | 6 | 7 | |  |  |
| 1 | M | 6 | . Coarse face  . Ptosis (bilateral)  . Downslanted palpebral fissures  . Epicanthal folds  . Bulbous nasal tip  . Wide prominent philtrum  . Micrognathia | 1  (suggestive) | 0  (low-suggestive) | 1  (suggestive) | 2  (typical) | 0  (low-suggestive) | 1  (suggestive) | 2  (typical) | 2  (typical) | 1  (suggestive) | |  |  |
| 2 | F | 45 | . Downslanted palpebral fissures  . Low-set posteriorly angulated ears  . Prominent nasolabial folds | 1  (suggestive) | 2  (typical) | 1  (suggestive) | 1  (suggestive) | 1  (suggestive) | 0  (low-suggestive) | 1  (suggestive) | 1  (suggestive) | 2  (typical) | |  |  |
| 3 | M | 8 | . Hypoplasia of the midface  . Triangular face  . Low-set posteriorly angulated ears  . High and broad nasal bridge  . Wide and prominent philtrum  . Wide peaks to vermillion border of the upper lip  . Low posterior hairline | 1  (suggestive) | 1  (suggestive) | 1  (suggestive) | 1  (suggestive) | 1  (suggestive) | 1  (suggestive) | 2  (typical) | 1  (suggestive) | 1  (suggestive) | |  |  |
| 4 | M | 12 | . Facial asymmetry  . Downslanted palpebral fissures  . Epicanthal folds  . Low-set posteriorly angulated ears  . Midface hypoplasia  . Wide and prominent philtrum | 1  (suggestive) | 1  (suggestive) | 1  (suggestive) | 1  (suggestive) | 0  (low-suggestive) | 1  (suggestive) | 1  (suggestive) | 1  (suggestive) | 1  (suggestive) | |  |  |
| 5 | F | 9 | . Prominent and high forehead  . Ptosis  . Hypertelorism  . Downslanted palpebral fissures  . Low-set posteriorly angulated ears | 2  (typical) | 2  (typical) | 2  (typical) | 2  (typical) | 2  (typical) | 1  (suggestive) | 2  (typical) | 2  (typical) | 2  (typical) | |  |  |
| 6 | M | 8 | . Facial asymmetry  . Ptosis  . Hypertelorism  . Downslanted palpebral fissures  . Eversion of the lateral eyelid  . Epicanthal folds  . Low-set posteriorly angulated ears  . Wide and prominent philtrum  . Micrognathia | 2  (typical) | 2  (typical) | 2  (typical) | 2  (typical) | 2  (typical) | 1  (suggestive) | 1  (suggestive) | 2  (typical) | 2  (typical) | |  |  |
| 7 | M | 9 | . Prominent and high forehead  . Hypertelorism  . Hypoplasia of the midface  . Downslanted palpebral fissures  . Epicanthal folds  . High and broad nasal bridge  . Bulbous nasal tip | 2  (typical) | 2  (typical) | 2  (typical) | 1  (suggestive) | 2  (typical) | 1  (suggestive) | 0  (low-suggestive) | 0  (low-suggestive) | 2  (typical) | |  |  |
| 8 | M | 8 | . Facial asymmetry  . Prominent and high forehead | 1  (suggestive) | 1  (suggestive) | 1  (suggestive) | 1  (suggestive) | 2  (typical) | 0  (low-suggestive) | 1  (suggestive) | 2  (typical) | 1  (suggestive) | |  |  |
| Abbreviations: F, female; M, male; ND, not done.  * Gestalt score confidence Face2Gene was defined as followed: 2 = “high confidence” (considered ‘typical’); 1 = “medium confidence” (considered ‘suggestive’, scored 1); 0 = “low confidence” (considered ‘low-suggestive’) | | | | | | | | | | | | | | |  |

| **Table S3. Details of scoring facial Noonan-like phenotype of the 26 patients NF-NS with *NF1* pathogenic variants (continued)** | | | | | | | | | | | | | | | |
| --- | --- | --- | --- | --- | --- | --- | --- | --- | --- | --- | --- | --- | --- | --- | --- |
| Case number | Sex | Age (year) | Facial Noonan phenotype | Global score established by the team of geneticists and dermatologist | Face2Gene Gestalt analysis* | Score established by each geneticist and dermatologist | | | | | | | |  |  |
|  |  |  |  |  |  | 1 | 2 | 3 | 4 | 5 | 6 | 7 | |  |  |
| 9 | F | 27 | . Prominent and high forehead  . High anterior hairline  . Hypertelorism  . Low-set posteriorly angulated ears  . Bulbous nasal tip | 1  (suggestive) | 1  (suggestive) | 1  (suggestive) | 1  (suggestive) | 2  (typical) | 0  (low-suggestive) | 1  (suggestive) | 1  (suggestive) | 1  (suggestive) | |  |  |
| 10 | M | 47 | . High anterior hairline  . Low-set posteriorly angulated ears  . Prominent nasolabial folds | 1  (suggestive) | 1  (suggestive) | 0  (low-suggestive) | 1  (suggestive) | 1  (suggestive) | 0  (low-suggestive) | 0  (low-suggestive) | 1  (suggestive) | 1  (suggestive) | |  |  |
| 11 | M | 10 | . Prominent and high forehead  . Frontal bossing  . Hypertelorism  . Triangular face  . Low-set posteriorly angulated ears  . Small and pointed chin | 2  (typical) | 2  (typical) | 2  (typical) | 1  (suggestive) | 2  (typical) | 0  (low-suggestive) | 1  (suggestive) | 2  (typical) | 2  (typical) | |  |  |
| 12 | M | 48 | . High anterior hairline  . Downslanted palpebral fissures  . Prominent nasal folds  . Bulbous nasal tip  . Wide peaks to vermillion border of the upper lip | 1  (suggestive) | 1  (suggestive) | 1  (suggestive) | 1  (suggestive) | 2  (typical) | 1  (suggestive) | 1  (suggestive) | 1  (suggestive) | 1  (suggestive) | |  |  |
| 13 | M | 10 | . Prominent and high forehead  . Frontal bossing  . Triangular face  . Midface hypoplasia  . High and broad nasal bridge  . Wide and prominent philtrum  . Small and pointed chin | 1  (suggestive) | 0  (low-suggestive) | 1  (suggestive) | 0  (low-suggestive) | 2  (typical) | 1  (suggestive) | 1  (suggestive) | 2  (typical) | 1  (suggestive) | |  |  |
| 14 | M | 7 | . Ptosis  . Downslanted palpebral fissures | 1  (suggestive) | 1  (suggestive) | 0  (low-suggestive) | 1  (suggestive) | 2  (typical) | 0  (low-suggestive) | 0  (low-suggestive) | 1  (suggestive) | 1  (suggestive) | |  |  |
| 15 | M | 3.5 | . Frontal bossing  . Hypertelorism  . Downslanted palpebral fissures  . Low-set posteriorly angulated ears | 2  (typical) | 2  (typical) | 2  (typical) | 1  (suggestive) | 2  (typical) | 0  (low-suggestive) | 1  (suggestive) | 1  (suggestive) | 2  (typical) | |  |  |
| 16 | M | 10 | . Facial asymmetry  . Epicanthus  . Downslanted palpebral fissures  . Thickened upper helix  . Micrognathia | 1  (suggestive) | 1  (suggestive) | 1  (suggestive) | 1  (suggestive) | 2  (typical) | 0  (low-suggestive) | 0  (low-suggestive) | 1  (suggestive) | 1  (suggestive) | | |  |
| 17 | F | 8 | . Triangular face  . Hypertelorism  . Downslanted palpebral fissures  . Epicanthus  . Wide and prominent philtrum  . Micrognathia | 1  (suggestive) | 1  (suggestive) | 1  (suggestive) | 0  (low-suggestive) | 2  (typical) | 0  (low-suggestive) | 0  (low-suggestive) | 1  (suggestive) | 1  (suggestive) | | |  |
| Abbreviations: F, female; M, male; ND, not done.  * Gestalt score confidence Face2Gene was defined as followed: 2 = “high confidence” (considered ‘typical’); 1 = “medium confidence” (considered ‘suggestive’, scored 1); 0 = “low confidence” (considered ‘low-suggestive’) | | | | | | | | | | | | |  |  |  |

| **Table S3. Details of baseline, facial Noonan phenotype, clinical manifestations and molecular characteristics of the 26 patients with NF1 pathogenic variants (end)** | | | | | | | | | | | | | | |
| --- | --- | --- | --- | --- | --- | --- | --- | --- | --- | --- | --- | --- | --- | --- |
| Case number | Sex | Age (year) | Facial Noonan phenotype | Global score established by the team of geneticists and dermatologist | Face2Gene Gestalt analysis* | Score established by each geneticist and dermatologist | | | | | | | |  |
|  |  |  |  |  |  | 1 | 2 | 3 | 4 | 5 | 6 | 7 |  |  |
| 18 | M | 9 | . Prominent and high forehead  . Ptosis  . Hypertelorism  . High and broad nasal bridge  . Hypoplasia of the midface  . Small and pointed chin | 2  (typical) | 2  (typical) | 2  (typical) | 1  (suggestive) | 2  (typical) | 1  (suggestive) | 1  (suggestive) | 2  (typical) | 2  (typical) |  |  |
| 19 | M (father of 18) | 45 | . Frontal bossing  . Hypertelorism  . Hypoplasia of the midface  . Low-set posteriorly angulated ears  . Prominent nasolabial folds | 1  (suggestive) | 1  (suggestive) | 0  (low-suggestive) | 1  (suggestive) | 2  (typical) | 0  (low-suggestive) | 1  (suggestive) | 0  (low-suggestive) | 1  (suggestive) |  |  |
| 20 | M (brother of 18) | 12 | . Ptosis  . Hypertelorism  . Down slanting palpebral fissures  . Low-set posteriorly angulated ears  . Wide and prominent philtrum  . Micrognathia | 2  (typical) | 1  (suggestive) | 1  (suggestive) | 2  (typical) | 2  (typical) | 1  (suggestive) | 1  (suggestive) | 2  (typical) | 2  (typical) |  |  |
| 21 | M | 19 | . Flat occiput  . Down slanting palpebral fissures  . Eversion of the lateral eyelid  . Low-set posteriorly angulated ears  . Wide and prominent philtrum | 1  (suggestive) | 0  (low-suggestive) | 1  (suggestive) | 1  (suggestive) | 0  (low-suggestive) | 0  (low-suggestive) | 1  (suggestive) | 2  (typical) | 1  (suggestive) |  |  |
| 22 | M | 14 | . Prominent and high forehead  . Ptosis  . Triangular face  . Low-set posteriorly angulated ears  . Wide and prominent philtrum | 2  (typical) | 1  (suggestive) | 2  (typical) | 0  (low-suggestive) | 2  (typical) | 1  (suggestive) | 1  (suggestive) | 2  (typical) | 2  (typical) |  |  |
| 23 | F | 11 | . Low-set posteriorly angulated ears  . High and broad nasal bridge  . Wide and prominent philtrum  . Micrognathia | 1  (suggestive) | 0  (low-suggestive) | 1  (suggestive) | 0  (low-suggestive) | 1  (suggestive) | ND | 1  (suggestive) | 1  (suggestive) | 0  (low-suggestive) |  |  |
| 24 | F | 1 | . Hypertelorism  . Low-set posteriorly angulated ears  . High and broad nasal bridge  . Triangular face  . Micrognathia | 1  (suggestive) | 1  (suggestive) | 1  (suggestive) | 1  (suggestive) | 2  (typical) | 2  (typical) | 0  (low-suggestive) | 1  (suggestive) | 2  (typical) |  |  |
| 25 | F | 18 | . Coarse face  . Ptosis  . Hypertelorism  . Bulbous nasal tip  . Wide and prominent philtrum  . Micrognathia | 1  (suggestive) | 1  (suggestive) | 1  (suggestive) | 2  (typical) | 2  (typical) | 0  (low-suggestive) | 1  (suggestive) | 2  (typical) | 1  (suggestive) |  |  |
| 26 | M | 46 | . Facial asymmetry  . Ptosis  . Hypertelorism  . Triangular face  . Down slanting palpebral fissures  . Low-set posteriorly angulated ears  . Wide and prominent philtrum  . Prominent nasolabial folds | 1  (suggestive) | 2  (typical) | 1  (suggestive) | 1  (suggestive) | 1  (suggestive) | ND | 1  (suggestive) | 1  (suggestive) | 1  (suggestive) |  |  |

Abbreviations: F, female; M, male; ND, not done.

* Gestalt score confidence Face2Gene was defined as followed: 2 = “high confidence” (considered ‘typical’); 1 = “medium confidence” (considered ‘suggestive’, scored 1); 0 = “low confidence” (considered ‘low-suggestive’)
