# Supplementary material for: Neurofibromatosis-Noonan syndrome: a prospective monocentric study of 26 patients and literature review
Source: Orphanet J Rare Dis. 2025 Apr 27;20:201. doi: 10.1186/s13023-025-03706-3 (PMC12036184; doi:10.1186/s13023-025-03706-3)
Supplement: Supplementary file 4 — Supplementary Material 4 [file 13023_2025_3706_MOESM4_ESM.docx]

**Table S4. Baseline characteristics and frequency of clinical manifestations and *NF1* pathogenic variants in our series in comparison with the data from the literature on neurofibromatosis type 1-Noonan syndrome and neurofibromatosis type 1.**

|  | Neurofibromatosis type 1-Noonan syndrome  Our study | Neurofibromatosis type 1-Noonan syndrome literature review^***^ | Neurofibromatosis type 1 literature review | Our study *vs* neurofibromatosis type 1-Noonan syndrome literature review  **(***P*-value) | Our study *vs* neurofibromatosis type 1 literature review  **(***P*-value) | Neurofibromatosis type 1-Noonan syndrome literature review *vs* neurofibromatosis type 1 literature review  (*P*-value) | Our study *vs* neurofibromatosis type 1-Noonan syndrome literature review (adjusted *P*-value) | Our study *vs* neurofibromatosis type 1 literature review  (adjusted *P*-value) | Neurofibromatosis type 1-Noonan syndrome literature review *vs* neurofibromatosis type 1 literature review  (adjusted *P*-value) |
| --- | --- | --- | --- | --- | --- | --- | --- | --- | --- |
| Baseline characteristics |  |  |  |  |  |  |  |  |  |
| Number of patients | 26 | 321 | - | - | - | - | - | - | - |
| Sex ratio (M/F) | 1.1 (19 /17) | 1.1 (159/142) | - | 0.996 | - | - | 1 | - | - |
| Facial Noonan phenotype | 100% (26/26) | 100% (242/242) | 3.1% (93/2978)^∞^ | 1 | **<0.001** | **<0.001** | 1 | **<0.001** | **<0.001** |
| Low-set and/or angulated ears | 61.5% (16/26) | 57.9% (140/242) | - | 0.717 | - | - | 0.925 | - | - |
| Down-slanting palpebral fissures | 53.8% (14/26) | 45.9% (111/242) | - | 0.438 | - | - | 0.704 | - | - |
| Hypertelorism | 50% (13/26) | 61.2% (148/242) | 52% (62/119)^∪^ | 0.270 | 0.846 | 0.101 | 0.470 | 1.000 | 0.114 |
| Ptosis | 37.5% (9/24^*^) | 40.1% (97/242) | 9.3% (7/75)^χ^ | 0.805 | **0.005** | **<0.001** | 1.000 | **0.028** | **<0.001** |
| Prominent and/or high forehead | 30.8% (8/26) | 10.7% (26/242) | - | **0.009** | - | - | 0.072 | - | **-** |
| Triangle-shaped head | 26.9% (7/26) | 4.5% (11/242) | - | **0.001** | - | - | **0.010** | - | **-** |
| Midface and/or malar hypoplasia | 23.1% (6/26) | 21.9% (53/242) | - | 0.891 | - | - | 1.000 | - | **-** |
| Epicanthal folds | 23.1% (6/26) | 14.5% (35/242) | - | 0.254 | - | - | 0.469 | - | **-** |
| Short stature | 23% (6/26) | 48.7% (134/275) | 17.4% (409/2346)^Ω^ | **0.012** | 0.437 | **<0.001** | 0.071 | 0.661 | **<0.001** |
| Macrocephaly | 42.3% (11/26) | 41.2 % (93/226) | 31.3% (787/2516)^∀^ | 0.910 | 0.228 | **0.002** | 1 | 0.443 | **0.003** |
| Cardiovascular abnormalities |  |  |  |  |  |  |  |  |  |
| Cardiovascular malformations**^§^** | 19.2% (5/26) | 36.8% (112/304) | 4% (121/3054)^†^ | 0.071 | 0.003 | **<0.001** | 0.162 | **0.019** | **<0.001** |
| Pulmonic stenosis | 7.7% (2/26) | 24.3% (74/304) | 1.7% (61/3680)^†^ | 0.053 | 0.071 | **<0.001** | 0.161 | 0.181 | **<0.001** |
| Left heart obstruction (aortic stenosis/coarctation) | 3.8% (1/26) | 2% (6/304) | 0.3% (10/3849)^†^ | 0.440 | 0.047 | **0.0006** | 0.668 | 0.139 | **<0.001** |
| Mitral valves prolapse/dysplasia | 7.7% (2/26) | 5.6% (17/304) | 1.1% (36/3236)^†^ | 0.653 | 0.036 | **<0.001** | 0.811 | 0.119 | **<0.001** |
| Atrial septal defect | 0% (0/26) | 4.3% (13/304) | 0.3% (13/4646)^†^ | 0.610 | 1 | **<0.001** | 0.807 | 1 | **<0.001** |
| Ventricular septal defect | 0% (0/26) | 2% (6/304) | 0.3% (11/4338)^†^ | 1 | 1 | **0.0005** | 1 | 1 | **<0.001** |
| Hypertrophic cardiomyopathy | 0% (0/26) | 2.6% (8/304) | 0.1% (4/2932)^†^ | 1 | 1 | **<0.001** | 1 | 1 | **<0.001** |
| Electrocardiographic abnormality | 8.7% (2/23) | 0.7% (2/304) | 0.2% (5/2322)^†^ | **0.026** | 0.002 | 0.190 | 0.103 | **0.012** | 0.202 |
| Vasculopathy |  |  |  |  |  |  |  |  |  |
| Cerebral vasculopathy | 3.8% (1/26) | 0% (0/305) | 4% (122/3024)° | 0.078 | 1 | **<0.001** | 0.162 | 1 | 0 |
| Peripheral vasculopathy | 3.8% (1/26) | 0.6% (2/321) | 2.5% (2/77)° | 0.209 | 1 | 0.170 | 0.372 | 1 | 0.186 |
| Skin manifestations |  |  |  |  |  |  |  |  |  |
| Café au lait spots (> 5) | 100% (26/26) | 96% (288/300) | 93.6% (3690/3943)^††^ | 0.609 | 0.407 | 0.095 | 0.807 | 0.661 | 0.112 |
| Skinfold freckling | 96.2% (25/26) | 64% (192/300) | 78.9% (3022/3831)^††^ | **0.0006** | 0.031 | **<0.001** | **0.004** | 0.114 | **<0.001** |
| Superficial cutaneous neurofibromas (> 18 y) | 62.5% (5/8) | 37.6% (32/85) | 91% (803/882)^††^ | 0.258 | 0.030 | **<0.001** | 0.423 | 0.114 | **<0.001** |
| Subcutaneous neurofibromas (> 18 y) | 50% (4/8) | 14.1% (12/85) | 57.7% (297/515)^††^ | **0.027** | 0.728 | **<0.001** | 0.103 | 0.961 | **<0.001** |
| Plexiform neurofibromas (major external/severe; > 8 y) | 37.5% (3/8) | 12.6% (23/183) | 20% (169/847)^††^ | 0.079 | 0.205 | **0.02** | 0.162 | 0.423 | **0.024** |

| **Table S4. Baseline characteristics and frequency of clinical manifestations and *NF1* pathogenic variants in our series in comparison with the data from the literature on neurofibromatosis type 1-Noonan syndrome and neurofibromatosis type 1.** | | | | | | | | | | | |
| --- | --- | --- | --- | --- | --- | --- | --- | --- | --- | --- | --- |
|  | Neurofibromatosis type 1-Noonan syndrome  Our study | Neurofibromatosis type 1-Noonan syndrome literature review* | Neurofibromatosis type 1 literature review | Our study *vs* neurofibromatosis type 1-Noonan syndrome literature review  **(***P*-value) | Our study *vs* neurofibromatosis type 1 literature review  **(***P*-value) | Neurofibromatosis type 1-Noonan syndrome literature review *vs* neurofibromatosis type 1 literature review  (*P*-value) | Our study *vs* neurofibromatosis type 1-Noonan syndrome literature review (adjusted *P*-value) | Our study *vs* neurofibromatosis type 1 literature review  (adjusted *P*-value) | Neurofibromatosis type 1-Noonan syndrome literature review *vs* neurofibromatosis type 1 literature review  (adjusted *P*-value) |  |  |
| Neurological manifestations |  |  |  |  |  |  |  |  |  |  |  |
| Learning disabilities | 15.4% (4/26) | 32.8% (98/299) | 24.5% (260/1063)^τ^ | 0.069 | 0.286 | **0.004** | 0.162 | 0.497 | **0.005** |  |  |
| Attention deficit hyperactivity disorders | 34.6% (9/26) | 7.4% (22/299) | 22.8% (227/996)^Ψ^ | **0.0002** | 0.158 | **<0.001** | **0.002** | 0.372 | **<0.001** |  |  |
| Clinical autism spectrum disorder | 0% (0/26) | 1% (3/299) | 7.8% (163/2077)^θ^ | 1 | 0.258 | **<0.001** | 1 | 0.473 | **<0.001** |  |  |
| Developmental delay/intellectual disability | 19.2% (5/26) | 25.8% (77/299) | 13.4% (154/1146)^ϖ^ | 0.463 | 0.383 | **<0.001** | 0.712 | 0.632 | **<0.001** |  |  |
| Nervous-system tumours |  |  |  |  |  |  |  |  |  |  |  |
| Optic pathway gliomas (MRI and/or CT-scan; < 6 years) | - | 20% (4/20) | 18% (102/566)^ϕ^ | - | - | 0.770 | - | - | 0.793 |  |  |
| Malignant peripheral nerve sheat tumours | 3.8% (1/26) | 0% (0/321) | 3.4 % (191/5682)^&^ | 0.075 | 0.590 | **<0.001** | 0.162 | 0.847 | **<0.001** |  |  |
| Lisch nodules (> 20 years) | 33.3% (1/3) | 51.4% (18/35) | 94% (102/108)^∃^ | 1 | 0.013 | **<0.001** | 1 | 0.062 | **<0.001** |  |  |
| Skeletal abnormalities |  |  |  |  |  |  |  |  |  |  |  |
| Scoliosis | 19.2% (5/26) | 23.2% (70/302) | 22.9% (240/1047)^#^ | 0.645 | 0.658 | 0.926 | 0.811 | 0.904 | 0.926 |  |  |
| Pectus excavatum | 61.5% (16/26) | 19.9% (57/287) | 1% (12/1157)^φ^ | **<0.001** | <0.001 | **<0.001** | **<0.001** | **<0.001** | **<0.001** |  |  |
| *NF1* pathogenic variant |  |  |  |  |  |  |  |  |  |  |  |
| Truncating | 34.6% (9/26) | 18.6% (54/280) | 54% (576/1067) | 0.064 | 0.050 | **<0.001** | 0.162 | 0.139 | **<0.001** |  |  |
| Missense | 38.5% (10/26) | 61.4% (172/280) | 9.2% (98/1067) | **0.022** | <0.001 | **<0.001** | 0.102 | **<0.001** | **<0.001** |  |  |
| Splice | 15.4% (4/26) | 3.2% (9/280) | 27.3% (291/1067) | **0.017** | 0.177 | **<0.001** | 0.089 | 0.390 | **<0.001** |  |  |
| In-frame | 0% (0/26) | 12.5% (35/280) | 2% (21/1067) | 0.055 | 1 | **<0.001** | 0.161 | 1 | **<0.001** |  |  |
| Large deletions | 11.5% (3/26) | 3.6% (10/280) | 7.5% (80/1067) | 0.088 | 0.441 | **0.019** | 0.172 | 0.661 | **0.024** |  |  |

**Abbreviations:** CT, computerized tomography; MRI, magnetic resonance imaging; -, not done.

^*^In two cases, the presence of a neurofibroma on the upper eyelid precluded the diagnosis of ptosis.

^**^ Other signs included: Coarse face, flat occiput/brachycephaly, facial asymmetry, eversion of the lateral eyelid, thickened lips, thickened upper helix, high and broad nasal bridge, depressed flat root, bulbous nasal tip, upturned nasal tip, wide and prominent philtrum, wide peaks to vermillion border of the upper lip

(cupid’s bow appearance), micrognathia, small, pointed chin, low posterior hairline, webbed neck

^***^ Literature review conducted up to December 2023 (See Supporting Information Table S1).

**^§^** Cardiovascular malformations are defined as a structural malformation involving intracardiac structures or great arteries.

^∞^ Based on: Friedman and Birch, Am J Med Genet 1997; Young *et al*, J Child Neurol 2002; Khosrotehrani *et al*. Arch Dermatol 2003; D’Amico *et al*, Clin Genet 2021.

^†^ Based on: Colley *et al* Clin Genet 1996; Lin *et al*, Am J Med Genet 2000; Lama *et al*, Pediatr Nephrol 2004; Incecik *et al*, Turk Kardiyol Dern Ars, 2015; Leppävirta J *et al*, Orphanet J Rare Diseases 2018; Pinna *et al*, Genes 2019; Tedesco *et al*, Am Heart J 2022.

° Based on: Rosser *et al*, Neurology 2005; Cairns *et al*, J Neurol Neurosurg Psychiatry 2008; Rea *et al*, Pediatrics 2009; Kaas *et al*, J Child Neurol 2013; Ghosh *et al*, J Child Neurol 2013; Paschoal *et al*, J Child Neurol 2016; Kang *et al*, J Hum Genet 2020; Sheerin *et al*, Am J Med Genet 2022.

^ϕ^ Based on: Obringer et al, 1989; Listernick *et al*, J Pediatr 1994; Blazo *et al*, Am J Med Genet 2004; Blanchard *et al*, 2016.

^&^ Based on: Huson *et al*, Brain 1988; Cnossen *et al*, Arch Dis Child 1998; King AA *et al*, Am J Med Genet 2000; Evans *et al*, J Med Genet 2002; Tucker *et al*, Neurology 2005; Darrigo *et al*, J Pediatr (Rio J) 2007; Alkindy *et al*, Hum Genomics 2012; Sabbagh *et al*, Hum Mut 2013; Tabata *et al*, JCI Insight 2020;

^††^ Based on: Young *et al*, J Child Neurol 2002; Khosrotehrani *et al*, Am J Med Genet 2005; Sabbagh *et al*, Hum Mut 2013; Duat Rodriguez *et al*, An Pediatr (Barc) 2015; Koczkowska *et al*, Hum Mutation 2020; Kang *et al*, J Hum Genet 2020; D’Amico *et al*, Clin Genet 2021; Almuqbil *et al*, J Multidiscip Healthc 2024.

^∃^ Based on : Lubs *et al*, N Engl J Med 1991 ; Beauchamp, Trans Am Ophthalmol Soc, 1995.

^#^ Based on : Wang *et al*, Eur Spine J 2024.

^φ^ Based on : Miraglia *et al*, Ital J Dermatol Venereol 2021.

^∀^ Based on : Huson *et al*, Brain 1988 ; Young *et al*, J Child Neurol 2002; Khosrotehrani *et al*. Arch Dermatol 2003; Barton *et al*, Dev Med Child Neurol 2004; Karvonen *et al*, Horm Res Paediatr 2013; Sabbagh *et al*, Hum Mut 2013; Pinna *et al*, Genes 2019; Kang *et al*, J Hum Genet 2020; Basto *et al*. Orphanet J Rare Dis 2022; Almuqbil *et al*, J Multidiscip Healthc 2024.

^Ω^ Based on : Huson *et al*, Brain 1988; Cnossen *et al*, J Med Genet 1998; Young *et al*, J Child Neurol 2002; Khosrotehrani *et al*. Arch Dermatol 2003; Pinna *et al*, Genes 2019; Kang *et al*, J Hum Genet 2020; D’Amico *et al*, Clin Genet 2021; Basto *et al*. Orphanet J Rare Dis 2022; Almuqbil *et al*, J Multidiscip Healthc 2024.

^θ^ Based on : Garg *et al*, Pediatrics 2013 ; Plasschaert *et al*, Am J Med Genet B 2015; Morris *et al*, JAMA Psychiatry 2016; Eijk *et al*, J Autism Dev Disord 2018; Morotti *et al*, Dev Med Child Neurol 2021.

^Ψ^ Based on: Barton and North, Dev Med Child Neurol 2004; Garg *et al*, Dev Med Child Neurol 2013; Walsh et al, Dev Med Child Neurol 2013; Kang *et al*, J Hum Genet 2020; Sanchez-Marco *et al*, Neurologia (Engl Ed) 2022; Almuqbil *et al*, J Multidiscip Healthc 2024.

^ϖ^ Based on: Huson *et al*, Brain 1988; Cnossen *et al*, Arch Dis Child 1998; Young *et al*, J Child Neurol 2002; D’Amico *et al*, Clin Genet 2021.

^τ^ Based on: McGaughan *et al*, J Med Genet 1999; Kang *et al*, J Hum Genet 2020; D’Amico *et al*, Clin Genet 2021.

^∪^ Based on: Westerhof *et al*, Arch Dermatol 1984; Cnossen *et al*, J Med Genet 1998.

^χ^ Based on: Hall. Am J Med Genet 1986.

**References**

1. Alkindy A, Chuzhanova N, Kini U, Cooper DN, Upadhyaya M. [Genotype-phenotype associations in neurofibromatosis type 1 (NF1): an increased risk of tumor complications in patients with NF1 splice-site mutations?](https://pubmed.ncbi.nlm.nih.gov/23244495/) Hum Genomics. 2012; 6:12.
2. Almuqbil M, Alshaikh FY, Altwaijri W *et al*. [Epidemiology and Outcomes of Neurofibromatosis Type 1 (NF-1): Multicenter Tertiary Experience.](https://pubmed.ncbi.nlm.nih.gov/38533410/)J Multidiscip Healthc. 2024; 17:1303-14.
3. Barton B, North K. [Social skills of children with neurofibromatosis type 1.](https://pubmed.ncbi.nlm.nih.gov/15287247/) Dev Med Child Neurol. 2004;46:553-63.
4. Basto DL, de Souza Vieira G, Andrade-Losso RM *et al*. [Head circumference and anthropometric changes and their relation to plexiform and skin neurofibromas in sporadic and familial neurofibromatosis 1 Brazilian adults: a cross-sectional study.](https://pubmed.ncbi.nlm.nih.gov/36064430/) Orphanet J Rare Dis. 2022; 17:341.
5. Beauchamp GR. [Neurofibromatosis type 1 in children.](https://pubmed.ncbi.nlm.nih.gov/8719691/) Trans Am Ophthalmol Soc. 1995;93:445-72.
6. Blanchard G, Lafforgue MP, Lion-François L *et al*. [Systematic MRI in NF1 children under six years of age for the diagnosis of optic pathway gliomas. Study and outcome of a French cohort.](https://pubmed.ncbi.nlm.nih.gov/26774135/) Eur J Paediatr Neurol. 2016; 20:275-81.
7. Blazo MA, Lewis RA, Chintagumpala MM *et al*. [Outcomes of systematic screening for optic pathway tumors in children with Neurofibromatosis Type 1.](https://pubmed.ncbi.nlm.nih.gov/15150770/) Am J Med Genet A. 2004; 127A:224-9.
8. Cairns AG, North KN. [Cerebrovascular dysplasia in neurofibromatosis type 1.](https://pubmed.ncbi.nlm.nih.gov/18469031/) J Neurol Neurosurg Psychiatry. 2008; 79:1165-70.
9. Cnossen MH, de Goede-Bolder A, van den Broek KM *et al*. [A prospective 10 year follow up study of patients with neurofibromatosis type 1.](https://pubmed.ncbi.nlm.nih.gov/9659085/) Arch Dis Child. 1998; 78:408-12.
10. Cnossen MH, Moons KG, Garssen MP *et al*. [Minor disease features in neurofibromatosis type 1 (NF1) and their possible value in diagnosis of NF1 in children < or = 6 years and clinically suspected of having NF1. Neurofibromatosis team of Sophia Children's Hospital.](https://pubmed.ncbi.nlm.nih.gov/9719365/) J Med Genet. 1998; 35:624-7.
11. Colley A, Donnai D, Evans DG. [Neurofibromatosis/Noonan phenotype: a variable feature of type 1 neurofibromatosis.](https://pubmed.ncbi.nlm.nih.gov/8740913/) Clin Genet. 1996; 49:59-64.
12. D'Amico A, Rosano C, Pannone L *et al*. [Clinical variability of neurofibromatosis 1: A modifying role of cooccurring PTPN11 variants and atypical brain MRI findings.](https://pubmed.ncbi.nlm.nih.gov/34346503/) Clin Genet. 2021; 100:563-72.
13. Darrigo LG Jr, Geller M, Bonalumi Filho A, Azulay DR. [Prevalence of plexiform neurofibroma in children and adolescents with type I neurofibromatosis.](https://pubmed.ncbi.nlm.nih.gov/18046492/) J Pediatr (Rio J). 2007; 83:571-3.
14. [Duat Rodríguez A, Martos Moreno GÁ, Martín Santo-Domingo Y *et al*. [Phenotypic and genetic features in neurofibromatosis type 1 in children].](https://pubmed.ncbi.nlm.nih.gov/25541118/) An Pediatr (Barc). 2015; 83:173-82.
15. Eijk S, Mous SE, Dieleman GC *et al*. [Autism Spectrum Disorder in an Unselected Cohort of Children with Neurofibromatosis Type 1 (NF1).](https://pubmed.ncbi.nlm.nih.gov/29423604/) J Autism Dev Disord. 2018; 48:2278-85.
16. Evans DG, Baser ME, McGaughran J *et al*. [Malignant peripheral nerve sheath tumours in neurofibromatosis 1.](https://pubmed.ncbi.nlm.nih.gov/12011145/) J Med Genet. 2002; 39:311-4.
17. Friedman JM, Birch PH. Type 1 neurofibromatosis: a descriptive analysis of the disorder in 1,728 patients. Am J Med Genet. 1997; 70:138-43.
18. Garg S, Lehtonen A, Huson SM *et al*. [Autism and other psychiatric comorbidity in neurofibromatosis type 1: evidence from a population-based study.](https://pubmed.ncbi.nlm.nih.gov/23163236/) Dev Med Child Neurol. 2013; 55:139-145.
19. Garg S, Green J, Leadbitter K *et al*. [Neurofibromatosis type 1 and autism spectrum disorder.](https://pubmed.ncbi.nlm.nih.gov/24190681/) Pediatrics. 2013; 132: e1642-8.
20. Ghosh PS, Rothner AD, Emch TM *et al*. [Cerebral vasculopathy in children with neurofibromatosis type 1.](https://pubmed.ncbi.nlm.nih.gov/22532547/) J Child Neurol. 2013; 28:95-101.
21. Hall BD. [Congenital lid ptosis associated with neurofibromatosis.](https://pubmed.ncbi.nlm.nih.gov/3098101/) Am J Med Genet. 1986; 25:595-7.
22. Huson SM, Harper PS, Compston DA. [Von Recklinghausen neurofibromatosis. A clinical and population study in south-east Wales.](https://pubmed.ncbi.nlm.nih.gov/3145091/) Brain. 1988; 111:1355-81.
23. İncecik F, Hergüner ÖM, Alınç Erdem S, Altunbaşak Ş. [Neurofibromatosis type 1 and cardiac manifestations.](https://pubmed.ncbi.nlm.nih.gov/26717333/) Turk Kardiyol Dern Ars. 2015; 43:714-6.
24. Kang E, Kim YM, Seo GH *et al*. [Phenotype categorization of neurofibromatosis type I and correlation to NF1 mutation types.](https://pubmed.ncbi.nlm.nih.gov/31776437/) J Hum Genet. 2020; 65:79-89.
25. Kaas B, Huisman TA, Tekes A *et al*. [Spectrum and prevalence of vasculopathy in pediatric neurofibromatosis type 1.](https://pubmed.ncbi.nlm.nih.gov/22832780/) J Child Neurol. 2013; 28:561-9.
26. Karvonen M, Saari A, Hannila ML *et al*. [Elevated head circumference-to-height ratio is an early and frequent feature in children with neurofibromatosis type 1.](https://pubmed.ncbi.nlm.nih.gov/23466600/) Horm Res Paediatr. 2013; 79:97-102.
27. Khosrotehrani K, Bastuji-Garin S, Zeller J *et al*. [Clinical risk factors for mortality in patients with neurofibromatosis 1: a cohort study of 378 patients.](https://pubmed.ncbi.nlm.nih.gov/12588224/) Arch Dermatol. 2003; 139:187-91.
28. Khosrotehrani K, Bastuji-Garin S, Riccardi VM *et al*. [Subcutaneous neurofibromas are associated with mortality in neurofibromatosis 1: a cohort study of 703 patients.](https://pubmed.ncbi.nlm.nih.gov/15523617/) Am J Med Genet A. 2005; 132A:49-53.
29. King AA, Debaun MR, Riccardi VM, Gutmann DH. [Malignant peripheral nerve sheath tumors in neurofibromatosis 1.](https://pubmed.ncbi.nlm.nih.gov/10951462/) Am J Med Genet. 2000; 93:388-92.
30. Koczkowska M, Callens T, Chen Y *et al*. [Clinical spectrum of individuals with pathogenic NF1 missense variants affecting p.Met1149, p.Arg1276, and p.Lys1423: genotype-phenotype study in neurofibromatosis type 1.](https://pubmed.ncbi.nlm.nih.gov/31595648/) Hum Mutat. 2020; 41:299-315.
31. Lama G, Graziano L, Calabrese E *et al*. [Blood pressure and cardiovascular involvement in children with neurofibromatosis type1.](https://pubmed.ncbi.nlm.nih.gov/14991390/) Pediatr Nephrol. 2004; 19:413-8.
32. Leppävirta J, Kallionpää RA, Uusitalo E *et al*. [Congenital anomalies in neurofibromatosis 1: a retrospective register-based total population study.](https://pubmed.ncbi.nlm.nih.gov/29335026/) Orphanet J Rare Dis. 2018; 13:5.
33. Lin AE, Birch PH, Korf BR *et al*. [Cardiovascular malformations and other cardiovascular abnormalities in neurofibromatosis 1.](https://pubmed.ncbi.nlm.nih.gov/11078559/) Am J Med Genet. 2000; 95:108-17.
34. Listernick R, Charrow J, Greenwald M, Mets M. [Natural history of optic pathway tumors in children with neurofibromatosis type 1: a longitudinal study.](https://pubmed.ncbi.nlm.nih.gov/8021787/) J Pediatr. 1994; 125:63-6.
35. Lubs ML, Bauer MS, Formas ME, Djokic B. [Lisch nodules in neurofibromatosis type 1.](https://pubmed.ncbi.nlm.nih.gov/1901624/) N Engl J Med. 1991; 324:1264-6.
36. McGaughran JM, Harris DI, Donnai D *et al*. [A clinical study of type 1 neurofibromatosis in north west England.](https://pubmed.ncbi.nlm.nih.gov/10204844/) J Med Genet 1999; 36:197-203.
37. Miraglia E, Chello C, Calvieri S, Giustini S. [Pectus excavatum in neurofibromatosis type 1: a single-center experience.](https://pubmed.ncbi.nlm.nih.gov/31578835/) Ital J Dermatol Venerol. 2021; 156(Suppl. 1):88-9.
38. Morotti H, Mastel S, Keller K *et al*. [Autism and attention-deficit/hyperactivity disorders and symptoms in children with neurofibromatosis type 1.](https://pubmed.ncbi.nlm.nih.gov/32406525/) Dev Med Child Neurol. 2021; 63:226-32.
39. Morris SM, Acosta MT, Garg S *et al*. [Disease Burden and Symptom Structure of Autism in Neurofibromatosis Type 1: A Study of the International NF1-ASD Consortium Team (INFACT).](https://pubmed.ncbi.nlm.nih.gov/27760236/) JAMA Psychiatry. 2016; 73:1276-84.
40. Obringer AC, Meadows AT, Zackai EH. [The diagnosis of neurofibromatosis-1 in the child under the age of 6 years.](https://pubmed.ncbi.nlm.nih.gov/2499182/) Am J Dis Child. 1989; 143:717-9.
41. Paschoal JK, Paschoal FM Jr, de Lima FT *et al*. [Detection of Cerebral Vasculopathy by Transcranial Doppler in Children With Neurofibromatosis Type 1.](https://pubmed.ncbi.nlm.nih.gov/26184486/) J Child Neurol. 2016; 31:351-6.
42. Pinna V, Daniele P, Calcagni G *et al*. [Prevalence, Type, and Molecular Spectrum of NF1 Mutations in Patients with Neurofibromatosis Type 1 and Congenital Heart Disease.](https://pubmed.ncbi.nlm.nih.gov/31487937/) Genes (Basel). 2019; 10:675.
43. Plasschaert E, Descheemaeker MJ, Van Eylen L *et al*. [Prevalence of Autism Spectrum Disorder symptoms in children with neurofibromatosis type 1.](https://pubmed.ncbi.nlm.nih.gov/25388972/) Am J Med Genet B Neuropsychiatr Genet. 2015; 168B(1):72-80.
44. Rea D, Brandsema JF, Armstrong D *et al*. [Cerebral arteriopathy in children with neurofibromatosis type 1.](https://pubmed.ncbi.nlm.nih.gov/19706560/) Pediatrics. 2009; 124:e476-83.
45. Rosser TL, Vezina G, Packer RJ. [Cerebrovascular abnormalities in a population of children with neurofibromatosis type 1.](https://pubmed.ncbi.nlm.nih.gov/15699396/) Neurology. 2005; 64:553-5.
46. Sabbagh A, Pasmant E, Imbard A *et al*. [NF1 molecular characterization and neurofibromatosis type I genotype-phenotype correlation: the French experience.](https://pubmed.ncbi.nlm.nih.gov/23913538/) Hum Mutat. 2013; 34:1510-8.
47. Sánchez Marco SB, López Pisón J, Calvo Escribano C *et al*. [Neurological manifestations of neurofibromatosis type 1: our experience.](https://pubmed.ncbi.nlm.nih.gov/35672119/) Neurologia (Engl Ed). 2022; 37:325-33.
48. Sheerin UM, Holmes P; London NF1 Research Group *et al*. [Neurovascular complications in adults with Neurofibromatosis type 1: A national referral center experience.](https://pubmed.ncbi.nlm.nih.gov/36097643/) Am J Med Genet A. 2022; 188:3009-15.
49. Tabata MM, Li S, Knight P *et al*. [Phenotypic heterogeneity of neurofibromatosis type 1 in a large international registry.](https://pubmed.ncbi.nlm.nih.gov/32814709/) JCI Insight. 2020; 5:e136262.
50. Tedesco MA, Di Salvo G, Natale F *et al.* [The heart in neurofibromatosis type 1: an echocardiographic study.](https://pubmed.ncbi.nlm.nih.gov/12040353/) Am Heart J. 2002; 143:883-8.
51. Tucker T, Wolkenstein P, Revuz J *et al*. [Association between benign and malignant peripheral nerve sheath tumors in NF1.](https://pubmed.ncbi.nlm.nih.gov/16043787/) Neurology. 2005; 65:205-11.
52. Walsh KS, Vélez JI, Kardel PG *et al*. [Symptomatology of autism spectrum disorder in a population with neurofibromatosis type 1.](https://pubmed.ncbi.nlm.nih.gov/23163951/) Dev Med Child Neurol. 2013; 55:131-8.
53. Wang D, Zhang BH, Wen X *et al*. [Clinical features and surgical treatments of scoliosis in neurofibromatosis type 1: a systemic review and meta-analysis.](https://pubmed.ncbi.nlm.nih.gov/38526628/) Eur Spine J. 2024 Mar 25.
54. Westerhof W, Delleman JW, Wolters E, Dijkstra P. [Neurofibromatosis and hypertelorism.](https://pubmed.ncbi.nlm.nih.gov/6439124/) Arch Dermatol. 1984; 120:1579-81.
55. Young H, Hyman S, North K. [Neurofibromatosis 1: clinical review and exceptions to the rules.](https://pubmed.ncbi.nlm.nih.gov/12403560/) J Child Neurol. 2002; 17:613-21.
