# Supplementary material for: Neurofibromatosis-Noonan syndrome: a prospective monocentric study of 26 patients and literature review
Source: Orphanet J Rare Dis. 2025 Apr 27;20:201. doi: 10.1186/s13023-025-03706-3 (PMC12036184; doi:10.1186/s13023-025-03706-3)
Supplement: Supplementary file 5 — Supplementary Material 5 [file 13023_2025_3706_MOESM5_ESM.docx]

**Supplemental Figure 1 A-D. Concordance analysis of Noonan syndrome-like facial phenotype**


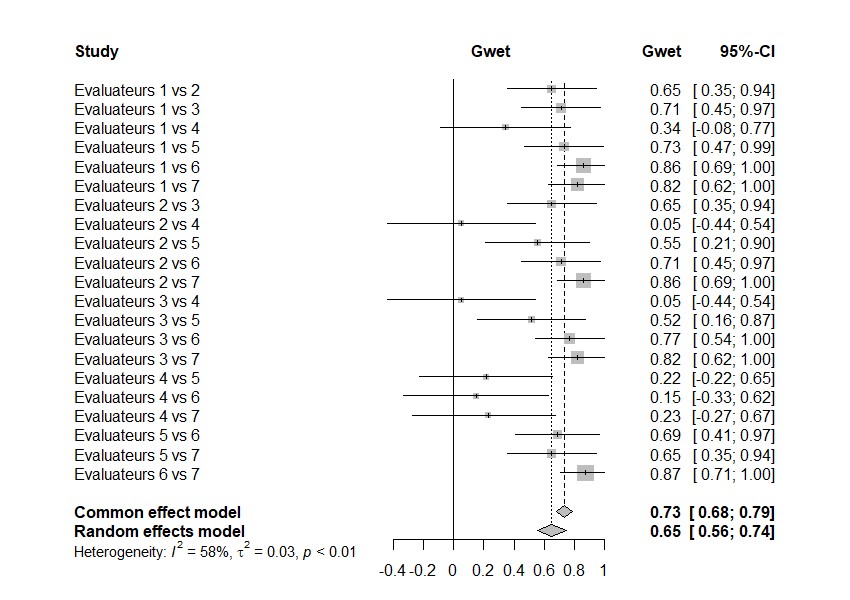


1. **Inter-rater concordance analysis of Noonan syndrome-like facial phenotype: suggestive or typical *versus* low-suggestive. The concordance was 0.65 [0.56; 0.74].**


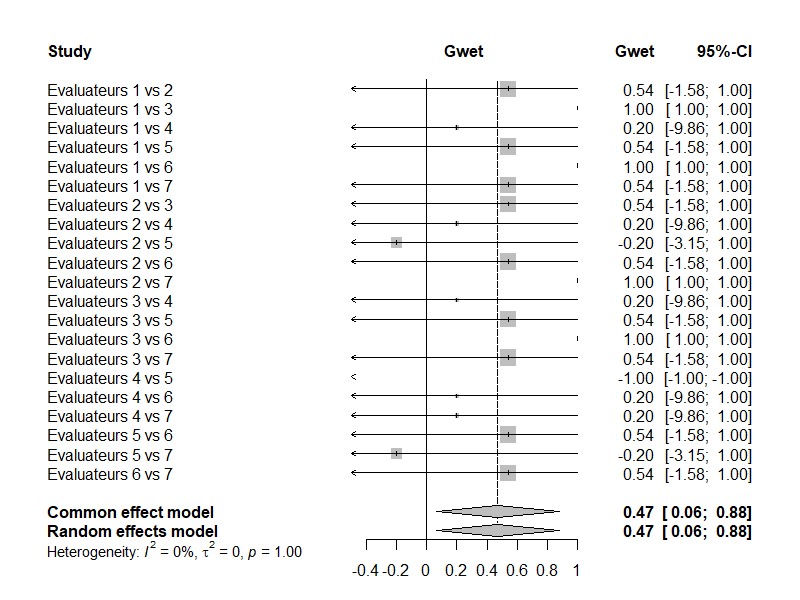


1. **Patients under12 years (3 patients) - Inter-rater concordance analysis of Noonan syndrome-like facial phenotype: suggestive/typical *versus* low-suggestive. The concordance was 0.47 [0.06; 0.88].**


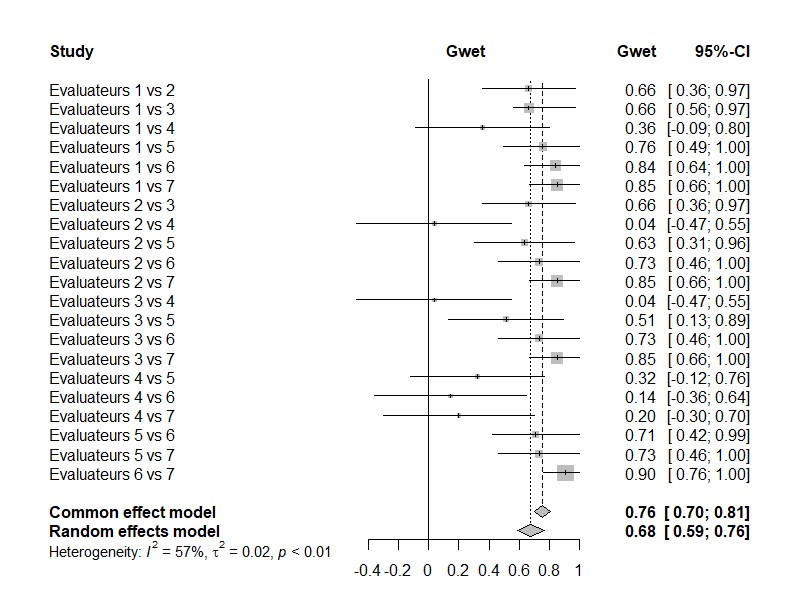


1. **Patients of 12 years and older (23 patients) - Inter-rater concordance analysis of Noonan syndrome-like facial phenotype: suggestive/typical *versus* low-suggestive. The concordance was 0.68 [0.59; 0.76].**


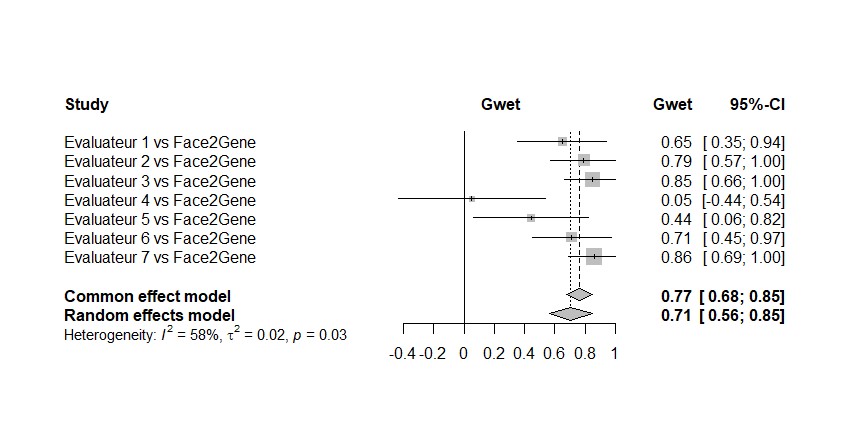


1. **Concordance between the clinicians’ panel average rating and the Face2Gene analysis of Noonan syndrome-like facial phenotype: suggestive or typical *versus* low-suggestive. The concordance was 0.821 [IC95% = 0.625; 1.000].**
